# Rectal Culture-Based Versus Empirical Antibiotic Prophylaxis to Prevent Infectious Complications in Men Undergoing Transrectal Prostate Biopsy: A Randomized, Nonblinded Multicenter Trial

**DOI:** 10.1093/cid/ciac913

**Published:** 2022-11-24

**Authors:** Sofie C M Tops, Eva Kolwijck, Evert L Koldewijn, Diederik M Somford, Filip J M Delaere, Menno A van Leeuwen, Anthonius J Breeuwsma, Thijn F de Vocht, Hans J H P Broos, Rob A Schipper, Martijn G Steffens, Steven Teerenstra, Marjolijn C A Wegdam-Blans, Els de Brauwer, Wouter van den Bijllaardt, Alexander C A P Leenders, J P Michiel Sedelaar, Heiman F L Wertheim

**Affiliations:** Department of Medical Microbiology and Radboudumc Center for Infectious Diseases, Radboud University Medical Center, Nijmegen, The Netherlands; Department of Medical Microbiology, Jeroen Bosch Hospital, ‘s-Hertogenbosch, The Netherlands; Department of Urology, Catharina Hospital, Eindhoven, The Netherlands; Department of Urology, Canisius Wilhelmina Hospital, Nijmegen, The Netherlands; Department of Urology, Zuyderland, Heerlen, The Netherlands; Department of Urology, Bravis, Roosendaal, The Netherlands; ETZ hospital, Tilburg, The Netherlands; Department of Urology, Bernhoven, Uden, The Netherlands; Department of Urology, Amphia, Breda, The Netherlands; Department of Urology, Jeroen Bosch Hospital, ‘s-Hertogenbosch, The Netherlands; Department of Urology, Isala, Zwolle, The Netherlands; Department for Health Evidence, Section Biostatistics, Radboud University Medical Center, Nijmegen, The Netherlands; Department of Medical Microbiology, University Medical Center Utrecht, Utrecht University, Utrecht, The Netherlands; Department of Medical Microbiology, Zuyderland, Heerlen, The Netherlands; Microvida Laboratory for Medical Microbiology, Amphia Hospital, Breda, The Netherlands; Department of Medical Microbiology, Jeroen Bosch Hospital, ‘s-Hertogenbosch, The Netherlands; Department of Urology, Radboud University Medical Center, Nijmegen, The Netherlands; Department of Medical Microbiology and Radboudumc Center for Infectious Diseases, Radboud University Medical Center, Nijmegen, The Netherlands

**Keywords:** culture-based antibiotic prophylaxis, empirical antibiotic prophylaxis, infectious complications, transrectal prostate biopsy

## Abstract

**Background:**

An increase in infections after transrectal prostate biopsy (PB), related to an increasing number of patients with ciprofloxacin-resistant rectal flora, necessitates the exploration of alternatives for the traditionally used empirical prophylaxis of ciprofloxacin. We compared infectious complication rates after transrectal PB using empirical ciprofloxacin prophylaxis versus culture-based prophylaxis.

**Methods:**

In this nonblinded, randomized trial, between 4 April 2018 and 30 July 2021, we enrolled 1538 patients from 11 Dutch hospitals undergoing transrectal PB. After rectal swab collection, patients were randomized 1:1 to receive empirical prophylaxis with oral ciprofloxacin (control group [CG]) or culture-based prophylaxis (intervention group [IG]). Primary outcome was any infectious complication within 7 days after biopsy. Secondary outcomes were infectious complications within 30 days, and bacteremia and bacteriuria within 7 and 30 days postbiopsy. For primary outcome analysis, the χ^2^ test stratified for hospitals was used. Trial registration number: NCT03228108.

**Results:**

Data from 1288 patients (83.7%) were available for analysis (CG, 652; IG, 636). Infection rates within 7 days postbiopsy were 4.3% (n = 28) (CG) and 2.5% (n = 16) (IG) (*P* value = .08; reduction: −1.8%; 95% confidence interval, −.004 to .040). Ciprofloxacin-resistant bacteria were detected in 15.2% (n = 1288). In the CG, the presence of ciprofloxacin-resistant rectal flora resulted in a 6.2-fold higher risk of early postbiopsy infection.

**Conclusions:**

Our study supports the use of culture-based prophylaxis to reduce infectious complications after transrectal PB. Despite adequate prophylaxis, postbiopsy infections can still occur. Therefore, culture-based prophylaxis must be weighed against other strategies that could reduce postbiopsy infections.

**Clinical Trials Registration**. NCT03228108.

## Teaser

Our study supports using culture-based prophylaxis to reduce infectious complications after transrectal prostate biopsy, especially with regard to the early, more severe infections. Despite adequate prophylaxis, patients can still develop postbiopsy infection; therefore, other strategies need to be explored as well.

Prostate biopsy (PB) is commonly performed using a transrectal ultrasound-guided approach. Unfortunately, transrectal PB may cause infections from introduction of enteric bacteria, particularly Enterobacterales such as *Escherichia coli*, into the urinary tract, prostate, or bloodstream [1]. To prevent these infectious complications, antibiotic prophylaxis is administered [2].

Traditionally, fluoroquinolones (FQs) are used as prophylaxis because of their coverage against common causative bacteria of postbiopsy infections and favorable prostatic penetration [2]. In recent years, a rise in FQ-resistant Enterobacterales [3, 4] has caused an up to 6% increase in postbiopsy infections [5–7]. Van Besien et al showed a 5-fold higher risk of postbiopsy infection using FQ prophylaxis in the presence of FQ-resistant rectal flora (7.9% vs 1.6%) [8]. Moreover, it was estimated that in the United States, 13.120 postbiopsy infections per year are attributable to FQ-resistant pathogens (42%), leading to a relevant burden on healthcare facilities [9].

A plausible strategy to overcome the problem of postbiopsy infections related to FQ-resistant Enterobacterales is rectal culture–based antibiotic prophylaxis [10, 11]. The strategy has the potential to limit selection of antibiotic resistance in contrast to other proposed strategies as augmented empirical prophylaxis consisting of a combination of antibiotics. Additionally, the presumed reduction of infections with culture-based prophylaxis will diminish the use of therapeutic antibiotics postbiopsy and therefore will not further drive development of antibiotic resistance.

Previous studies reported conflicting results regarding the impact of rectal culture–based prophylaxis on postbiopsy infection rates [10–14]. Until now, effectiveness has not been evaluated in a prospective randomized trial (RCT) with sufficient power. We performed a multicenter RCT to compare infectious complications rates after transrectal PB using empirical prophylaxis with ciprofloxacin versus rectal culture–based antibiotic prophylaxis.

## METHODS

### Study Design

This nonblinded, randomized trial was performed in 11 Dutch hospitals: 1 academic hospital, 8 nonacademic teaching hospitals, and 2 nonacademic nonteaching hospitals. The study was approved by the Medical Research Ethics Committee Nijmegen, all 11 institutional review boards, and underwent an extramarginal review by the Central Committee on Research Involving Human Subjects. Trial registration was performed prospectively (NCT03228108). The study was monitored by an independent expert. The study protocol including amendments summary is available in Supplementary File 1.

### Participants

All patients undergoing transrectal PB were eligible for inclusion. Patients were recruited between April 2018 and July 2021. Written informed consent was obtained. Exclusion criteria were: (1) contraindication for ciprofloxacin; (2) contraindication for selected prophylactic antibiotics used as alternatives for ciprofloxacin; (3) urinary tract infection (UTI) or acute prostatitis within 14 days before biopsy; (4) antibiotic use after rectal swab collection and before PB; (5) absence of (reliable) rectal swab to guide prophylaxis; (6) repeat PB within 7 days; and (7) inability to understand the nature of the trial and procedures required.

### Rectal Swab Specimens

After informed consent, a rectal swab was collected from all patients by a healthcare provider when PB was recommended or by self-sampling [15]. The swab was collected preferably at least 7 days before PB to ensure adequate time for results and earlier than 60 days before PB to ensure representative culture results. Immediately before PB, healthcare providers collected a second rectal swab. In case of infectious complications, this swab was used to provide information about the susceptibility of the rectal flora at the time of PB. See Supplementary File 2 for detailed information about the culture protocol [16].

### Randomization and Masking

After prebiopsy rectal swab collection, patients were randomly assigned to receive either empirical prophylaxis with ciprofloxacin (control group [CG]) or culture-based antibiotic prophylaxis (intervention group [IG]). Randomization was performed by the coordinating investigator using a web-based program (CastorEDC, Amsterdam, the Netherlands) with 1:1 allocation ratio and randomly selected block sizes of 4, 6, and 8 stratified for hospital and PB technique. The coordinating investigator was aware of the culture results, treatment allocation, and prophylaxis schedule on the patient level. Healthcare providers were aware of the latter 2. The antibiotic prophylaxis regimen was not blinded for patients. Outcomes were blinded until final analysis.

### Procedures

**Figure 1.**
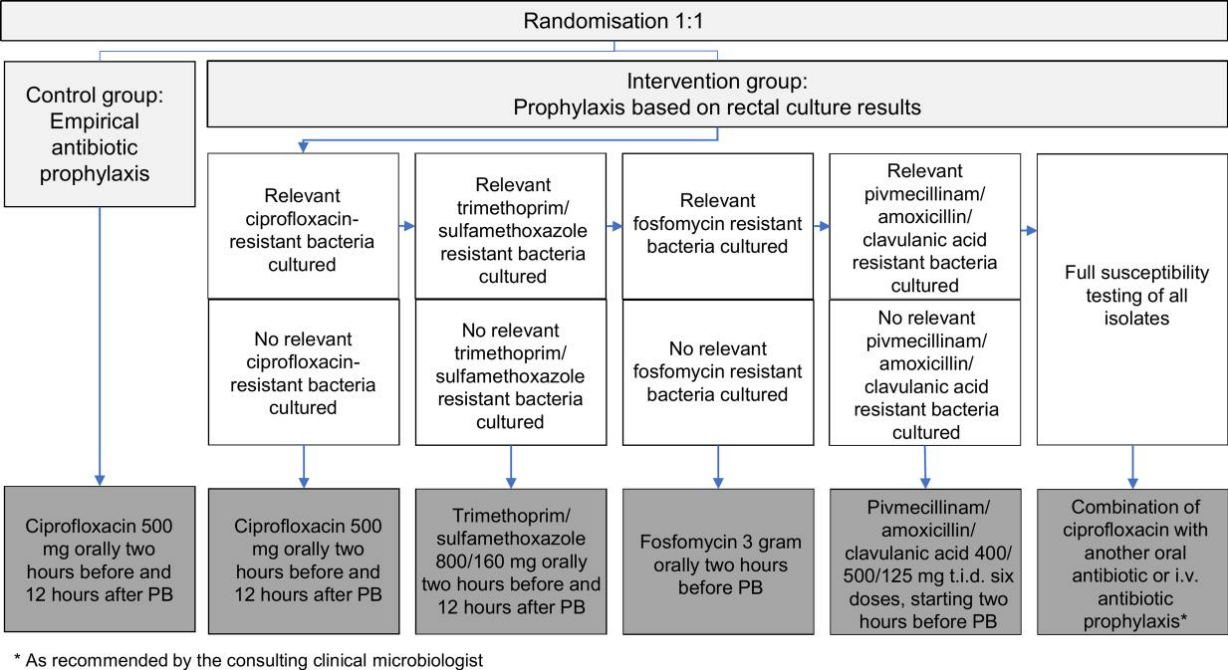
Flowchart of the antibiotic prophylaxis regimens prescribed per group.

A flowchart of the antibiotic prophylactic regimens per group is depicted in Figure 1. Patients were instructed to contact their urologist in case of signs and/or symptoms of infection (standard of care). The attending physician interpreted patient's symptoms and assessed whether additional diagnostic testing and/or treatment was necessary.

### Outcomes

Data on exclusion criteria, demographic characteristics, outcomes, and potential confounders were collected. Data were primary retrieved from the hospital's electronic medical record, including hospital outpatient, inpatient, and emergency department visits. Additionally, patient questionnaires were sent before PB, and approximately 7 and 30 days afterward (Supplementary File 3). If questionnaires were incomplete, patients were called to obtain the information. In this way, we ensured that no infectious events were missed (eg, infections that could not be withdrawn from the hospital's electronic medical record such as those treated by the general practitioner or in nonparticipating hospitals). In these cases, written medical information was retrieved. In case of infection, patients were called for additional information, blinded for the arm.

Primary outcome was any infectious complication within 7 days after PB (Table 1). Secondary outcomes were infectious complications within 30 days, and bacteremia and bacteriuria within 7 and 30 days postbiopsy. Moreover, hospitalization, overall mortality, antibiotic use, adverse events of antibiotics, and prevalence of ciprofloxacin-resistant Gram-negative bacilli in blood or urine cultures related to postbiopsy infections were recorded. Outcomes were centrally assessed.

**Table 1.** Definitions of Postbiopsy Infectious Complications

Definitive urinary tract infection	Without systemic symptoms	Symptoms of dysuria, urgency, frequency or hematuria, AND pyuria (>5 white blood cells per high-power field; >25 white blood cells/µL; or urinary dipstick test that is positive for leukocyte esterase) and/or bacteriuria (≥10^3^ colony-forming units/mL)
	With systemic symptoms^a^	Symptoms of dysuria, urgency, frequency or hematuria, AND/OR symptoms of fever, chills, or malaise AND pyuria (>5 white blood cells per high-power field; >25 white blood cells/µL; or urinary dipstick test that is positive for leukocyte esterase) and/or bacteriuria (≥10^3^ colony-forming units/mL)
Probable urinary tract infection	Without systemic symptoms	Symptoms of dysuria, urgency, frequency, or hematuria not proven by urine screening, sediment, or culture for which antibiotics are prescribed.
	With systemic symptoms	Symptoms of dysuria, urgency, frequency, or hematuria AND symptoms of fever, chills, or malaise not proven by urine screening, sediment or culture for which antibiotics are prescribed.
Acute prostatitis	…	Symptoms of fever, chills, malaise, dysuria, urgency, frequency, AND pelvic/perineal pain or tender prostate during palpitation of the prostate AND pyuria (>5 white blood cells per high power field; >25 white blood cells/µL; or urinary dipstick test that is positive for leukocyte esterase) and/or bacteriuria (≥10^3^ colony-forming units/mL)
Acute epididymitis	…	Presence of a swollen, red or warm scrotum, accompanied by tenderness for which antibiotics are prescribed.
Sepsis	…	Suspicion of infection plus at least 2 of the following criteria (quick sequential organ failure assessment score): systolic blood pressure ≤100 mmHg, respiratory rate ≥22 breaths/min, or Glasgow coma scale <15.
Severe sepsis	…	Sepsis plus organ dysfunction or with persisting hypotension requiring vasopressors to maintain a mean arterial pressure ≥65 mmHg and to have a serum lactate level <2 mmol/L despite adequate volume resuscitation.
Isolated fever	…	Body temperature ≥38.0 °C without localizing signs and symptoms

The primary outcome includes all of the types of infections that occurred within 7 days after prostate biopsy.

Systemic symptoms are defined as: fever, chills, malaise.

### Statistical Analysis

Sample size calculation was based on a 3.2% estimated infection rate within 7 days postbiopsy using empirical prophylaxis (CG) and 1.0% using culture-based prophylaxis (IG) [11, 12]. For 80% power, 5% 2-sided significance level, 1332 patients were required.

Characteristics of individuals in the 2 different groups were assessed for clinically relevant differences in prognostic factors. Distribution of continuous data was described by median and interquartile ranges (IQR) and categorical data by number and percentage. A modified intention-to-treat analysis was performed for the primary outcome using the χ^2^ test stratified for hospitals (Cochran–Mantel–Haenszel test). Descriptive statistical analysis was performed for secondary and exploratory outcomes. Point estimates of effects with 95% confidence intervals (CIs) for the difference between CG and IG (unstratified for hospital) were calculated using the Newcombe method [17]. For statistical analysis, SPSS Statistics for Windows, version 25.0 (Armonk, NY, IBM Corp), was used.

## RESULTS

### Study Population

**Figure 2.**
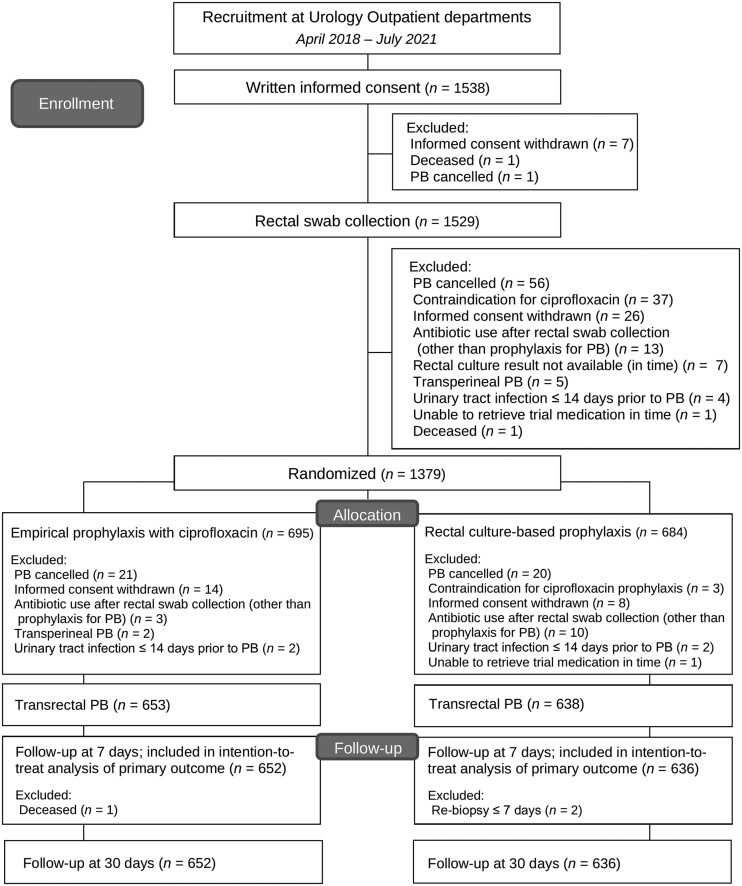
Flowchart of patient inclusion and exclusion.

In total, 1538 men were included between 4 April 2018 and 30 July 2021. The trial was finished because sufficient patients were recruited. For various reasons, 250 patients did not complete the entire study (16.3%); therefore, 1288 patients were included in the final analysis (Figure 2). Eighteen patients participated twice for different PB sessions.

Patients' characteristics per group can be found in Table 2. Despite randomization, an unequal distribution of clinically relevant prognostic baseline variables was observed, namely (1) diabetes mellitus (CG, 7.5%; IG, 11.2%); (2) indwelling catheter or intermittent catheterization (CG, 3.4%; IG, 1.9%) (Table 2). Six patients allocated to the IG (0.9%) group with resistant rectal Gram-negative bacilli mistakenly used the standard prophylaxis. One of these patients had an infection 10 days postbiopsy.

**Table 2.** Patients' Characteristics Per Group

	Total	Empirical Prophylaxis	Culture-based Prophylaxis	*P* Value for Difference
Number of patients, n (%)	1288	652	636	1.00
ȃHospital A	36 (2.8)	18 (2.8)	18 (2.8)	
ȃHospital B	399 (31.0)	206 (31.6)	193 (30.3)	
ȃHospital C	111 (8.6)	56 (8.6)	55 (8.6)	
ȃHospital D	72 (5.6)	35 (5.4)	37 (5.8)	
ȃHospital E	39 (3.0)	19 (2.9)	20 (3.1)	
ȃHospital F	54 (4.2)	26 (4.0)	28 (4.4)	
ȃHospital G	350 (27.2)	179 (27.5)	171 (26.9)	
ȃHospital H	56 (4.3)	28 (4.3)	28 (4.4)	
ȃHospital I	82 (6.4)	42 (6.4)	40 (6.3)	
ȃHospital J	87 (6.8)	42 (6.4)	45 (7.1)	
ȃHospital K	2 (0.2)	1 (0.2)	1 (0.2)	
Age (y), median (IQR)	69 (64–73)	68 (63–73)	69 (65–73)	.15
BMI (kg/m^2^), median (IQR), n = 1252	25.9 (24.2–28.3)	25.8 (24.2–28.1)	25.9 (24.3–28.4)	.34
Ciprofloxacin-resistant rectal flora, n (%)	196 (15.2)	102 (15.6)	94 (14.8)	.67
Antibiotic prophylaxis used, n (%)				
ȃCiprofloxacin	1199 (93.1)	652 (100)	548 (86.2)	NA
ȃTrimethoprim/sulfamethoxazole	22 (1.7)	…	22 (3.5)	
ȃFosfomycin	13 (1.0)	…	13 (2.0)	
ȃPivmecillinam + amoxicillin/clavulanic acid	9 (0.7)	…	9 (1.4)	
ȃCiprofloxacin + trimethoprim/sulfamethoxazole	20 (1.6)	…	20 (3.1)	
ȃCiprofloxacin + fosfomycin	15 (1.2)	…	14 (2.2)	
ȃCiprofloxacin + pivmecillinam + amoxicillin/clavulanic acid	7 (0.5)	…	7 (1.1)	
ȃCiprofloxacin + ceftazidime	2 (0.2)	…	2 (0.3)	
ȃCeftazidime	1 (0.1)	…	1 (0.2)	
Type of prostate biopsy, n (%)				
ȃRandom TRUSPB	449 (34.9)	221 (33.9)	228 (35.8)	.78
ȃTRUSPB with additional targeted (cognitive) MRI-TRUS fusion-guided PB	746 (57.9)	380 (58.3)	366 (57.5)	
ȃTargeted MRI-TRUS fusion-guided PB only	70 (5.4)	38 (5.8)	32 (5.0)	
ȃTargeted in-bore MRI-guided PB only	23 (1.8)	13 (2.0)	10 (1.6)	
Number of biopsy cores, median (IQR)	12 (10–13)	12 (10–14)	12 (10–13)	.79
Histopathology positive for malignancy, n (%)	900 (69.9)	449 (68.9)	451 (70.9)	.42
Age-adjusted Charlson Comorbidity Index, median (IQR)	3 (2–4)	3 (2–4)	3 (2–4)	.21
History of diabetes mellitus, n (%)	120 (9.3)	49 (7.5)	71 (11.2)	<.05
Drug-induced immunosuppression, n (%)	31 (2.4)	15 (2.3)	16 (2.5)	.80
Previous prostate biopsy, n (%), n = 1283	299 (23.3)	156 (24.0)	143 (22.6)	.75
Current smoker, n (%), n = 1278	131 (10.3)	66 (10.2)	65 (10.4)	.42
Indwelling catheter in situ or intermittent catheterization, n (%), n = 1266	34 (2.7)	22 (3.4)	12 (1.9)	.10
Invasive (diagnostic) procedure of the urinary tract ≤30 d before PB, n (%) n = 1264	24 (1.9)	16 (2.5)	8 (1.3)	.13
International Prostate Symptom Score, median (IQR), n = 1253	9 (5–16)	10 (5–16)	9 (5–16)	.62
Prostate-specific antigen (µg/L), median (Q1-Q3)	8.0 (5.6–11.6)	7.5 (5.5–11.3)	8.3 (5.6–12.0)	.66
Prostate volume (cm^3^), median (IQR), n = 1204	51.0 (38.0–69.5)	50.7 (38.3–69.0)	51.0 (38.0–69.5)	.70

Abbreviations: BMI, body mass index; IQR, interquartile range; MRI, magnetic resonance imaging; PB, prostate biopsy; Q, quarter; TRUSPB, transrectal ultrasound-guided prostate biopsy.

### Primary Outcome

**Figure 3.**
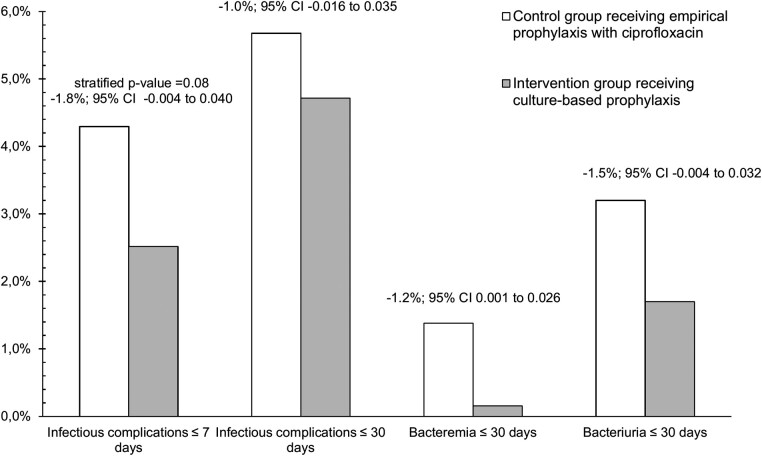
Results of the primary and secondary outcome measures per group. Point estimates of effects with 95% confidence intervals for the difference between the control and intervention groups are depicted.

**Figure 4.**
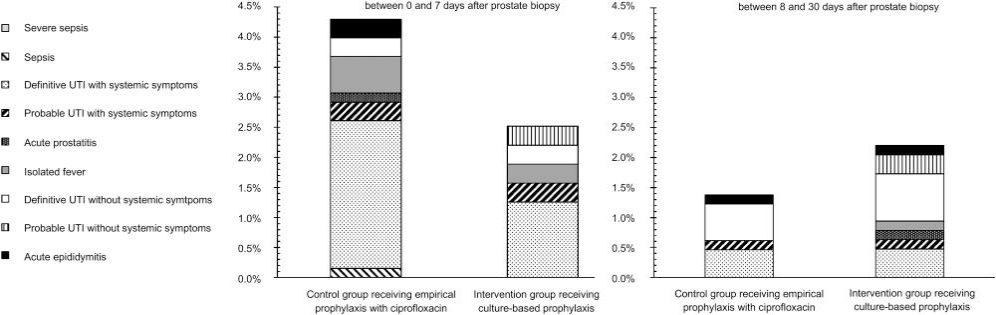
Type of early and late postbiopsy infections per group.

Infection rates within 7 days postbiopsy were 4.3% (n = 28) using empirical prophylaxis (CG) and 2.5% (n = 16) using culture-based prophylaxis (IG) (stratified *P* value = .08; reduction, −1.8%; 95% CI: −.004 to .040) (Figure 3). Most patients had systemic symptoms (CG, 85.7%; IG, 75.0%) (Figure 4). Infections occurred after a median of 2 days (IQR, 1–3). The number of patients needed to screen to prevent 1 early postbiopsy infection amounted 56 (95% CI: 25–∞). In retrospect, 53.6% of the patients with an early postbiopsy infection in the CG had prophylaxis-resistant bacteria in their prebiopsy rectal culture. In the CG, infections occurred in 2.4% of the patients with ciprofloxacin-sensitive rectal flora and 14.7% of the patients with ciprofloxacin-resistant rectal flora (6.2-fold higher risk) (difference, −12.3%; 95% CI: .061–.211), compared with 2.6% and 2.1% in the IG, respectively.

In the IG, patients with an early postbiopsy infection (n = 16) used the following prophylaxis: ciprofloxacin (88%); fosfomycin (6%), and pivmecillinam combined with amoxicillin/clavulanic acid (6%). None of these patients had prophylaxis-resistant gram-negative bacteria (GNB) in the culture of the (second) rectal swab collected immediately before PB (not used for choosing antibiotic prophylaxis).

### Post Hoc Analysis for the Primary Outcome

Data were fitted into a binary logistic regression model to adjust for covariates (ie, potential confounders) that were imbalanced after randomization (diabetes mellitus, indwelling catheter, or intermittent catheterization). Similar to the original analysis, stratification for hospital was applied. Post hoc logistic regression showed a significantly reduced risk of infection within 7 days after biopsy with the use of culture-based prophylaxis (odds ratio, 0.52; 95% CI: .267–.993) (for comparison: unadjusted odds ratio, 0.58; 95% CI: .308–1.101).

### Secondary Outcomes: Infectious Complications Within 30 Days After Biopsy

With regard to late infections, occurring between 8 and 30 days postbiopsy, no effect of culture-based prophylaxis was seen (Figure 3). Late infections occurred in 1.4% (n = 9) of the patients in the CG (median, 12 days; IQR, 10–17) (prebiopsy prophylaxis-resistant rectal flora, 44.4%) and in 2.2% (n = 14) of the patients in the IG (median, 16 days; IQR, 13–21). Less than one-half of the patients had systemic symptoms (CG, 44.4%; IG, 42.9%) (Figure 4). In the CG, late infections occurred in 0.9% of the patients with prophylaxis-sensitive rectal flora and 3.9% of the patients with prophylaxis-resistant rectal flora (4.2-fold higher risk) (difference, −3.0%; 95% CI: .0004–.094).

### Secondary Outcome: Microbiological Outcome Measures

Except for 1 (CG), all bacteremia cases occurred within the first week after biopsy (pathogens: 80% *E coli* and 20% *Klebsiella pneumoniae*). In the CG, 28.6% of the early postbiopsy infections were accompanied by bacteremia, which was 6.3% in the IG (Figure 3).

Urine cultures were taken in 79.1% of the patients with a postbiopsy infection. In 32 of these urine cultures, a pathogen was isolated (60.4%). All early urine culture-proven infections were caused by *E coli*. In late infections, in the CG, GNB (n = 3; 50%) and Enterococcus species (n = 3; 50%) were isolated from urine cultures, and in the IG *E coli* (n = 4; 66.7%), *Aerococcus* species (n = 1; 16.7%) and *Staphylococcus saprophyticus* (n = 1; 16.7%) (the latter 2 in patients performing intermittent catheterization). Within 30 days after biopsy, 3.2% (CG) and 1.7% (IG) of the patients had an urine culture–proven infection (Figure 3).

Combining all blood- and/or urine culture–proven infections, 68.4% of the early postbiopsy culture-proven infections in the CG were caused by an intermediate (n = 4) or resistant (n = 9) GNB to ciprofloxacin. The prebiopsy rectal culture showed ciprofloxacin-resistant rectal flora in 84.6% of these patients. In the IG, 60.0% of the early culture-proven infections were caused by intermediate (n = 1) or resistant GNB (n = 2) to ciprofloxacin. These patients received ciprofloxacin prophylaxis because no ciprofloxacin-resistant bacteria were isolated in the prebiopsy rectal culture. Additional microbiological data are available in Supplementary File 4.

### Exploratory Outcomes

Hospitalization rates within 30 days after biopsy amounted 2.6% (n = 17) (CG) and 1.3% (n = 8) (IG) (reduction, −1.3%; 95% CI: −.003 to .031). Patients were hospitalized for a median of 4 days (IQR, 3–5), mostly within the first week after biopsy (92.0%). No patients were admitted to the intensive care unit. One patient died during the follow-up period (unrelated to postbiopsy infection). Within 7 days postbiopsy, a total of 62 antibiotics were prescribed (441 treatment days) in the CG compared with 30 antibiotics (233 treatment days) in the IG. Within 30 days after biopsy, a total of 74 antibiotics (552 treatment days) were prescribed in the CG compared with 45 antibiotics (371 treatment days) in the IG. In both groups, the median number of antibiotics and total treatment duration per patient were comparable, namely 2 antibiotics for 14 days. No remarkable differences in adverse events between the different prophylactic agents were observed.

### Prebiopsy Rectal Cultures

In the prebiopsy rectal cultures, growth of relevant bacteria was observed on 196 agars containing ciprofloxacin (n = 1288; 15.2%), interpreted as ciprofloxacin resistance. Growth rates on the agar with ciprofloxacin varied considerably between hospitals (also within the same geographic region) from 11.9% to 26.3%. Zooming in on the 636 patients in the IG, swabs were collected a median of 22 days (IQR, 15–32) before PB; ciprofloxacin resistance was found in 14.8%. Additional microbiological data are available in Supplementary File 4.

## DISCUSSION

This multicenter trial supports the use of rectal culture-based prophylaxis to reduce early infectious complications after transrectal PB. Even though the absolute reduction of infections is small because of the small event rate, the effect is clinically relevant because PBs are performed frequently, leading to >40 000 postbiopsy infections in the United States yearly. Importantly, culture-based prophylaxis also reduced severe infections (ie, bacteremia).

Our study is the first RCT with sufficient power on this topic, adding substantial high-quality evidence. The multicenter trial design increases the robustness of our data. Considerable variation, however, exists in the number of included patients per hospital (Table 2). To account for any cluster effect, stratified randomization and analysis for hospital was performed. Given the low event rate of our primary outcome, interhospital differences in effect can only be reliably estimated for those with the highest inclusion rates (hospital B and G) (Supplementary File 5).

Ninety-one patients (5.9%) dropped out after randomization and before PB, 56 (3.6%) of which because PB was cancelled for various reasons. This dropout was largely unforeseen and related to the start of the coronavirus disease 2019 pandemic in March 2020. Importantly, reasons and patient numbers for dropout after randomization were comparable for both groups. Because randomization results were not blinded, some selection bias could have occurred. Two patients in the CG dropped out because of a high risk of postbiopsy infection. For 12 patients in the CG (1.9%), the exact reason for canceling PB or withdrawing informed consent could not be ascertained. If this had any impact on our results, it would have underestimated our results. Two patients in the IG withdrew informed consent because of the prescribed prophylaxis regimen.

Our results are in line with 3 meta-analyses [10–12]. Additionally, 2 large retrospective studies reported that culture-based prophylaxis and empirical prophylaxis resulted in similar sepsis and/or hospitalization rates within 30 days after PB. Also in our study, late infections, occurring after the first week after biopsy, were not reduced with culture-based prophylaxis. Based on etiology, late infections are likely not always procedure-related, which is supported by the weakening association of postbiopsy infection with prophylaxis-resistant rectal flora after the first week after biopsy. Additionally, the large majority of infections with systemic symptoms (78.3%) were observed within the first week. In concordance, 92.0% of all infection-related hospital admissions and 90.0% of all bacteremia cases were in this period.

When interpreting our results, some factors must be taken into account. Because of the nature of our (phenotypic) culture technique, the susceptibility of Gram-positive bacteria, particularly *Enterococcus* species—in previous studies causing 9.0% of all postbiopsy UTIs and 1.3% of all cases of bacteremia—was not determined [18–20]. In our study, 3 UTIs were caused by *Enterococcus* species. These Gram-positive bacteria are not covered with routine ciprofloxacin prophylaxis before PB.

In general, infection rates should be interpreted in the context of the level of ciprofloxacin resistance, which is subject to geographical differences. Given the relatively low resistance for ciprofloxacin in the Netherlands (15.2%), our study is not necessarily representative for geographic regions with higher rates of resistance where the impact of culture-based prophylaxis will likely be higher.

Still, 2.5% of patients developed an early postbiopsy infection despite culture-based prophylaxis. Therefore, alternative strategies to reduce postbiopsy infections needs consideration as well (ie, transperineal PB for which previous studies reported comparable postbiopsy infection rates as our study and antibiotic prophylaxis might be omitted) [21–23]. Other alternatives are reducing biopsy core number in transrectal PB or prebiopsy rectal cleansing with povidone-iodine, which is easy to implement in daily practice [21, 24–27]. Rectal cleansing was not performed in our study. Therefore, possibly, a relatively high rectal bacterial load contributed to the development of infections in the IG.

The choice and uptake of any strategy does not depend solely on the effectiveness. In our setting, the additional costs of culture-based prophylaxis were estimated to be €79. The efficiency will be higher in geographic regions with higher rates of ciprofloxacin resistance because of an increased event rate, which is generally the case outside The Netherlands [28, 29]. Furthermore, in case of transperineal PB, more costs are involved as well as the procedure requires training of urologists and costly new biopsy devices.

Several aspects about the culture-based prophylaxis strategy need to be considered. First, the optimal timing of swab collection must be established. Liss et al showed that screening approximately 2 weeks and immediately before PB provided concordant results in 93% of all patients [30]. As in our study, the timing of screening varied considerably (Supplementary File 4). A cutoff point after which screening is insufficiently recent to guide prophylaxis is lacking.

Second, recently, in Europe, attention has been paid to the adverse events of FQ [31]. Consequently, the European guidelines on prostate cancer state to avoid FQ prophylaxis in transrectal PB [32]. The European Medicine Agency, however, did not restrict the use of FQ for short-term prophylaxis [33]. Therefore, using FQ as prophylaxis remains subject to benefit-risk assessment. FQ are an important oral option to prevent postbiopsy infection. Based on our data, in a setting with relatively low antimicrobial resistance rates, culture-based prophylaxis with a single oral antibiotic is impossible in 30.5% when avoiding ciprofloxacin (Supplementary File 4). Intravenous and oral antibiotics are equally effective, but intravenous antibiotics are more expensive, less patient friendly, and require adjusted logistics [10].

Third, the optimal culture strategy needs to be determined taking into account local antimicrobial resistance patterns and cost efficiency. Perhaps screening with other antibiotics is more sensible in other settings.

Last, 24 hours of prophylaxis is recommendable. Prolonged prophylaxis has disadvantages (adverse events and selection of resistant bacteria). Evidence that supports long-course prophylactic regimens is lacking, except for pivmecillinam/amoxicillin/clavulanic acid [19].

## CONCLUSIONS

Our study supports using culture-based prophylaxis to reduce infectious complications after transrectal PB, especially with regard to the early, more severe infections. Despite adequate prophylaxis, patients can still develop postbiopsy infection. Therefore, other strategies need to be explored as well.

## Supplementary Data

Supplementary materials are available at *Clinical Infectious Diseases* online. Consisting of data provided by the authors to benefit the reader, the posted materials are not copyedited and are the sole responsibility of the authors, so questions or comments should be addressed to the corresponding author.

## Supplementary Material

ciac913_Supplementary_Data

## Notes

***Author contributions.*** Conception and design of the study: E. L. K., H. F. L. W., S. C. M. T., J. P. M. S., S. T., E. L. K., and D. M. S. Acquisition of data: S. C. M. T., E. L. Koldewijn, D. M. S., F. J. M. D., M. A. v. L., A. J. B., T. F. d. V., H. J. H. P. B., R. A. S., M. G. S., M. C. A. W.-B., E. d. B., W. v. d. B., and A. C. A. P. L. Analysis and interpretation of data: S. C. M. T. and S. T. Drafting the submitted article: S. C. M. T. Revision of the submitted article: E. L. Koldewijn, H. F. L. W., J. P. M. S., E. L. K., D. M. S., F. J. M. D., M. A. v. L., A. J. B., T. F. d. V., H. J. H. P. B., R. A. S., M. G. S., M. C. A. W.-B., E. d. B., W. v. d. B., A. C. A. P. L., and M. G. S. The corresponding author attests that all listed authors meet authorship criteria.

***Acknowledgments*.** All local staff is gratefully acknowledged for their efforts and important contribution to our study.

***Financial support.*** This work was supported by The Netherlands Organisation for Health Research and Development (ZonMw) (grant number 541001009). The funder had no role in the study design, in the collection, analysis, and interpretation of data, in the writing of the report, and the decision to submit the article for publication. There was no commercial support for this study.

***Data sharing*.** The processed anonymized data will be made available following publication and approval by Heiman Wertheim of any formal request with a defined analysis plan. Data may only be used in the context of collaboration and after agreements are made about publication and authorship. Data may only be used for research questions for which our study group is not working on. For more information on this process, or to make a request, please send an email to heiman.wertheim@radboudumc.nl. The final clinical study protocol as well as a summary of amendments made to the original protocol is available in Supplementary File 1.

***Transparency*.** The guarantors affirm that the manuscript is an honest, accurate, and transparent account of the study being reported; no important aspects of the study have been omitted, and any discrepancies from the study as originally planned (and registered) have been explained.
