# Supplementary material for: Rectal Culture-Based Versus Empirical Antibiotic Prophylaxis to Prevent Infectious Complications in Men Undergoing Transrectal Prostate Biopsy: A Randomized, Nonblinded Multicenter Trial
Source: Clin Infect Dis. 2022 Nov 24;76(7):1188–96. doi: 10.1093/cid/ciac913 (PMC10069853; doi:10.1093/cid/ciac913)
Supplement: ciac913_Supplementary_Data [file ciac913_supplementary_data.docx]

**Rectal culture-based versus empirical antibiotic prophylaxis to prevent infectious complications in men undergoing transrectal prostate biopsy:**

**a randomised, non-blinded multicenter trial**

**Supplementary Appendix**

**Table of contents**

Supplementary File I: Final study protocol 3-43

Supplementary File II: Rectal Culture Protocol 44

Supplementary File III: Patient Questionnaires 46-60

Supplementary File IV: Microbiological outcomes 61-67

Supplementary File V: Differences in effect between hospitals 68

**Supplementary I: Final study protocol, version 11.0, August 4, 2020**

**PRO-SWAP**

The effect of rectal swab culture-guided antimicrobial prophylaxis in men undergoing prostate biopsy on infectious complications and cost of care: A randomized controlled trial in the Netherlands.

Principal investigators Dr. E. Kolwijck, Dept. of Medical Microbiology

Radboud University Medical Center, huispost 777

P.O. box 9101, 6500 HB Nijmegen, the Netherlands

Tel: +31-(0)24 361 4356 - Fax: + 31-(0)24 363 5153

Email: eva.kolwijck@radboudumc.nl

Dr. J.P.M. Sedelaar, Dept.of Urology

Radboud University Medical Center, huispost 610

P.O. box 9101, 6500 HB Nijmegen, the Netherlands

Tel: +31-(0)24 361 3735

Email: michiel.sedelaar@radboudumc.nl

Dr. D.M. Somford, Dept. Of Urology

Canisius Wilhelmina Hospital

Weg door Jonkerbos 100

6532 SZ Nijmegen, the Netherlands

Tel: +31-(0)24 365 8832

Email: r.somford@cwz.nl

Dr. E.L. Koldewijn, Dept. of Urology

Catharina Hospital

Michelangelolaan 2

5623 EJ Eindhoven, the Netherlands

Tel: +31-(0)40 239 7040

Email: evert.koldewijn@catharinaziekenhuis.nl

Dr. T. F. de Vocht, Dept. of Urology

Bernhoven Hospital

Nistelrodeseweg 10

5406 PT Uden, the Netherlands

E-mail: t.devocht@bernhoven.nl

Dr. R.A. Schipper

Jeroen Bosch Hospital

Henri Dunantstraat 1

5223 GZ ’s-Hertogenbosch, the Netherlands

E-mail: r.schipper@jbz.nl

Dr. M.G. Scheffens

Isala Hospital

Dr. van Heesweg 2

8025 AB Zwolle, the Netherlands

E-mail: m.steffens@isala.nl

Dr. M.R. van Balken

Rijnstate Hospital

Wagnerlaan 55

6815 AD Arnhem

E-mail: mvanbalken@rijnstate.nl

Dr. F.J.M. Delaere

Zuyderland

Henri Dunantstraat 5

6419 PC Heerlen

Email: f.delaere@zuyderland.nl

Dr. A.J. Breeuwsma

Elisabeth Tweesteden Ziekenhuis

Dr. Deelenlaan 5

5042 AD Tilburg

Email: a.j.breeuwsma@etz.nl

Dr. D.K.E. van der Schoot

Amphia Hospital

Molengracht 21

4818 CK Breda, the Netherlands

Email: dvdschoot@Amphia.nl

Dr. M.A. van Leeuwen

Bravis Hospital

Boerhaavelaan 25

4708 AE Roosendaal, the Netherlands

Email: s.vanleeuwen@bravis.nl

Study coordinator Drs. S.C.M. Tops - van Kuppevelt, Dept. of Medical Microbiology

Radboud University Medical Center, huispost 777

P.O. box 9101, 6500 HB Nijmegen, the Netherlands

Tel: +31-(0)6 27 15 41 17 / +31-(0)24 361 4356

E-mail: sofie.tops@radboudumc.nl

Projectmembers

Dr. H.F.L. Wertheim Radboudumc Professor of medical microbiology, head of

clinical microbiology department

Dr. E.M.M. Adang Radboudumc Associate professor health technology assessment

and cost-effectiveness analyses in medical research

Dr. R.J.M. Brüggemann Radboudumc Senior hospital pharmacist, scientist

Drs. R.T.M. van Duren Mediaproducts B.V. Director

Dr. A.M.P. Huis Radboudumc Researcher at IQ healthcare

Prof dr. M.E.J.L. Hulscher Radboudumc Professor Quality of care for infectious and

inflammatory diseases, senior researcher

at IQ healthcare

Dr. J. Schouten Radboudumc Researcher at IQ Healthcare

Dr. D. Masman Radboudumc Director of valorization

Dr. M.C.A. Wegdam-Blans PAMM Clinical microbiologist

Dr. P.M. Schneeberger Jeroen Bosch Hospital Clinical microbiologist

Dr. R.W. Bosboom Rijnstate Hospital Clinical microbiologist

Dr. E. De Brauwer Zuyderland Clinical microbiologist

Dr. J. Murk Elisabeth Tweesteden Clinical microbiologist

Dr. W. Van den Bijllaardt Microvida Clinical microbiologist

**CLINICAL TRIAL PROTOCOL**

|  |  |
| --- | --- |
| Trial Title | The effect of rectal swab culture-guided antimicrobial prophylaxis in men undergoing prostate biopsy on infectious complications and cost of care: A randomized controlled trial in the Netherlands. |
| Short title | PRO-SWAP study |
| Start date | April 2018 |
| Duration of study | 3.5 years |
| Protocol ID | pro-SWAP104622 |
| ABR number | NL63566.091.17 |
| EudraCT number | 2017-002938-23 |
| Sponsor | Radboud University Medical Center  P.O. box 9101, 6500 HB Nijmegen, The Netherlands |
| Subsiding party | ZonMw, project number 541001009  Programme: antimicrobial resistance (ABR) |
| Participating hospitals: | Amphia Hospital, Breda  Bernhoven hospital, Uden  Bravis Hospital, Roosendaal and Bergen op Zoom  Canisius Wilhelmina hospital, Nijmegen  Catharina hospital, Eindhoven  Elisabeth Tweesteden Ziekenhuis, Tilburg  Isala hospital, Zwolle  Jeroen Bosch hospital, ‘s-Hertogenbosch  Radboudumc, Nijmegen  Rijnstate hospital, Arnhem  Zuyderland, Heerlen en Sittard-Geleen |
| Independent expert | Dr. J.J. Hoogerwerf, Dept. of Infectious diseases  Radboud University Medical Center  Huispost 463, P.O. box 9101, 6500 HB Nijmegen, the Netherlands  Tel: +31-(0)24 361 0175 |
| Laboratory sites | Medical Microbiology laboratory Radboud University Medical Center  Huispost 777, P.O. box 9101, 6500 HB Nijmegen, the Netherlands  Tel: +31-(0)24 361 4356  PAMM, Medical Microbiology laboratory  P.O. box 2, 5500 AA Veldhoven, the Netherlands  Tel: +31-(0)40 888 8100  Medical Microbiology laboratory Jeroen Bosch hospital  P.O. box 90153, 5200 ME ‘s-Hertogenbosch, the Netherlands  Tel: +31-(0)73 553 2000  Medical Microbiology laboratory Rijnstate hospital  P.O. box 9025, 6800 EG Arnhem, the Netherlands  Tel: +31-(0)88 005 5455  Medical Microbiology laboratory Tilburg (LMMI)  P.O. box 747, 5000 AS Tilburg, the Netherlands  Tel: +31-(0)13 221 2650  Medical Microbiology laboratory Amphia (Microvida)  Molengracht 21, 4818 CK Breda, the Netherlands  Tel: +31-(0)76 595 3015  Medical Microbiology laboratory Zuyderland  P.O. box 5500, 6130 MB Sittard, the Netherlands  Tel: +31-(0)45 576 7803 |
| Pharmacy | Pharmacy Radboudumc  Huispost 459, P.O. box 9101, 6500 HB Nijmegen, the Netherlands  Tel: +31-(0)24 361 9191  Pharmacy Catharina Hospital  P.O. Box 1350, 5602 ZA Eindhoven, the Netherlands  Tel: +31-(0)40 239 9100  Pharmacy Canisius Wilhelmina Hospital  P.O. Box 9015, 6500 GS Nijmegen, the Netherlands  Tel: +31-(0)24 365 7600  ZANOB  P.O. Box 3406, 5203 DK ’s-Hertogenbosch, the Netherlands  Tel: +31-(0)73 553 5950  Pharmacy Isala  Dr. Van Heesweg 2, 8025 AB Zwolle, the Netherlands  Tel: +31-(0)38 424 5474  Pharmacy Rijnstate  P.O. Box 9555, 6800 TA Arnhem, the Netherlands  Tel: +31-(0)88 005 6319  Pharmacy Zuyderland  Postbus 5500  6130 MB Sittard-Geleen  Pharmacy Tilburg  Dr. Deelenlaan 5  5042 AD Tilburg  Pharmacy Breda  Molengracht 21  4818 CK Breda |

**PROTOCOL SIGNATURE SHEET**

I have read the protocol, including all appendices, and I agree that it contains all necessary details for me and my staff to conduct this trial as described. I will conduct this trial as outlined herein and will make a reasonable effort to complete the trial within the time designated.

The following investigators have read and agreed with the protocol, version 8 d.d. 12-12-2017.
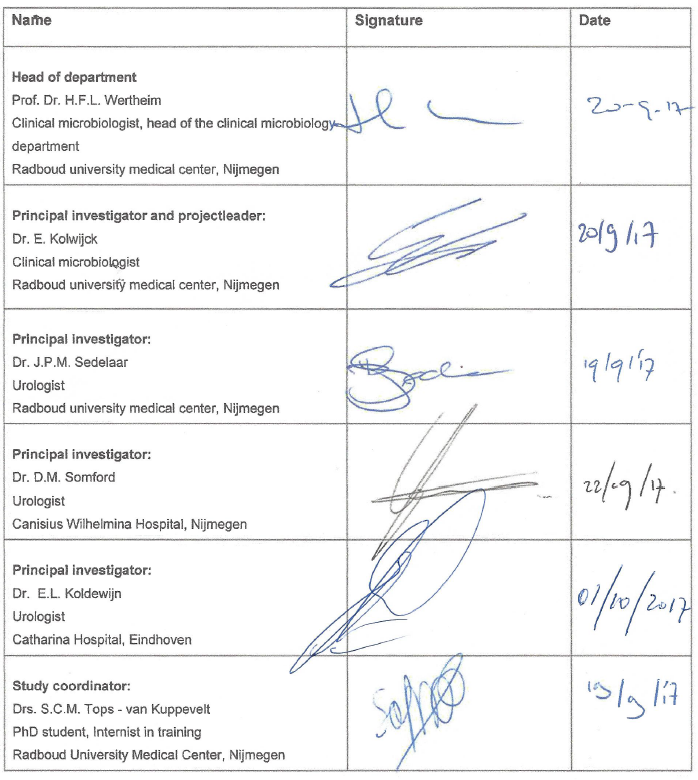


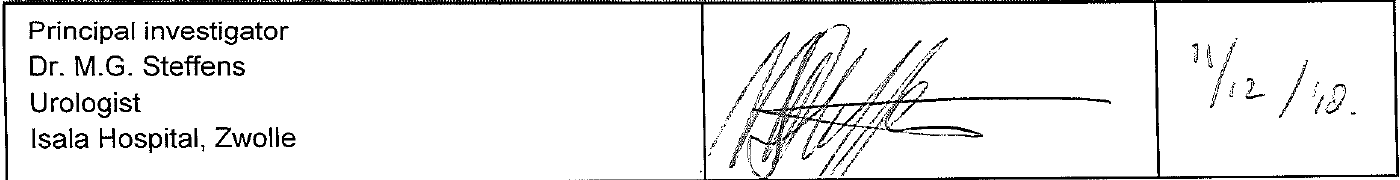


----------------------------------------------------------------------------------------------------------------------------------------

The following investigators have read and agreed with the protocol, version 11 d.d. 04-08-2020.


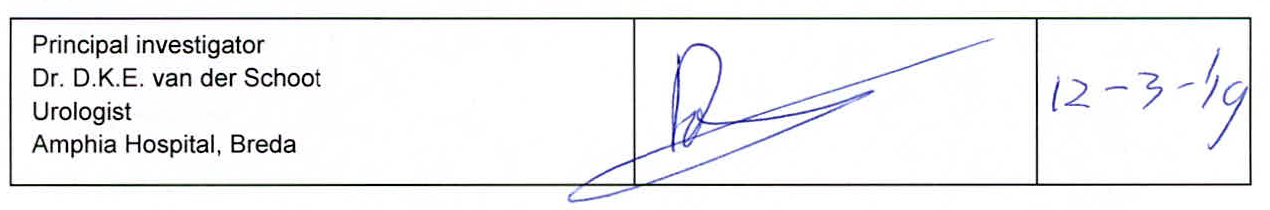

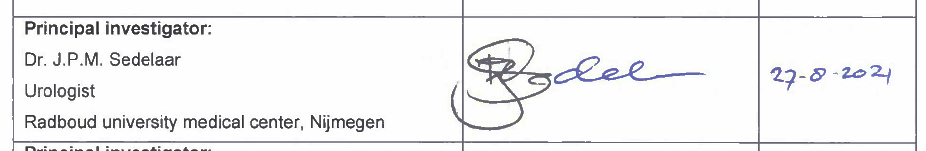

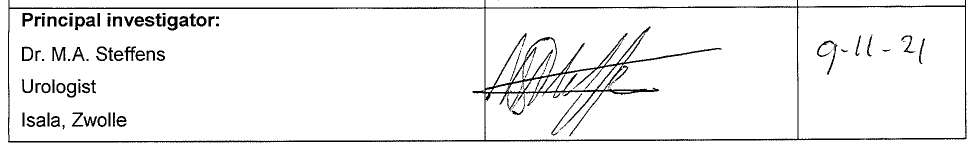

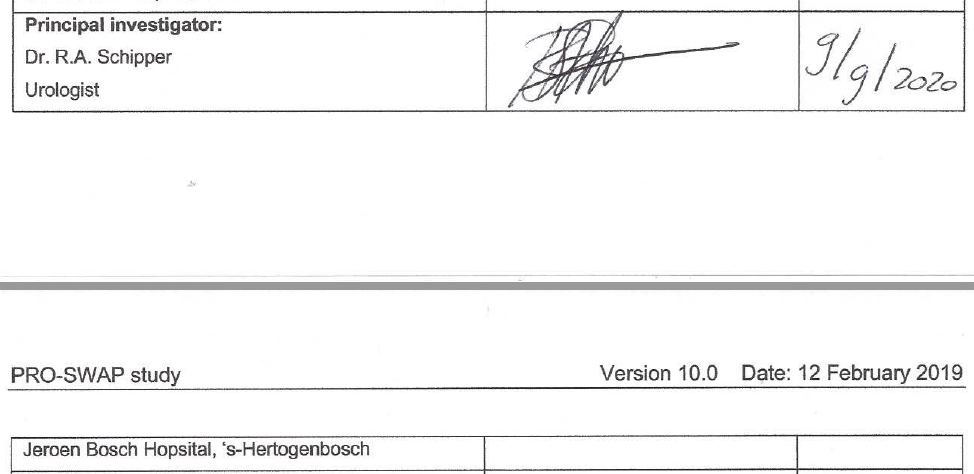

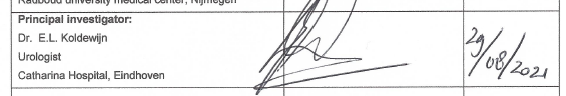


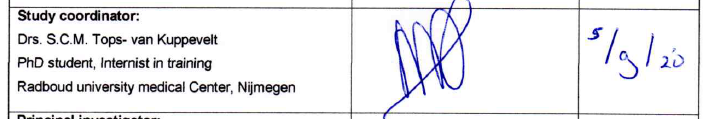


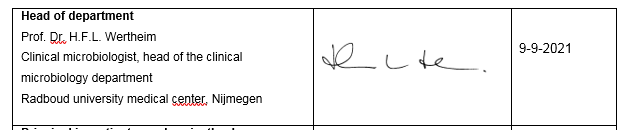

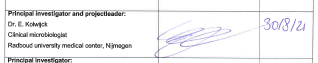


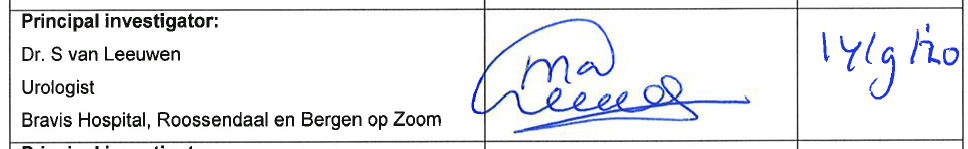

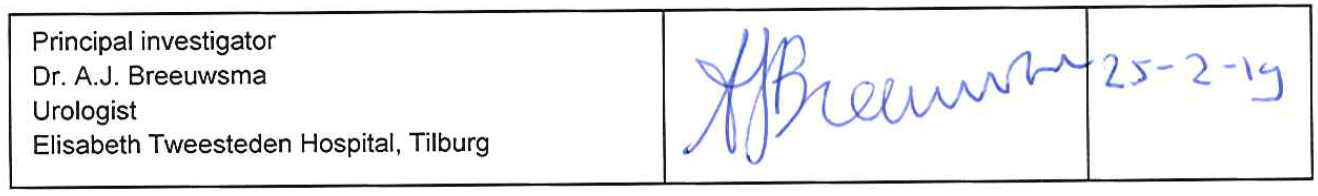

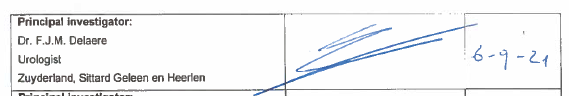

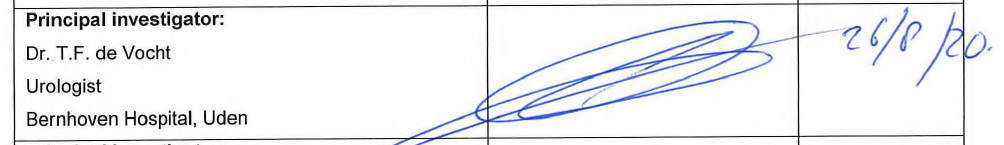


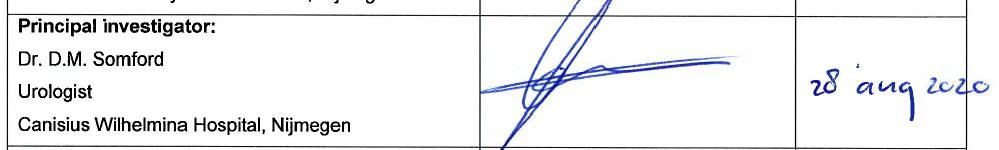


I will provide all trial personnel under my supervision copies of the protocol. I will discuss this material with them to ensure that they are fully informed about the drugs and the trail.

**SUMMARY**

**Study design:** Prospective non-blinded randomized controlled trial (intervention study).

**Objectives:**

- To assess the effectiveness of rectal culture-guided antimicrobial prophylaxis for transrectal prostate biopsy (random ultrasound-guided, targeted MRI-guided, targeted MRI-ultrasound fusion guided) on infectious complications.
- To compare the cost-effectiveness of empirical antimicrobial prophylaxis (standard of care) to the rectal culture-guided antimicrobial prophylaxis strategy for transrectal prostate biopsy.
- To assess experiences of patients and professionals regarding barriers and facilitators (determinants) that influence the performance of the culture-guided approach in daily clinical practice.

**Rationale:** Transrectal ultrasound guided prostate biopsy (TRUS-PB) is a well-established procedure to obtain tissue for the histological diagnosis of prostate cancer. During TRUS-PB, a spring-loaded device is used to collect multiple core biopsies, sampling tissue systematically from both sides of the gland. A variety of infectious complications may occur following transrectal prostate biopsy, ranging from asymptomatic bacteriuria or urinary tract infection (UTI) to prostatitis, bacteremia, and severe sepsis. Although no studies have clearly defined the pathophysiology of TRUS-PB related infectious complications, the cause appears to be direct inoculation of bacteria from the rectal mucosa by the biopsy needle into the prostate, blood vessels, or urinary tract.

Several classes of antibiotics are proven effective for prophylaxis during TRUS-PB, reducing infectious complications to less than 1% in case of susceptible rectal flora. Ciprofloxacin has been best studied and is recommended as first choice prophylaxis in urology guidelines, although no clear recommendations are made on the duration of prophylaxis. In the Netherlands, therefore, various prophylactic ciprofloxacin schedules are used, of which 2 to 3 day regimens are most common. Prolonged duration of prophylaxis in TRUS-PB is not proven to be more effective than a 1-day regimen, but it is more likely to select more fluoroquinolone (FQ) resistance.

Due to increasing FQ resistance in gram negative bacilli (GNB) (currently more than 20% in *E. coli*), a significant increase up to 6% in infectious complications after TRUS-PB procedures was recently noticed. Antibiotic treatment of these infections and hospitalization may account for increased health care associated costs and will contribute to the further development of antibiotic resistance.

Directed antibiotic prophylaxis based on resistance data of individual rectal cultures may be an effective strategy to overcome the problem of increasing FQ resistance and reduce infectious complications after transrectal prostate biopsy. Last years, a few studies have been published on targeted antibiotic prophylaxis in TRUS-PB. These studies already showed promising results but had retrospective designs, did not use control groups, used unclear screenings techniques or were underpowered. In addition, expensive and time-consuming bacterial culture methods were performed. Moreover, the majority of these studies used intravenous antibiotics in case of FQ resistance. Oral alternatives to FQ, if available, are preferred over intravenous prophylaxis, because they are cheaper and more comfortable for the patient.

In the Radboudumc, MRI-guided or MRI-ultrasound fusion guided prostate biopsies are performed in almost all patients instead of TRUS-PB. MRI of the prostate is capable of detecting clinically relevant prostate cancer. Therefore patients with a normal MRI of the prostate may not need to undergo transrectal prostate biopsy. Moreover, MRI-guided or MRI-ultrasound fusion guided lesion targeting, allows urologist to progress from blind, systematic biopsies with at least 12 core samples to more accurate targeted biopsies with only 2 till 4 core samples. Because less core samples are taken with these techniques, the amount of infectious complications after MRI-guided or MRI-ultrasound fusion guided prostate biopsies might be reduced compared to TRUS-PB. However, there is no literature available on this subject.

This study aims to assess the effectiveness and cost-effectiveness of rectal culture-guided antimicrobial prophylaxis to reduce infectious complications after transrectal prostate biopsy. Also, duration of antibiotic prophylaxis will be minimized to 24 hours, thereby controlling further development of resistant bacteria. Moreover, we will be able to compare the difference of infectious complications after random transrectal ultrasound-guided, targeted MRI and MRI-ultrasound fusion guided prostate biopsy. Lastly, we will assess experiences of patients and professionals regarding barriers and facilitators (determinants) that influence the performance of the culture-guided approach in daily clinical practice. Such information is important to take into account when assessing the (cost) effectiveness of rectum culture-directed antibiotic prophylaxis strategy and to facilitate its future implementation into daily practice.

**Hypothesis:** The targeted prophylaxis group will have a 2.2% reduction of post-biopsy infectious complications compared to the control group (overall estimated at respectively 1.0% and 3.2%).

**Study population:** Men suspect of prostate cancer undergoing transrectal prostate biopsy as part of standard of care, in one of the participating study centers (see page 4), will be included after written informed consent is obtained. Patients will be randomized into two groups of 666 patients each:

1. Control group receiving routine empirical prophylaxis with oral ciprofloxacin (500 mg orally 2 hours before and 12 hours after the procedure)
2. Intervention group receiving rectal culture-guided oral antibiotic prophylaxis. Men whose rectal swabs do not show ciprofloxacin-resistant bacteria will receive ciprofloxacin prophylaxis (see 1), and men whose swabs do show ciprofloxacin-resistant bacteria will receive alternative oral antibiotics based on the culture results (in the following order):

- trimethoprim/sulfamethoxazole (SXT) 960 mg orally 2 hours before the procedure and again 12 hours later, or
- fosfomycin 3 g orally 2 hours before the procedure, or
- pivmecillinam/amoxicillin/clavulanic acid respectively 400 mg and 500/125 mg 2 hours before biopsy, followed by 2 days with three divided doses each day after biopsy.

Exclusion criteria:

- Inability to receive ciprofloxacin prophylaxis for any reason:
  - documented history of sensitivity to medicinal products or excipients similar to those found in the antibiotics used as prophylaxis
  - relevant history or presence of cardiovascular disorders (specific relevant Qtc time prolongation)
  - relevant drug interaction
  - an increased risk of fluoroquinolone-induced tendinitis or tendon rupture (specifically patients with decreased renal function defined as MDRD <30 ml/min/1.73m^2^, patients with solid organ transplants, concomitant use of corticosteroids or a history of fluoroquinolone-induced tendinopathy).
  - an increased risk of aortic anneurysm or dissection (specifically patients with a connective tissue disease (e.g. Marfan syndrome, Ehlers-Danlos syndrome, large vessel arteritis (Tayasu, gaint cell), Behçet disease), patients with a history of aortic aneurysm or dissection or patients with a family history (siblings) of aortic aneurysm or dissection).
- Inability to receive either co-trimoxazole, fosfomycin and pivmecillinam/amoxicillin/clavulanic acid prophylaxis for any reason (e.g. documented history of sensitivity to medicinal products or excipients similar to those found in the antibiotic used as prophylaxis, relevant drug interaction)
- Individuals with UTI or acute prostatitis within 14 days prior to transrectal prostate biopsy
- Individuals who received antibiotics within 14 days prior to transrectal prostate biopsy
- Individuals who fail to send a rectum swab to the microbiology laboratory
- Individuals whose rectal swab shows no growth on a Colombia blood agar (CBP) (growth control)
- Repeat biopsy within 7 days

**Main study parameters/endpoints:**

Primary endpoints:

1. Any registered clinical infectious complication (UTI, pyelonephritis, sepsis, fever, acute prostatitis, acute epididymitis) within 7 days after transrectal prostate biopsy.

Secondary endpoints:

1. Cost of care 30 days after transrectal prostate biopsy to determine and compare overall costs of care among the intervention- and the control group.
2. Positive microbiological urine or blood culture results within 7 and 30 days after transrectal prostate biopsy.
3. Clinical infectious complications (as for primary endpoint) within 30 days after transrectal prostate biopsy.

Exploratory:

1. Hospitalization, including ICU admission and length of admission, within 30 days after transrectal prostate biopsy.
2. Overall mortality within 30 days after transrectal prostate biopsy.
3. Side effects and toxicity of used antibiotics within 30 days after transrectal prostate biopsy.
4. Prevalence of ciprofloxacin resistant GNB in local rectal flora, assessed through microbiological rectal swab cultures.
5. Overall antibiotic use within 30 days after transrectal prostate biopsy.
6. The relation between antibiotic resistance in the fecal carriage and other determinants of infection after transrectal prostate biopsy.
7. Experiences of patients and healthcare professionals regarding barriers and facilitators (determinants) that influence the performance of the culture-guided approach in daily clinical practice.

**Nature and extent of the burden and risks associated with participation, benefit and group relatedness:**

Nature and extent of the burden associated with participation:

- At three moments during the study period patients have to fill in a few questionnaires:
- Baseline (approximately 7 days prior to prostate biopsy): questionnaire about demographic parameters, use of medication (including antibiotics), allergies and other parameters which might intervene with the study outcome, EQ-5D-5L (to assess health status), iMTA PCQ (to assess productivity loss).
- At approximately 7 days and 30 days after prostate biopsy: questionnaire about the study measurements, EQ-5D-5L, iMTA PCQ.
- At two moments during the study period a rectal swab will be taken (at home by self-sampling approximately 14 days prior to prostate biopsy and immediately before prostate biopsy by the urologist). Utility of a rectum swab culture is a minimally invasive method to provide information on patients' rectal flora.
- At baseline, some patients (phase 1) will be approached for an in-depth interview (approximately 5-10 patients per center, for approximately 1 hour per patient) as part of an analysis of determinants that influence the performance of the culture-guided approach in daily clinical practice. Interviews will take place at a with the patient agreed upon moment and location. Based upon these results, a questionnaire will be developed to assess barriers and facilitators experienced. Thereafter (phase 2), approximately 50-100 patients per center will be asked to fill in an extra questionnaire at baseline (time investment approximately 15 minutes).
- Similarly, after patient inclusion has ended, a study among healthcare professionals will be performed to assess barriers and facilitators that influence the performance of the culture-guided approach in daily clinical practice. Professionals involved in performing the culture-guided approach will be approached for an in-depth interview (approximately 3-5 professionals per center, for approximately 1 hour per professional). Based upon these results, a questionnaire will be developed to assess barriers and facilitators experienced among Dutch professionals.
- According to the laws and regulations associated with research with a medicinal product, the study medication must be provided from the Clinical Trial Units of the hospital pharmacies of the participating centers. Therefore, participants can not obtain the antimicrobial prophylaxis at their home pharmacy. Participants will be offered two choices: to obtain the antimicrobial prophylaxis in the days before prostate biopsy at the outpatient department Urology (consequence: extra trip to the hospital) or to be present in the hospital two hours prior to prostate biopsy and then obtain the antimicrobial prophylaxis at the outpatient department Urology (and take the antimicrobial prophylaxis immediately). No extra other (study-related) hospital visits are required.

Risks associated with participation:

The risk-classification is assessed as negligible to the study participants.

Utility of a rectum swab culture is a minimally invasive and safe method to provide information on a participant’s rectal flora. It is already routine procedure for patients, admitted to Dutch hospitals, that are at risk for carriage of multidrug resistant bacteria (HRMO screening or methicillin-resistant Staphylococcus aureus (MRSA).(39)

The alternative antibiotics which may be prescribed in case of FQ resistance are all registered in the Netherlands, used in clinical practice for the treatment of UTI and/or prostatitis and logical alternatives as antimicrobial prophylactic around prostate biopsy. We do not expect additional risks compared to the use of the standard antibiotic prophylaxis with ciprofloxacin and besides already registered adverse effects. In addition, it should be mentioned that it concerns antibiotic prophylaxis, which means that the antibiotics are used only for a short period (mostly only for up to 24 hours).

Benefit:
We expect that the intervention group (with rectal swab culture-guided antimicrobial prophylaxis) will have a lower risk of post-biopsy infectious complications compared to the control group (respectively 1.0% and 3.2%).

**LIST OF ABBREVIATIONS AND RELEVANT DEFINITIONS**

| AE | Adverse Event |
| --- | --- |
| AR | Adverse Reaction |
| CA  CBP | Competent Authority  Colombia blood agar |
| CCMO | Central Committee on Research Involving Human Subjects; in Dutch: Centrale Commissie Mensgebonden Onderzoek |
| DSMB | Data Safety Monitoring Board |
| E-CRF | Electronic Case Report Form |
| EudraCT | European drug regulatory affairs Clinical Trials |
| FQ | Fluoroquinolone |
| GCP  GLM | Good Clinical Practice  Generalized linear model |
| GNB | Gram-negative bacilli |
| HRMO | Highly resistant microorganisms |
| METC | Medical research ethics committee (MREC); in Dutch: medisch ethische toetsing commissie (METC) |
| NVU  OLS | Dutch Urology Association  Ordinary least squares |
| OPD | Outpatient department |
| Radboudumc | Radboud university medical center |
| (S)AE  SPC | (Serious) Adverse Event  Summary of Product Characteristics |
| Sponsor | The sponsor is the party that commissions the organisation or performance of the research, for example a pharmaceutical company, academic hospital, scientific organisation or investigator. A party that provides funding for a study but does not commission it is not regarded as the sponsor, but referred to as a subsidising party. |
| SUSAR | Suspected Unexpected Serious Adverse Reaction |
| SWAB | Dutch Working Party on Antibiotic Policy |
| SXT | Trimethoprim/sulfamethoxazol |
| TRUS-PB | Transrectal ultrasound-guided prostate biopsy |
| UTI | Urinary tract infection |
| WMO | Medical Research Involving Human Subjects Act (in Dutch: Wet Medisch-wetenschappelijk Onderzoek met Mensen |

**TABLE OF CONTENTS**

1. INTRODUCTION AND RATIONALE 17

1.1 Introduction 17

1.2 Rationale 18

2. OBJECTIVES 19

3. STUDY DESIGN 19

3.1 Part I: Randomized controlled trail investigating the difference in post-TRUS-PB infection rate

between patients receiving rectal culture-guided prophylaxis and patients receiving empirical

prophylaxis with ciprofloxacin................................................................................................................19-25

3.1.1 Overall trial design 19

3.1.2 Patient recruitment 20

3.1.3 Prophylaxis schedule for the intervention group 23

3.2 Part II: Cost-effectiveness analysis (CEA) of the culture-guided antibiotic prophylaxis strategy...............24

3.3 Part III: Assessment of determinants that help or hinder the performance of the culture-based

approach in daily clinical practice.................................................................................................................25

4. STUDY POPULATION …25

4.1 Population (base) 25

4.2 Inclusion criteria 25

4.3 Exclusion criteria 25

4.4 Sample size calculation 26

5. TREATMENT OF SUBJECTS …27

6. INVESTIGATIONAL PRODUCT 27

6.1 Overview 27

6.2 Identity of investigational products 27

6.3 Explanation of antibiotics choice and duration for this study 28

6.4 Summary of known and potential risks 29

6.5 Preparation and labelling of Investigational Medicinal Product 29

6.6 Drug accountability 30

6.7 Blinding 30

7. METHODS 30

7.1 Study parameters/endpoints 30

7.1.1 Main study parameter/endpoint 30

7.1.2 Secondary study parameters/endpoints 30

7.1.3 Exploratory 30

7.1.4 Other study parameters 30

7.2 Analysis of determinants among healthcare professionals 33

7.3 Randomisation, blinding and treatment allocation 33

7.4 Study procedures 33

7.4.1 Rectal swabs and screening cultures 33

7.4.2 Blood and urine cultures post biopsy 34

7.4.3 Storage of isolates 34

7.5 Withdrawal of individual subjects and follow-up of subjects withdrawn from treatment 34

7.6 Replacement of individual subjects after withdrawal 34

7.7 Premature termination of the study 34 SAFETY REPORTING 34

8.1 Temporary halt for reasons of subject safety 34

8.2 AEs, SAEs and SUSARs 34

8.2.1 Adverse events (AEs) 34

8.2.2 Serious adverse events (SAEs) 34

8.2.2 Suspected unexpected serious adverse reactions (SUSARs) 35

8.3 Annual safety report 35

8.4 Follow-up of adverse events 35

8.5 Data Safety Monitoring Board (DSMB) 35

9. STATISTICAL ANALYSIS 35

9.1 Primary study parameter(s) 35

9.2 Secondary study parameter(s) 36

9.3 Other study parameters 36

9.4 Interim analysis 36

10. ETHICAL CONSIDERATIONS 37

10.1 Regulation statement 37

10.2 Recruitment and consent 37

10.3 Benefits and risks assessment, group relatedness 37

10.4 Compensation for injury 38

11. ADMINISTRATIVE ASPECTS, MONITORING AND PUBLICATION 38

11.1 Handling and storage of data and documents 38

11.2 Monitoring and Quality Assurance 38

11.3 Amendments 38

11.4 Annual progress report 39

11.5 Temporary halt and (prematurely) end of study report 39

11.6 Public disclosure and publication policy 39

12. REFERENCES 39

13. Amendments to the protocol…………………………………………………………………………….42

# **INTRODUCTION AND RATIONALE**

1.1 Introduction

*Transrectal ultrasound-guided prostate biopsy (TRUS-PB)*

TRUS-PB is the procedure to obtain tissue for the histological diagnosis of prostate cancer, the most commonly diagnosed cancer in men.(1) In the Netherlands, TRUS-PB is performed in approximately 40.000 patients annually. Due to the transrectal approach of the procedure, gut bacteria may be inoculated directly into the prostate, bloodstream or urinary tract, which may lead to infectious complications, including acute prostatitis, urinary tract infection (UTI), fever and sepsis.

Several classes of antibiotics are proven effective for prophylaxis in TRUS-PB, reducing infectious complications to less than 1% in case of susceptible rectal flora.(2) Ciprofloxacin has been best studied and is recommended as first choice prophylaxis in urology guidelines.(3-5)

*Increase of post-TRUS-PB complications*

Recently, a worrisome increase in infectious complications post-TRUS-PB (from less than 1% to 6%) has been reported.(6-10) Reported infection rates varied between 2.5% and 5.5%.(11-17) In the Radboudumc, microbiological evidence of infection was found in 2.7% of 842 patients that underwent TRUS-PB between 2014 and 2015 (96% gram-negative bacilli (GNB), of which 80% was *E. coli*). However, this might be an underestimation of the true infectious complication rate because of the retrospective design and the microbiological endpoint used (not all infections were cultured and sent to our laboratory). Another Dutch retrospective study using fever as clinical outcome found 3% infections among 706 patients that underwent TRUS-PB in 2011.(6)

*Fluoroquinolone (FQ) resistance*

Rise in infectious complications post-TRUS-PB has been linked to the growing FQ resistance of GNB. In studies on infectious complications after TRUS-PB, *E. coli* is the predominant causative organism, accounting for more than 80% of positive cultures in multiple studies.(6, 9, 11, 12, 14-16, 18-20) Remaining causative bacteria are almost always GNB. Due to increased community use of FQ,(21) FQ resistance in *E. coli* isolates from patients in Dutch urology outpatient and inpatient departments in 2016 was 19% and 23%, respectively.(21) This is a rapid increase compared to 7% in 2000.(22) In rectum cultures of men undergoing TRUS-PB, FQ resistance rates between 13% and 21% have been reported.(11, 13, 15, 23-25)

In TRUS-PB, with the use of FQ prophylaxis, there was an average fivefold higher risk of infectious complications in the presence of FQ resistant rectal flora (7.9%, versus 1.6%).(26) Moreover, antibiotic treatment of these infections and hospitalization may account for increased health care associated costs and will contribute to further development of antibiotic resistance. The 2015 European Association of Urology guidelines acknowledge the concern for rising FQ resistance and the increased risk of complications in TRUS-PB, though no specific recommendations were made.(27)

*Targeted prophylaxis for TRUS-PB*

Targeted antimicrobial prophylaxis is a promising new strategy to reduce the amount of transrectal prostate biopsy related infectious complications.(26, 28, 29) It starts with pre-biopsy screening for FQ resistant bacteria in the rectum of patients undergoing transrectal prostate, allowing the identification of men where ciprofloxacin prophylaxis is not appropriate. Antimicrobial prophylaxis can then be tailored to each patient individually.

*MRI guided or MRI-ultrasound fusion guided prostate biopsy*

In the Radboudumc, MRI-guided or MRI-ultrasound fusion guided prostate biopsies are performed in almost all patients instead of TRUS-PB. MRI of the prostate is capable of detecting clinically relevant prostate cancer. Therefore patients with a normal MRI of the prostate may not need to undergo transrectal prostate biopsy.(30, 31) Moreover, MRI-guided or MRI-ultrasound fusion guided lesion targeting, allows urologist to progress from blind, systematic biopsies with at least 12 core samples to more accurate targeted biopsies with only 2 till 4 core samples.(32) Because less core samples are taken with these techniques, the amount of infectious complications after MRI-guided or MRI-ultrasound fusion guided prostate biopsies might be reduced compared to TRUS-PB. However, there is no literature available on this subject.

*Purpose of the study*

The purpose of this prospective non-blinded randomized intervention study is the development and implementation of an optimized, feasible and oral (rectal culture-guided) antibiotic prophylaxis strategy in men undergoing transrectal prostate biopsy (random ultrasound-guided, targeted MRI-guided or targeted MRI-ultrasound fusion guided), in order to reduce infectious complications and control antibiotic resistance.

| 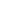 |
| --- |

1.2 Rationale

*Optimizing antimicrobial prophylaxis*

Inappropriate use of antibiotics (prolonged duration, use of broad-spectrum antibiotics, or misuse of antibiotics) can select for resistant bacteria. Guiding antibiotic choices based on susceptibility testing of rectal flora can result in minimal selection of resistance. Also, the presumed reduction of infectious complications after rectal culture-guided antimicrobial prophylaxis for TRUS-PB will diminish the use of therapeutic antibiotics and therefore prevent further development of antibiotic resistance. Culture directed prophylaxis may be an innovative strategy in the era of rising interest in personalized medicine. In addition, this strategy may be cost-effective.(33, 34)

Previous studies on targeted prophylaxis for TRUS-PB showed that this culture-based strategy was associated with significantly lower infectious complication. However, the designs of these studies were not robust and lacked power. In most studies results were compared to historical data.(13, 24, 35) Other studies had a retrospective design (23, 36) or used small numbers of patients without randomization.(25, 34) Thus, the feasibility and effectiveness of this personalized prophylaxis approach has not yet been evaluated in prospective randomized controlled trials with sufficient power. In addition to the inadequate design, the majority of these studies used intravenously administered broad-spectrum antibiotics as alternatives in case of FQ-resistance.(13, 23, 24, 34-36) Intravenous antibiotics are more expensive than oral antibiotics, less patient friendly and require an adjusted patient flow and logistics with more skilled personnel, which would be a significant barrier for the implementation of this strategy. Oral and intravenous antibiotics have been proven equally effective for TRUS-PB.(2) Currently, two randomized controlled trials on culture-guided prophylaxis for TRUS-PB (NCT01659866, NCT02423759) are being undertaken but they also use broad spectrum intravenous prophylaxis (e.g. imipenem) in case of FQ resistance. We therefore aim to develop an approach using oral antibiotic prophylaxis.

In American, European and Dutch urology guidelines, no clear recommendations are made on the duration of antimicrobial prophylaxis for transrectal prostate biopsy.(3-5) This provides urologists to choose their own dose and duration of prophylactic ciprofloxacin. A brief questionnaire send to over 20 Dutch urologists revealed the use of a wide range of prophylactic schedules. Ciprofloxacin was often prescribed for 2 or 3 days. A survey about the variability of pre-prostate biopsy prophylaxis among American urologists showed a lack of uniformity in prostate biopsy prophylaxis with 14 different duration regimens being used.(37) These findings suggest that inadequate and inappropriate antibiotic use is prevalent, since prolonged duration of prophylaxis in prostate biopsy is not proven to be more effective than a 1-day regimen.(2) Prolonged duration of prophylaxis is more likely to select for FQ resistant micro-organisms (38) which contributes to the observed increase of antibiotic resistance.

We therefore aim to reduce the duration of all antimicrobial prophylaxis to 1-day (except from pivmecillinam/­amoxicillin/clavulanic acid), in order to reduce the development of resistant bacteria. In addition, when effectively implemented, the targeted approach of this project might open up new possibilities for other surgical prophylactic indications.

*Appropriate diagnostics*

Appropriate methods to identify resistant rectal GNB may improve antimicrobial prophylaxis prescription by guiding clinicians in their decision making. As the rectal flora generally consists of a large variety of GNB, susceptibility testing of all individually cultured colonies is extremely time-consuming and expensive. In daily practice, a clear and rapid antibiotic prophylaxis advice is warranted. Previous studies on culture-guided prophylaxis for TRUS-PB already used a selective agar containing ciprofloxacin.(13, 23-25, 34, 36) Within 24 hours, a sterile agar plate then allows urologists to prescribe ciprofloxacin without any caution. However, in case of growth on the ciprofloxacin agar, these studies performed full susceptibility testing of all individually cultured bacterial colonies to find an alternative antibiotic for prostate biopsy prophylaxis. Again, this is expensive and time-consuming and, in our opinion, not suitable for routine diagnostics, especially when over 20% of cultures may contain FQ-resistant GNB. We therefore aim to go one step beyond and will develop four phenotypic screening agars to support the choice of the four oral prophylactic antibiotics used in this study (ciprofloxacin, trimethoprim/sulfamethoxazole, fosfomycin or pivmecillinam/amoxicillin/clavulanic acid). Such a screening approach is already available and proven effective for rectal screening of highly resistant microorganisms (HRMO) (e.g. MRSA, ESBL, CPE).(39) Our method is innovative as it has never been used for the purpose of targeting surgical prophylaxis. MediaProducts B.V. is a research partner willing to tailor make agars for commercial use, making it possible for all microbiological laboratories to use the same standardized and uniform diagnostic method of testing. Culture results become available rapidly, within 48 hours, and the method is simple and relatively inexpensive, as it does not need full susceptibility testing of separate colonies.

Our method also allows us to test the entire bacterial population in the rectum swab for the antibiotics in the agar. This is an advantage compared to traditional susceptibility testing (testing only one or a few colonies) as in case of heteroresistance, this will prevent the selection of resistant bacteria, leading to prophylactic failure.(40)

1. **OBJECTIVES**

**Primary Objective:**

1. To assess the effectiveness of rectal culture-guided antimicrobial prophylaxis for transrectal prostate biopsy (random ultrasound-guided, targeted MRI-guided or targeted MRI-ultrasound fusion guided) on infectious complications.

*We will perform a prospective non-blinded randomized controlled trial investigating the difference in post-prostate biopsy infection rate between patients receiving rectal culture-guided prophylaxis and patients receiving empirical prophylaxis with ciprofloxacin.*

*With this approach we aim to contribute to the control of antibiotic resistance by reducing infectious complications (less use of therapeutic antibiotics), prescription of prophylaxis based on rectal microbiome data and reducing the duration of prophylaxis to 24 hours (except for pivmecillinam/amoxicillin/clavulanic acid prophylaxis). Moreover, we will be able to compare the difference of infectious complications after random transrectal ultrasound-guided, targeted MRI and MRI-ultrasound fusion guided prostate biopsy.*

**Secondary Objectives:**

1. To compare the cost-effectiveness of empirical antimicrobial prophylaxis (standard of care) to rectal culture-guided antibiotic prophylaxis for transrectal prostate biopsy

*We aim to reduce the total costs of care of transrectal prostate biopsy using a rectal culture-guided prophylaxis strategy. This strategy has been advocated to decrease the rate of infections and hospital admissions related to FQ-resistant GNB. However, the increased expense of pre-biopsy rectal cultures and logistics must be weighed against the cost of more frequent antibiotic treatment and hospital admissions before any change in prophylaxis should be considered. We will do so by performing an empirical cost effectiveness analysis.*

1. To assess determinants (barriers and facilitators) that help or hinder the performance of the rectal culture-guided antibiotic prophylaxis strategy into daily practice.

*When assessing the (cost) effectiveness of the rectum culture-directed antibiotic prophylaxis strategy, it is important to take into account the experiences of patients and professionals regarding determinants that influence the performance of the approach in daily clinical practice. Such information is also important to facilitate the future implementation of rectum culture-directed antibiotic prophylaxis strategy into daily practice. For these reasons, an analysis of such determinants will be performed among patients and healthcare professionals. Based on this analysis, we will provide recommendations for daily practice.*

**3. STUDY DESIGN**

The project involves three main parts.

**3.1 Part I: Randomized controlled trial investigating the difference in infection rate after transrectal prostate biopsy between patients receiving rectal culture-guided antimicrobial prophylaxis and patients receiving empirical prophylaxis with ciprofloxacin.**

3.1.1 Overall trial design

This is a prospective, open-label, randomized, comparative, multi-center trial. Men suspect of prostate cancer, undergoing transrectal prostate biopsy as part of standard of care, will be included after written informed consent is obtained. Patients will be recruited at different study sites. We intend to include 1332 patients, 666 patients in both study arms:

- Group 1: Control group receiving routine empirical prophylaxis with ciprofloxacin (500 mg orally 2 hours before and 12 hours after the procedure) .
- Group 2: Intervention group receiving rectal culture-guided prophylaxis for transrectal prostate biopsy. Men whose rectal swabs do not show ciprofloxacin-resistant bacteria will receive ciprofloxacin prior to biopsy (equal to the control group), and men whose swabs do show ciprofloxacin-resistant bacteria will receive alternative oral antibiotics based on culture results in the following order:

1. trimethoprim/sulfamethoxazole (SXT) 960 mg orally 2 hours before the procedure and again 12 hours later, or
2. fosfomycin 3 g orally 2 hours before the procedure, or
3. pivmecillinam/amoxicillin/clavulanic acid respectively 400 mg and 500/125 mg 2 hours before biopsy, followed by 2 days with three divided doses each day after biopsy.

- Patients will be followed until 30 days after transrectal prostate biopsy for any registered clinical infectious complication (UTI, pyelonephritis, sepsis, fever, acute prostatitis, acute epididymitis), positive microbiological urine or blood culture results (if applicable), hospitalization, overall antibiotic use, side effects and toxicity of used antibiotics and overall mortality.
- Our hypothesis is that the intervention group will have a significant reduction of post-biopsy infectious complications compared to the control group (respectively 1.0% and 3.2%).
- Enrolment is expected to take place between April 1th, 2018 and February 1th, 2021.

3.1.2 Patient recruitment

*Process flow prior to transrectal prostate biopsy*

There is some variation in diagnostic work flow prior to prostate biopsy between the participating hospitals. We tried to uniform the logistic process flows between the participating hospitals, but also to deviate as little as possible from daily clinical practice. In Table 1 the general logistic process flow chart of the study prior to transrectal prostate biopsy is shown.

| **Table 1:** logistic process flow chart of the study prior to transrectal prostate biopsy | | |
| --- | --- | --- |
| **Time path:** | **Research activity:** | **Performed by:** |
|  | Patients are referred to the urology outpatient department (OPD) because of suspicion of prostate cancer.  - At the Isala hospital, all patients are directly scheduled for transrectal prostate biopsy without pre-biopsy OPD visit.  - At the Radboudumc, all patients are first seen at the urology OPD. On the same day a MRI prostate is performed. Three working days later, depending on the outcome of the MRI, patients will undergo transrectal prostate biopsy. This interval is too short to perform all the study activities (e.g. rectal cultures). Therefore, in the Radboudumc, we will recruit patients prior to OPD visit and MRI prostate. Patients with a normal MRI (approximately 50%) may not need to undergo transrectal prostate biopsy and will be excluded from futher participation in the study (see below).  - At the other hospitals, after referral, patients are first seen at the urology OPD to determine if transrectal prostate biopsy is indicated. | Urologist |
| Approximately14 days prior to biopsy | At the Radboudumc and Isala hospital, patients will receive the following items per post.  Patient information letter  Informed consent form  Rectal swab with instructions  Retour envelopes  In the other hospitals, during pre-biopsy OPD visit, patients are informed about the study and eligibility for participation is assessed. In case participation in the study is considered, patients will receive the above items at the urology OPD. | Someone of treatment team |
| Approximately 12 days prior to biopsy | If wanted, patients are contacted by telephone to offer them the possibility to ask questions about the study after reading the patient information letter. | Someone of the research team |
| 10 - 12 days prior to biopsy | 1. The informed consent form must be appropriately signed and dated and returned to the research team. The signed informed consent form will remain in the study file and be available for verification by study monitors at any time.  N.B. some patients will not sign the informed consent form in presence of the investigator. This is in the interests of the patients, namely to make logistics as easy as possible for them (no additional OPD visit) and to allow them sufficient time to consider the information. Moreover, we want to deviate as little as possible from daily clinical practice in order not to influence the barrier analysis. The investigator will sign the informed consent form after the patient has signed. | Patient |
|  | 2. The rectal swab will be sent to the Medical Microbiology laboratory within 24 hours after (self-)sampling.*  N.B. in case only a rectal Eswab is sent to the laboratory without receiving an informed consent form, the rectal Eswab will be distroyed. In case only the informed consent form is received without rectal Eswab, patients will be called to ask to sent the rectal Eswab after all. However, if even after this reminder no rectal Eswab is received, patients will be included into the study (per protocol analysis). |  |
| 9 days prior to biopsy | After informed consent is obtained, the following items are sent to the patient by e-mail or by post:   - Questionnaire about demographic parameters, use of medication (including antibiotics), allergies and other parameters which might intervene with the study outcome - EQ-5D-5L (to assess health status) - iMTA PCQ (to assess productivity loss) - IPSS questionnaire - Copy of the signed (by patient and investigator) informed consent form   In case of uncertainties patients will be called.  Analysis of determinants in a subgroup of patients:  Phase 1: interviews to identify factors that influence the performance of the targeted prophylaxis strategy (approximately 5-10 patients per center). Patients will receive a patient information letter and informed consent form to participate in the interviews.  Phase 2: tailored questionnaire (based on the results of phase 1) on factors that influence the performance of the targeted prophylaxis strategy (approximately 50-100 patients per center). | Someone of the research team |
|  | Study participation is noted in patient’s Electronic Medical Record. |  |
|  | Patient’s home pharmacy is contacted to obtain the following information:  current medication use  use of antibiotics within 1 year before prostate biopsy  history of allergy to antibiotics |  |
| > 6 days  prior to biopsy | Rectal swabs are cultured on 4 selective agar plates containing concentrations of the oral antibiotics used in the study. | Microbiology lab of the study site |
| 5 days prior to biopsy | *In the Radboudumc,at this point patients with a normal MRI of the prostate, who do not need to undergo transrectal prostate biopsy, will be excluded from further participation in the study. The gathered information (from the above mentioned questionnaires and the rectal swab) will be used to determine the relation between antibiotic resistance in the fecal carriage and other determinants of infection after transrectal prostate biopsy.* |  |
|  | Randomization is performed in Castor (data management system). | Study coördinator |
|  | Antibiotic prophylaxis is prescribed and the receipt is sent to the Clinical Trial unit of the pharmacy of the concerning partipating center. Participants will be offered two choices: to obtain the antimicrobial prophylaxis in the days before prostate biopsy at the outpatient department Urology (consequence: extra trip to the hospital) or to be present in the hospital two hours prior to prostate biopsy and then obtain the antimicrobial prophylaxis at the outpatient department Urology (and take the antimicrobial prophylaxis immediately).  - The type of antibiotic prophylaxis is noted in patient’s Electronic Medical Record.  - Medication monitoring will be performed by the study coordinator.  Nota Bene:  - The study coordinator will have access to the laboratory management systems and thereby be able to see the results of the rectum swab cultures.  - Pivmecillinam, although registrerd, is not available in the Netherlands, because of a too small market share for this product. Therefore pivmecillinam will be imported from Germany by the Clinical Trials Unit of the Radboudumc and from there be provided to the Clinical Trials Units of the pharmacies in the other participing centers.  - In case of resistance to all oral antibiotics in the intervention group, the study coordinator will phone the subject to inform on the administration of a combination of two oral antibiotics (preference) or intravenous prophylaxis prior to transrectal prostate biopsy (based on rectal swab culture results). |  |
| Immediately before biopsy | Another rectal (control) swab is taken by the treating urologist and sent to the Medical Microbiology laboratory of the study site within 24 hours after sampling, so that in case of infectious complications, an attempt can be made to determine the cause of the infectious complication (see page 37).  Moreover, the second rectal Eswab provides insight into the direct effect of antibiotic prophylaxis on the intestinal flora. Such information has not been described in the literature and is very useful and relevant.  The urologist will check if antibiotic prophylaxis is used adequately. | Urologist |
|  | **Transrectal prostate biopsy is performed.** | **Urologist** |

* Self-sampling has been proven equally effective compared to swabs collected by clinicians in other patient groups (41, 42) and it has been shown that screening two weeks before biopsy provides concordant results compared with testing immediately before biopsy.(43)

*Process flow after transrectal prostate biopsy*

In Table 2 the general logistic process flow chart of the study after transrectal prostate biopsy is shown.

| **Table 2:** logistic process flow chart of the study after transrectal prostate biopsy | | |
| --- | --- | --- |
| **Time path:** | **Research activity:** | **Performed by:** |
| 7 days after biopsy | To following items are sent to the subject by e-mail or by post:   - Questionnaire about study measurements - EQ-5D-5L (to assess health status) - iMTA PCQ (to assess productivity loss)   In case of signs of infection or uncertainties patients will be called. | Someone of the research team |
| 30 days after biopsy | To following items are sent to the subject by e-mail or by post:   - Questionnaire about study measurements - EQ-5D-5L (to assess health status) - iMTA PCQ (to assess productivity loss)   In case of signs of infection or uncertainties patients will be called. | Someone of the research team |
| > 30 after prostate biopsy | Medical records will be checked for infectious complications, medical history, infection related parameters, prostate biopsy related parameters, missing data (see 6.1.3) | Study coördinator |
|  | Microbiological results of urine and blood cultures within 30 days after transrectal prostate biopsy, available at the hospital microbiological laboratory will be assessed using the laboratory management system. Cultures are only obtained when patients seek medical attention, in case of signs and symptoms of infection (standard practice). Blood and urine cultures are thus not taken specific for this study. Patients are asked to visit their treating urologist instead of their general practioner in case of infection (already standard practice), to ensure all information and corresponding cultures are delivered to the study site. However, in case of treatment by the general practioner or referral to a hospital which is not a study site, medical records will be checked for data (included in the informed consent form). |  |
|  | The study coordinator will check if the E-CRF is complete. | Study coordinator |

3.1.3 Prophylaxis schedule for the intervention group

Decision making after culture results of the rectum swab become available is illustrated in Figure I. Based on the level of evidence, ciprofloxacin remains our first choice prophylaxis in the intervention group. As a result, based on Dutch ciprofloxacin resistance in *E. coli*, an estimated 70-80% of the intervention group will still receive ciprofloxacin prophylaxis.

When a patient’s rectum swab harbours FQ resistant isolates (estimated 20-30% of the intervention group), trimethoprim/sulfamethoxazole (SXT) will be prescribed. When resistant to both FQ en SXT, fosfomycin will be prescribed. When resistant to FQ, SXT and fosfomycin, pivmecillinam/amoxicillin/clavulanic acid will be prescribed. When resistant to all 4 antibiotics, full susceptibility testing of all isolates on the ciprofloxacin agar is performed and a combination of ciprofloxacin with another oral antibiotic (SXT, fosfomycin, pivmecillinam/amoxicillin/clavulanic acid) will be given. As last option intravenous antibiotics (ceftriaxone, meropenem) will be prescribed based on susceptibility testing of isolates that grow on the control agar.

Only culture results from patients in the intervention group will be used to target prophylaxis. Culture results of the control group will be saved in the study E-CRF but are not used to guide antibiotic prophylaxis for transrectal prostate biopsy.


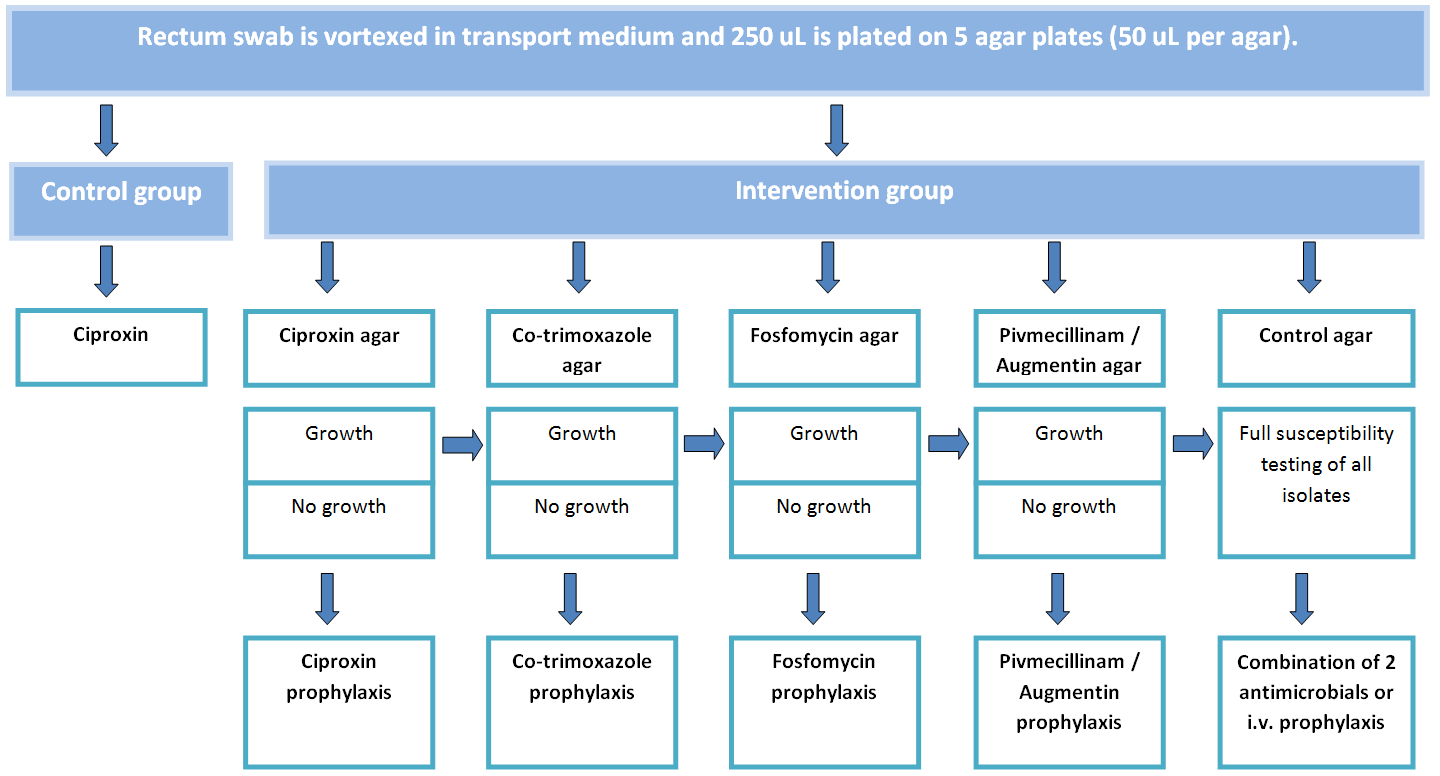


*Figure I. Flowchart illustrating decision-making after culture results become available in the intervention group.*

**3.2 Part II: Cost-effectiveness analysis (CEA) of the culture-guided antibiotic prophylaxis strategy**

We will compare the cost-effectiveness of full implementation of rectal swab culture-guided antimicrobial prophylaxis in men undergoing transrectal prostate biopsy to the usual standard of care (empirical antimicrobial prophylaxis with ciprofloxacin). This will be done from a societal perspective. The design of the economic evaluation follows the principles of a cost-utility analysis and adheres to the new Dutch guideline for performing economic evaluations in health care.(44)

Cost-effectiveness will be expressed in terms of cost per QALY gained. The empirical CEA timeframe will be 30 days after prostate biopsy. We hypothesize that there will be less biopsy (infectious) related complications in the intervention arm within 30 days after transrectal prostate biopsy, which will result in a reduction of health care consumption, increased work resumption/participation and an increase in health related quality of life in this target population.

*Cost analysis*

The cost analysis exists of two main parts. First, on patient level, health care consumption related to infectious complications after prostate biopsy such as antibiotics, hospital admission and ICU admission, will be measured prospectively, using patient questionnaires, medical records, E-CRFs (see part I) and if necessary data from the hospital administration system. In addition, all costs related to the culture-guided approach are calculated as well, consisting of costs of lab materials and hands-on time of laboratory personnel.

Second, per item of health care consumption standard cost-prices will be determined using appendix 1 of the guideline for performing economic evaluations.(44) If standard prices are not available, activity based costing will be used to allocate full cost prices to items. To estimate the indirect costs of transrectal prostate biopsy related infectious complications, the friction cost method will be applied following the Dutch guidelines.(44) Productivity losses for patients will be assessed using the iMTA PCQ.(45) In addition, travel time to the hospital and related costs will be considered. Differences in cost between the intervention group and the control group will be evaluated using regression based techniques.

*Patient outcome analysis*

To assess patient’s health status, a validated health-related quality of life (HRQoL) instrument, the EuroQol-5D (EQ-5D-5L)(46) will be used at baseline, 7 days and 30 days after transrectal prostate biopsy. This instrument comprises five domains of health: mobility, self-care, usual activities, pain/discomfort and anxiety/depression and provides a societal-based global quantification of patient’s health status on a scale ranging from 0 (death) to 1 (perfect health). Patients will also be asked to rate their overall HRQoL on a visual analogue scale (EQ-5D VAS) consisting of a vertical line ranging from 0 (worst imaginable health status) to 100 (best imaginable).

**3.3 Part III: Assessment of determinants that help or hinder the performance of the culture-based approach in daily clinical practice**

We will assess experiences of patients and professionals regarding barriers and facilitators (determinants) that influence the performance of the culture-guided approach in daily clinical practice. Such information is important to take into account when assessing the (cost) effectiveness of rectum culture-directed antibiotic prophylaxis strategy and to facilitate its future implementation into daily practice. We will conduct interviews with patients and, once the trial period has ended, with healthcare professionals involved in the performance of the culture-guided approach (urologists, microbiologists and residents who prescribe antimicrobial prophylaxis for transrectal prostate biopsy, nurses) to identify factors that influence the performance of the targeted prophylaxis strategy. For a preliminary topic list, see Appendix 1. The obtained information will be used to translate available determinant frameworks (the Dutch validated measurement for determinants of innovations (MIDI)(47) framework combined with a checklist for identifying determinants of practice (i.e. a synthesis of frameworks and taxonomies of factors that prevent or enable improvements in healthcare professional practice))(48) into tailored questionnaires.

Next, patients of the participating centers will be asked to fill in this tailored questionnaire. Moreover, an (online) survey will be performed among Dutch professionals. Based on this information recommendations on the performance of the culture-guided approach will be developed.

Protocol timeline:

Part I: RCT and data analysis: 31 months

Part II: Cost-effectiveness analysis: 6 months

Part III: Barrier analysis: 12 months

Part II and III, to some extent, can run in parallel with part I.STUDY POPULATION

**4. STUDY POPULATION**

4.1 Population (base)

All men scheduled for transrectal prostate biopsy from April 1, 2018 until at least 1322 patients have been included in the study will be enrolled. Identification of eligible patients will be performed after patients are referred to the urology OPD because of an elevated prostate-specific antigen (PSA) and/or abnormal digital rectal examination indication possible prostate cancer. Patients will be recruited in the participating hospitals, see page 5.

4.2 Inclusion criteria

In order to be eligible to participate in this study, a subject must meet all of the following criteria:

Subject undergoes a transrectal prostate biopsy as part of the standard care in one of the participating centers (because of suspicion of prostate cancer)

Subject is able and willing to sign the Informed Consent Form

4.3 Exclusion criteria

A potential subject who meets any of the following criteria will be excluded from participation in this study:

- Inability to receive ciprofloxacin prophylaxis for any reason
  - documented history of sensitivity to medicinal products or excipients similar to those found in the antibiotics used as prophylaxis
  - relevant history or presence of cardiovascular disorders (specific relevant Qtc time prolongation)
  - relevant drug interaction
  - an increased risk of fluoroquinolone-induced tendinitis or tendon rupture (specifically patients with decreased renal function defined as MDRD <30 ml/min/1.73m^2^, patients with solid organ transplants, concomitant use of corticosteroids or a history of fluoroquinolone-induced tendinopathy).
  - an increased risk of aortic anneurysm or dissection (specifically patients with a connective tissue disease (e.g. Marfan syndrome, Ehlers-Danlos syndrome, large vessel arteritis (Tayasu, gaint cell), Behçet disease), patients with a history of aortic aneurysm or dissection or patients with a family history (siblings) of aortic aneurysm or dissection).
- Inability to receive either SXT, fosfomycin and pivmecillinam/amoxicillin/clavulanic acid prophylaxis for any reason (e.g. documented history of sensitivity to medicinal products or excipients similar to those found in the antibiotic prophylaxis, relevant drug interaction)
- Inability to understand the nature of the trial and the procedures required
- Subjects with UTI or acute prostatitis within 14 days prior to transrectal prostate biopsy
- Subjects who received antibiotics within 14 days prior to transrectal prostate biopsy
- Subjects who fail to send a rectum swab to the microbiology laboratory
- Subjects whose rectal swab shows no growth on a Colombia blood agar (growth control)
- Repeat within seven days

Subjects who cannot receive ciprofloxacin are not able to participate in the control group and are most probably (since approximately 70-80% of isolates is ciprofloxacin susceptible) unable to participate in the intervention group as well. Individuals that cannot receive either SXT, fosfomycin and pivmecillinam/amoxicillin/clavulanic acid are not able to participate because these antibiotics are the alternative choices in case of ciprofloxacin resistance in the intervention group. Subjects with UTI or acute bacterial prostatitis pre-procedure are excluded because of inability to differentiate between pre- or post-biopsy infection. When antibiotics are administered within 14 days before biopsy rectal sampling results might become unreliable, as these are taken within the same period. Subjects whose rectal swab shows no growth on a Colombia blood agar (growth control), are excluded because then the rectal swab specimen is most likely inadequate, probably incorrect suggesting FQ sensitivity of rectal flora.

4.4 Sample size calculation*

This study will be conducted in three hospitals. In the Radboudumc Nijmegen approximately 200 targeted MRI-guided or MRI-ultrasound fusion guided prostate biopsies are performed annually. In the Canisius Wilhelmina hospital Nijmegen and Catharina hospital Eindhoven approximately 300 random ultrasound-guided prostate biopsies are performed annually per center.*****

To perform an adequate sample size calculation, we used our own retrospective data about infectious complications after transrectal ultrasound-guided prostate biopsy and searched for infectious complication rates in the literature. A search in the Dutch pathology database resulted in 1742 unique histological biopsy numbers from 842 biopsy procedures/patients performed in the Radboudumc between 2014 and 2015. We linked patient numbers from this database to patient numbers in the microbiology database of the Radboudumc and searched for positive urine and blood cultures obtained within 30 days after TRUS-PB. We only included urine cultures with both GNB and gram stain results that were suspected for a true UTI. We found positive urine cultures in 2.73% of patients and positive blood cultures in 0.6% of patients. However, we believe this is an underestimation of the true infectious complication rate after TRUS-PB procedures because of the retrospective nature of our search, the microbiological endpoint (not all clinical infections are cultured) and the potential lack of cultures from general practitioners and other hospitals that were not sent to our microbiology laboratory. Another Dutch retrospective study was performed using fever as clinical outcome and found 3% infections among 706 patients that underwent TRUS-PB in 2011.(6) Other studies that assessed infectious complications after empirical ciprofloxacin prophylaxis for TRUS-PB in the last decade found overall infectious rates between 2.5% and 5.5% with similar ciprofloxacin resistance rates of approximately 20%.(11-17) In the literature, infectious complications after TRUS-PB in patients with ciprofloxacin susceptible rectal flora varied between 0% and 1.6%.(10, 11, 26) Studies that used a culture-guided strategy found lower complication rates of less than 1%, although designs of these studies were not robust and often lacked power.(13, 24, 34).

The sample size calculation in the original protocol was based on the assumption that 200 patients would be included at the Radboudumc with lower infectious complications rates because less core samples are taken with MRI-guided or MRI-ultrasound fusion transrectal prostate biopsy. Because the recruitment rate in the Radboudumc was considerably lower than expected (30 patients included instead of the expected 200 patients), we decided (after consultation with the statistician) to go back to the original power calculation (of the grant proposal and previous versions of the study protocol) in order to prevent the (unnecessary) waste of research grant and (unnecessary) patient burden:

Sample size calculation is based on an estimated suspected complication rate in the control group (3.2%) and suspected reduction of infectious complications to 1.0% using a culture-guided approach. We calculated a planned enrollment of 1332 patients, 666 patients in both groups to achieve 80% power to detect a difference between the group proportions of -2.2% (to detect a reduction of 2.2%). The proportion in the intervention group is assumed to be 3.2% under the null hypothesis and 1% under the alternative hypothesis. We used the two-sided Fisher's Exact test, with significance level targeted at 5%.

***Note: due to changes in the daily clinical practice around prostate biopsy in some hospitals and delay in the start of this clinical trial, the recruitment rate is lower as expected. Therefore, the trial is extended with more participating hospitals.**

**5. TREATMENT OF SUBJECTS**

For a detailed description of the study design, see Chapter 3

The intervention consist of a rectal swab taken by self-sampling and sent to the microbiological laboratory of the study site prior to transrectal prostate biopsy. Culture results of the rectum swab about antibiotic resistance in the fecal carriage will be used to guide antimicrobial prophylaxis in the intervention group. Antimicrobial prophylaxis can then be tailored to each patient individually.

A second rectal swab will be taken by the urologist immediately before prostate biopsy, so that in case of infectious complications, an attempt can be made to determine the cause of the infectious complication (see page 37). Moreover, the second rectal Eswab provides insight into the direct effect of antibiotic prophylaxis on the intestinal flora. Such information has not been described in the literature and is very useful and relevant.

The (cost-)effectiveness of this rectal culture-guided antimicrobial prophylaxis strategy for transrectal prostate biopsy (on infectious complications) will be compared with the use empirical antimicrobial prophylaxis with ciprofloxacin (standard of care).

**6. INVESTIGATIONAL PRODUCT**

Overview

See chapter 3 for a detailed description of each study arm and antibiotic prophylaxis flow chart.

Identity of investigational products

All antibiotics used in this study as antimicrobial prophylaxis for transrectal prostate biopsy are commercially available and registered in the Netherlands. There are no special storage requirements for the antibiotics used in this study.

The following formulations will be used:

*Current standard of antibiotic prophylaxis for transrectal prostate biopsy*

Name: Ciprofloxacin 500 mg, film-coated tablets

Active ingredient: Ciprofloxacin (as hydrochloride monohydrate)

Excipients: Core: cellulose, microcrystalline, sodium starch glycollate (type A), povidone, silica, colloidal anhydrous stearic acid magnesium stearate croscarmellose, sodium.

Film: hypromellose, macrogol 600, talc, titanium dioxide (E 171)

Dosage: 500 mg orally 2 hours before and 12 hours after transrectal prostate biopsy

*Culture-guided antibiotic prophylaxis for transrectal prostate biopsy (intervention group)*

Name: Co-trimoxazole 160/800 mg, tablets

Active ingredient: Each tablets contains 160 mg trimethoprim and 800 mg sulfamethoxazole

Excipients: Povidone, sodium starch glycollate, magnesium started, docusate sodium

Dosage: trimethoprim/sulfamethoxazol 960 mg orally 2 hours before and 12 hours after transrectal prostate biopsy

Name: Fosfomycin 3 g, granules for oral solution

Active ingredient: Each single-dose sachet contains 5631 mg fosfomycin trometamol, equivalent to 3 g

fosfomycin

Excipients: Orange flavour consisting of maltodextrin, dextrose monohydrate, acacia (E414),

anhydrous citric acid (E330), butylhydroxyanisole (E320)

Dosage: fosfomycin 3 g orally 2 hours before transrectal prostate biopsy

Name: Pivmecillinam hydrochloride 200 mg, film-coated tablets

Active ingredient: Pivmecillinam hydrochloride

Excipients: Core: cellulose microcrystalline, magnesium stearate

Film: Hypromellose, triacetin

Dosage: pivmecillinam 400 mg orally 2 hours before transrectal prostate biopsy, followed by 2 days with three divided doses each day after biopsy (combined with amoxicillin/clavulanic acid)

Manufacturer: LEO Pharma A/S

Name: Amoxicillin/clavulanic acid 625 mg, film-coated tablets

Active ingredient: Each film-coated tablet contains amoxicillin trihydrate equivalent to 500 mg amoxicillin and potassium clavulanate equivalent to 125 mg of clavulanic acid

Excipients: Core: magnesium stearate, sodium starch glycolate (type A), colloidal anhydrous silica, microcrystalline cellulose

Film: titanium dioxide (E171), hypromellose, macrogol (4000, 6000), dimeticone

Dosage: amoxicillin/clavulanic acid 500/125 mg orally 2 hours before transrectal prostate biopsy, followed by 2 days with three divided doses each day after biopsy (combined with pivmecillinam)

Explanation of antibiotics choice and duration for this study

All antibiotics in this study were chosen because of potent activity against a broad spectrum of clinically relevant pathogens in the urogenital tract (see Summary of Product Characteristics (SPC) of the individual antibiotics) and rapid penetration and high concentration in prostatic tissue.(49-52) The antibiotics are registered in the Netherlands, can be administered orally and their potential effectiveness has been shown in previous studies.(19, 53-61) Furthermore, fosfomycin and pivmecillinam are not commonly prescribed in hospitals, which diminishes the pressure on antibiotics used in hospitals.

In American, European en Dutch urology guidelines, no clear recommendations are made on the duration of antimicrobial prophylaxis for transrectal prostate biopsy.(3-5) Therefore, various prophylactic ciprofloxacin schedules are used (till 7 days of prophylaxis), of which 2 to 3 day regimens are most common. There is no evidence that supports superiority of a long-course regimen (2), except for pivmecillinam/amoxicillin/clavulanic acid for which extended prophylaxis was proven to be more effective.(19) Since prolonged antimicrobial prophylaxis use does have clear disadvantages (side effects and selection of resistant microorganisms are more likely), we believe that antimicrobial prophylaxis, with exception of pivmecillinam/amoxicillin/clavulanic acid, should not exceed one day.

1. **Ciprofloxacin**

Ciprofloxacin is a broad-spectrum antibiotic of the fluoroquinolone class. Ciprofloxacin is active against many gram-positive and gram-negative bacteria and has rapidly bactericidal activity and high potency.

Ciprofloxacin is well absorbed after oral administration and has excellent penetration into the prostate gland.(49) Ciprofloxacin is registered for the treatment of acute bacterial prostatitis, has been recommended for transrectal prostate biopsy prophylaxis in current guidelines (3-5) and remains the most commonly used prophylactic antimicrobial agent for transrectal prostate biopsy due to the demonstrated benefit in reducing infectious complications in many studies.(2, 53, 54)

For more information about ciprofloxacin, see Summary of Product Characteristics.

1. **Trimethoprim/sulfamethoxazole (SXT)**

SXT is a drug combination with broad-spectrum, bactericidal activity against both gram-positive and gram-negative organisms and is registered for the treatment of uncomplicated UTI. It has been mentioned in the Dutch and European guidelines as alternative to ciprofloxacin as a prophylaxis in transrectal prostate biopsy.(4, 5) SXT has good penetration into prostatic tissue and has been proven equally effective to ciprofloxacin in previous studies.(2) Comparably high or even higher levels of resistance in mainly *E. coli* make it difficult to use SXT as empiric prophylaxis for transrectal prostate biopsy. In 2016, SXT resistance rates in *E. coli* from urology OPD and inpatient departments in The Netherlands were 29% and 31%, respectively.(21) A search in our hospital laboratory system (Glims) between 2014 and 2016 revealed that 33% of ciprofloxacin-resistant GNB isolates from patients that visited the urology OPD (n=805) remained susceptible to SXT. Therefore, we believe that SXT might be used as an alternative to ciprofloxacin in case of FQ resistance.

For more information about SXT, see Summary of Product Characteristics.

1. **Fosfomycin**

Fosfomycin is increasingly considered as alternative prophylaxis to ciprofloxacin for transrectal prostate biopsy in countries with high rates of FQ resistance. It is a broad-spectrum, bactericidal antibiotic, registered for the treatment of uncomplicated UTI, with good activity against *Enterobacteriaceae,* especially *E. coli*. Nethmap (21) does not report fosfomycin resistance rates, but in the Radboudumc, fosfomycin resistance in *E. coli* from cultures of the urology OPD and inpatient department was lower than 3% between 2012 and 2016. Fosfomycin is well tolerated and has a low incidence of harmful side effects. After a single dose, high concentrations in urine (2000 mg/L) are reached. Last years, fosfomycin is increasingly prescribed in acute prostatitis. Pharmacological studies have shown relatively good prostate concentrations of fosfomycin after a single dose of 3 gram.(51, 52) The first trial published on fosfomycin prophylaxis in TRUS-PB patients, compared single-dose fosfomycin to single-dose levofloxacin or ciprofloxacin (5 days). A 50% reduction in febrile UTI was observed in the fosfomycin group compared to both FQ groups.(61) Also, three recent studies, of which one with 1109 patients, showed that antibiotic prophylaxis with fosfomycin had a significantly lower rate of infectious complications compared to ciprofloxacin.(57, 59, 60, 62)

For more information about fosfomycin, see Summary of Product Characteristics.

1. **Pivmecillinam/amoxicillin/clavulanic acid**

Pivmecillinam is an orally active prodrug of mecillinam, an extended-spectrum penicillin. It is only considered to be active against gram-negative bacteria (including ESBLs), has low resistance rates in GNB and is registered for the treatment of UTI in the Netherlands since 2015. Although it is recommended for the treatment of uncomplicated cystitis in the European Association of Urology Guidelines,(4) it is mainly used for this indication in Nordic countries.(63) Side effects are considered mild and similar to other penicillins. Synergy between pivmecillinam and clavulanic acid has been documented and the combination decreases the MIC of mecillinam over 32 fold in ESBL *E. coli*. As the combination pivmecillinam/clavulanic acid is not available as a product, pivmecillinam with amoxicillin/clavulanic acid is administered. Antsupova *et al.*(19) retrospectively compared prophylactic ciprofloxacin monotherapy (1 day) to pivmecillinam/amoxicillin/clavulanic acid short-course (1 day) and long-course (3 days) in a large series of 2624 Danish men undergoing TRUS-PB. In the pivmecillinam/amoxicillin/clavulanic acid long-course group, the bacteremia rate significantly decreased compared to pivmecillinam/amoxicillin/clavulanic acid short-course and ciprofloxacin. Also, a stabilization of ciprofloxacin resistance with no further increase was observed and a significant decrease in the number of ESBLs.

We believe pivmecillinam in combination with amoxicillin/clavulanic acid may be a promising oral antimicrobial option for prophylaxis of infectious complications in men undergoing TRUS-PB. It has good activity against GNB including ESBLs, reasonable penetration into prostatic tissue (50), few side effects, most probably a lower risk of resistance development compared to ciprofloxacin, and there is much experience with the product in other European countries.

A disadvantage is the relatively broad spectrum of antimicrobial activity when it is combined with amoxicillin/clavulanic acid (compared to the other products used in this study). Moreover, pivmecillinam is not available in Dutch pharmacies because of a too small market share for this product in the Netherlands and needs to be imported from Germany for this study. Therefore, in case a subject after randomization, according to the research protocol, should receive pivmecillinam/amoxicillin/clavulanic acid as antimicrobial prophylaxis, the antibiotics will be delivered by courier from the pharmacy of the Radboudumc to patients home address. In these cases medication verification and monitoring will be performed by the study coordinator by requesting an overview of participant’s (concomitant) medication at the home pharmacy (included in the informed consent form).

Because of the above information, we chose to use pivmecillinam/amoxicillin/clavulanic acid as the last option in the intervention group (see chapter 3). Therefore, only a small amount of participants will receive these antibiotics (estimated at a maximum of 100 participants).

For more information about pivmecillinam or amoxicillin/clavulanic acid, see Summary of Product Characteristics.

6.4 Summary of known and potential risks

See the Summary of Product Characteristics of the individual antibiotics.

Preparation and labelling of Investigational Medicinal Product

The study coordinator will perform randomization and thereafter, will prescribe antimicrobial prophylaxis according to the research protocol. Medication will be purchased centrally by the Clinical Trials Unit of the pharmacy of the Radboudumc, labelled according to Annex 13 legislation and from there be provided to the Clinical Trials Units of the pharmacies of the other participating hospitals. Study medication will distributed and stored according to GMP. Pivmecillinam, although registered, is not available in the Netherlands, because of a too small market share for this product. Therefore pivmecillinam will be imported from Germany. Trial medication will be supplied as the commercially available registered antibiotics. Medication verification and monitoring will be performed by the study coördinator by requesting an overview of participant’s (concomitant) medication at the home pharmacy (included in the informed consent form).

Drug accountability

The study coordinator or her designee must maintain an adequate record of the medical prescriptions. This record must be available for inspection at any time.

Drug accountability will be performed by the Clinicial trials units of the hospital pharmacies.

Prior to prostate biopsy, the urologist will ask and note if the participant has correctly taken the antibiotic prophylaxis (already routine practice).

Blinding

As this is an open trial, blinding procedures are not applicable.

**7. METHODS**

**7.1 Study parameters/endpoint**

7.1.1 Main study parameter/endpoint

- Any registered clinical infectious complication (UTI, pyelonephritis, sepsis, fever, acute prostatitis, acute epididymitis) within 7 days after transrectal prostate biopsy.
- UTI is defined as pyuria (>5 leucocytes per high-powered field) and bacteriuria (≥10^3^ colony-forming units/ml) with symptoms of dysuria, urgency, frequency or hematuria.
- pyelonephritis is defined as pyuria and bacteriuria with symptoms of fever, flank pain, nausea or vomiting.
- fever is defined as a temperature ≥38.0°C.
- sepsis is defined as suspected infection plus at least two of the following criteria (qSOFA score): low blood pressure (SBP ≤100 mmHg), high respiratory rate (≥22 breaths per min), or altered mentation (Glasgow coma scale <15).(64)
- severe sepsis or septic shock is defined as sepsis plus organ dysfunction or with persisting hypotension requiring vasopressors to maintain MAP ≥65 mmHg and to have a serum lactate level <2 mmol/L despite adequate volume resuscitation.
- acute prostatitis is defined as fever, bacteriuria and/or pyuria, and a painful prostate.
- acute epididymitis is defined as the presence of a swollen, red or warm scrotum and pain for which antibiotics are prescribed.

7.1.2 Secondary study parameters/endpoints

- Cost of care 30 days after transrectal prostate biopsy to determine and compare overall costs of care among the intervention- and the control group.
- Positive microbiological urine or blood culture results within 7 and 30 days after transrectal prostate biopsy.
- Bacteremia: defined as the presence of bacteria in the blood culture, accessed due to protocol blood collection, irrespective of clinical signs.
- Bacteriuria: the presence of bacteria (uropathogens) in the urine of ≥10^3^ colony forming units/ml obtained in the period after transrectal prostate biopsy, irrespective of clinical signs.
- Clinical infectious complications (as for primary endpoint) within 30 days after transrectal prostate biopsy.

7.1.3 Exploratory

- Hospitalization, including ICU admission and length of admission, within 30 days after transrectal prostate biopsy.
- Overall mortality within 30 days after transrectal prostate biopsy.
- Side effects and toxicity of used antibiotics within 30 days after transrectal prostate biopsy.
- Prevalence of ciprofloxacin resistant GNB in local rectal flora, assessed through microbiological rectal swab cultures.
- Overall antibiotic use within 30 days after transrectal prostate biopsy.
- The relation between antibiotic resistance in the fecal carriage and other determinants of infection after transrectal prostate biopsy.
- Experiences of patients and healthcare professionals regarding barriers and facilitators (determinants) that influence the performance of the culture-guided approach in daily clinical practice.

**7.1.4 Other study parameters**

The following data will be collected prospectively:

***Prior to transrectal prostate biopsy***

*Informed consent conversation*

During the informed consent conversation, information about possible exclusion criteria will be obtained e.g. allergies, medication use, and recent infections.

*Questionnaires:*

Before transrectal prostate biopsy, after informed consent is obtained, all participants have to fill in some questionnaires (sent by e-mail or post).

1. *Questionnaire for the purpose of gathering information on demographic data and parameters which might intervene with the study outcome.*

The following data are collected:

- age, length, body weight, BMI
- history of smoking
- International Prostate Symptom Score (screening tool used to screen for lower urinary tract symptoms, including feeling of incomplete bladder emptying, frequency, intermittency, urgency, weak stream, straining, nocturia and quality of life.(65, 66)
- history of previous transrectal prostate biopsy and antimicrobial prophylaxis used
- treatment or history of treatment by medical specialists in which hospital
- history of recent UTI or acute prostatitis < 14 days prior to transrectal prostate biopsy
- history of allergy to antibiotics
- use of antibiotics within 1 year before prostate biopsy (type and duration)
- hospitalization within 1 year before prostate biopsy, including date and length of admission
- international travel within 6 months before transrectal prostate biopsy including destination (especially Southeast Asia or South America or South Europe)
- presence of permanent urinary catheterization or intermittent catheterization
- instrumental investigation of the urinary tract < 30 days prior to transrectal prostate biopsy
- occupation as health worker

1. EQ-5D-5L questionnaire (46): a validated heath-related quality of life instrument to assess patient’s health status for the purpose of the cost-effectiveness analysis of the culture-guided antibiotic prophylaxis strategy.
2. iMTA PC questionnaire (70): to assess productivity loss (baseline) for the purpose of the cost-effectiveness analysis of the culture-guided antibiotic prophylaxis strategy.
3. Analysis of determinants among patients:

Phase 1: interviews to identify factors that influence the performance of the targeted prophylaxis strategy (approximately 5-10 patients per center, for approximately 1 hour per patient). Patients will receive a patient information letter and informed consent form to participate in the interviews. NB these documents and the interview guide will be presented separately (i.e. once they are ready) for CCMO approval. For a preliminary topic list, see Appendix 1.

Phase 2: tailored questionnaire (based on the results of phase 1) on factors that influence the performance of the targeted prophylaxis strategy (approximately 50-100 patients per center, for approximately 15 minutes per patient). NB Again, this questionnaire will be presented separately (i.e. once they are ready) for CCMO approval.

*Microbiological characteristics:*

- Prior to transrectal prostate biopsy, a rectal swab is taken by self-sampling and sent to the microbiological laboratory. Microbiological results from rectal swab cultures (resistance of bacteria for ciprofloxacin, SXT, pivmecillinam/amoxicillin/clavulanic acid and fosfomycin) from all participants are collected.
- Immediately before transrectal prostate biopsy, another rectal (control) swab is taken by the treating urologist. This rectal swab will be stored at -70°C and only cultured in case of a clinical infectious complication despite adequate antibiotic prophylaxis in an attempt to determine the cause of the infectious complication. Moreover, the second rectal Eswab provides insight into the direct effect of antibiotic prophylaxis on the intestinal flora. Such information has not been described in the literature and is very useful and relevant.

*Information about medication:*

After informed consent is obtained, participants’ home pharmacy will be contacted to obtain the following information.

- current medication use
- use of antibiotics within 1 year before prostate biopsy
- history of allergy to antibiotics

***After transrectal prostate biopsy***

*Questionnaires:*

Approximately 7 and 30 days after transrectal prostate biopsy all participants have to fill in some questionnaires (sent by e-mail or post).

1. Questionnaire about the study outcome:

- presence of adverse events related to the antibiotics
- hospitalization, length of hospitalization, ICU admission, length of ICU admission
- - presence of infectious complications: UTI, pyelonephritis, fever, sepsis, severe sepsis or septic shock, acute prostatitis, acute epididymitis (for definitions of infectious complications see 6.1.1)
- antibiotic use after prostate biopsy (type and duration)
- compliance to antibiotic prophylaxis/correct use of antibiotic prophylaxis (only at day 7)
- travel to hospital and related costs due to infectious complications after prostate biopsy

1. EQ-5D-5L questionnaire (46): a validated heath-related quality of life instrument to assess patient’s health status for the purpose of the cost-effectiveness analysis of the culture-guided antibiotic prophylaxis strategy.
2. iMTA PC questionnaire (70): to assess productivity loss for the purpose of the cost-effectiveness analysis of the culture-guided antibiotic prophylaxis strategy.

*Microbiological characteristics:*

- Microbiological results of urine and blood cultures, including identification and full susceptibility testing results of bacteria cultured at the local microbiology laboratory within 30 days after prostate biopsy will be assessed using microbiological information systems.

Blood and urine cultures are only obtained in case of suspicion of infection. Patients are asked at the prostate biopsy visit to call/visit their treating urologist instead of their general practitioner or a different urologist in case of infection, to ensure all information and corresponding cultures are delivered to the study site. However, in case of referral to a hospital which is not a study site and with permission of the patient, medical records will be checked.

*Medical records:*

Medical records will be checked for the following information:

- compliance to antibiotic prophylaxis/correct use of antibiotic prophylaxis
- number of biopsy cores taken during transrectal prostate biopsy
- prostate volume on imaging
- biopsy results: presence of prostate malignancy, signs of histopathologic inflammation
- medical history: history of diabetes mellitus, liver disease, malignancy (solid tumors, leukaemia or malignant lymphoma), AIDS, chronic heart failure, COPD, dementia, hemiplegia, connective tissue disease, CVA or TIA, peripheral vascular disease, myocardial infarction or peptic ulcer disease (necessary to calculate the Charlson co-morbidity scoring system (67-69))
- renal function
- PSA level
- Gleason score
- TNM tumor staging
- hospitalization
- appointment(s) for infections
- new prescriptions of antibiotics
- microbiological results of urine and blood cultures
- missing data

**7.2 Analysis of determinants among healthcare professionals**

Similarly to the analysis of determinants among patients, after patient inclusion has ended, a study among healthcare professionals will be performed to assess barriers and facilitators that influence the performance of the culture-guided approach in daily clinical practice. Professionals involved in performing the culture-guided approach in the participating centers will be approached for an in-depth interview (approximately 3-5 professionals per center, for approximately 1 hour per professional). For a preliminary topic list, see Appendix 1. Based upon these results, a questionnaire will be developed to assess barriers and facilitators experienced among Dutch professionals.

**7.3 Randomization, blinding and treatment allocation**

Patients will be allocated to either the empiric prophylactic ciprofloxacin group or the intervention group. Randomization is performed after informed consent is obtained. Randomization will be 1:1 and stratified for the study site and type of transrectal prostate biopsy (random ultrasound-guided, targeted MRI-guided or targeted MRI-ultrasound fusion guided). A computer-generated randomization list will be produced by the study coordinator. Treatment allocation will be via a web based program (Castor EDC) which can be assessed 24 hours/day with secured log in (non-blinded). Authorized study staff will enter patient details into the system which will generate and record the allocation based on the randomization list. All transactions on the web server will be logged and auditable. Code of the randomization is kept by the study coordinator and will be broken only if necessary for safety reasons.

**7.4 Study procedures**

7.4.1 Rectal swabs and screening cultures

For the screening of resistant rectal flora, we developed a simple and rapid phenotypic culture method to guide the use of prophylactic antibiotics. Rectal swabs will be vortexed in transport medium (Eswab, Copan, Murrieta, CA) and 50 ul will be plated on four selective screening agars and a (growth) control Colombia blood agar (CBP) without additional antibiotics in the medical microbiology laboratory, depending on the hospital where the patient is treated.

Each of the four selective screening agars consists of a MacConkey medium with vancomycin, which is a commercially available selective and differential culture medium for bacteria designed to selectively isolate gram-negative bacilli, plus concentrations of the oral antibiotics used in this study:

1. Ciprofloxacin (0.5 mg/L)
2. Trimethoprim (2 mg/L)
3. Fosfomycine (4 mg/L) + glucose-6-phosphate (25 mg/L)
4. Mecillinam (2 mg/L) + amoxicillin/clavulanic acid (8 mg/L)

Agars will be incubated overnight at 37 °C. Growth on (one of) the selective agar plates demonstrates rectal carriage with GNB that have a higher MIC than the concentration in the agar, which means that the antibiotic in the agar cannot be prescribed as prophylaxis. The agar without growth (susceptible GNB to the antibiotic in the agar) is selected and the corresponding antibiotic is prescribed to the patient. Susceptibility of GNB for sulfamethoxazole is deduced from the trimethoprim agar.

When more than 1 agar shows no growth (thus more than one antibiotic option is available), the antibiotic is selected according to the decision-making flowchart *(Figure I)*. In case of growth on all 4 agars, a full susceptibility pattern of all morphologically diverse colonies on the ciprofloxacin agar is performed. The patient is prescribed ciprofloxacin in combination with one of the other oral study antibiotics. As last option, intravenous antibiotics (ceftriaxone, meropenem) will be prescribed based on susceptibility testing of isolates that grow on the control agar. Results of the rectum swab cultures and the corresponding selected antibiotic prophylaxis are reported to the research physician. Only culture results from patients in the intervention group will be used to target prophylaxis. Culture results of the control group will be saved in the study E-CRF but are not revealed before biopsy.

*Interpretation of culture results*

Study samples will be processed as standard clinical samples and all laboratory technicians of all microbiology laboratories will be trained to interpret results of the selective agars used for the study. A standard operating procedure (SOP) will be used. Because of the relative simple interpretation of growth on the agars (growth or no growth, and no susceptibility testing is needed), as well as the extensive experience with selective agars in general, no additional risks for misinterpretation of culture results are expected.

7.4.2 Blood and urine cultures post biopsy

Blood and urine cultures taken after prostate biopsy in case of (suspicion of) infection, will be transported to the microbiological laboratory of the study site and treated as routine cultures. Results will be communicated to the treating physician.

7.4.3 Storage of isolates

Culture isolates from faecal samples will be stored in the freezer at -70°C of the microbiological laboratory of the study site during the study period and be identifiable by study name, randomisation number, date and time of sampling and sampling code. For more information, see section 10.1 Handling and storage of data and documents.

**7.5 Withdrawal of individual subjects and follow-up of subjects withdrawn from treatment**

Subjects can leave the study at any time for any reason if they wish to do so without any consequences. The investigator can decide to withdraw a subject from the study for urgent medical reasons. If a patient is withdrawn prior to completion of the study, the reason for this decision will be recorded in the E-CRFs. The remaining follow-up, as part of standard care, will be conducted if patient consent is obtained.

**7.6 Replacement of individual subjects after withdrawal**

In total, 1618 adult men scheduled for transrectal prostate biopsy will be enrolled into the study (after meeting the inclusion criteria, passing the exclusion criteria and after written informed consent is obtained) for analysis of the primary outcome measure. All patients who do not need to undergo prostate biopsy (after inclusion), for example because of a normal MRI prostate, will be replaced. All other patients, who are lost to follow up for any other reason will not be replaced and included in analysis (per protocol analysis).

**7.7 Premature termination of the study**

In case the study is ended prematurely, the sponsor will notify the accredited METC and the competent authority within 15 days, including the reasons for the premature termination.

**8. SAFETY REPORTING**

**8.1 Temporary halt for reasons of subject safety**

In accordance to section 10, subsection 4, of the WMO, the sponsor will suspend the study if there is sufficient ground that continuation of the study will jeopardise subjects health or safety. The sponsor will notify the accredited METC without undue delay of a temporary halt including the reason for such an action. The study will be suspended pending a further positive decision by the accredited METC. The investigator will take care that all subjects are kept informed.

**8.2 AEs, SAEs and SUSARs**

8.2.1 Adverse events (AEs)

An adverse event is any symptom, sign, illness or undesirable experience, which develops or worsens in severity from informed consent to up to 30 days following the last administration of any study drugs.

These events include adverse drug reactions, illnesses with onset during the study, exacerbations of pre-existing illnesses, clinically significant changes in physical examination, or abnormal laboratory values. All adverse events reported spontaneously by the subject or observed by the investiga­tor or his staff will be recorded. For all adverse events, sufficient information should be obtained by the investigator to determine the causality of the event.

8.2.2 Serious adverse events (SAEs)

A serious adverse event is any untoward medical occurrence or effect that

results in death;

- is life threatening (at the time of the event);
- requires hospitalisation or prolongation of existing inpatients’ hospitalisation;
- results in persistent or significant disability or incapacity;
- is a congenital anomaly or birth defect; or
- any other important medical event that did not result in any of the outcomes listed above due to medical or surgical intervention but could have been based upon appropriate judgement by the investigator.

An elective hospital admission will not be considered as a serious adverse event.

All serious adverse events, whether or not considered to be related to the study treatment, will be reported to the sponsor without undue delay after obtaining knowledge of the events.

The sponsor will report the SAEs through the web portal *ToetsingOnline* to the accredited METC that approved the protocol, within 7 days of first knowledge for SAEs that result in death or are life threatening followed by a period of maximum of 8 days to complete the initial preliminary report. All other SAEs will be reported within a period of maximum 15 days after the sponsor has first knowledge of the serious adverse events.

8.2.3. Suspected unexpected serious adverse reactions (SUSARs)

Adverse reactions are all untoward and unintended responses to an investigational product related to any dose administered.

Unexpected adverse reactions are SUSARs if the following three conditions are met:

1. the event must be serious (see chapter 7.2.2);
2. there must be a certain degree of probability that the event is a harmful and an undesirable reaction to the medicinal product under investigation, regardless of the administered dose;
3. the adverse reaction must be unexpected, that is to say, the nature and severity of the adverse reaction are not in agreement with the product information as recorded in Summary of Product Characteristics (SPC).

The sponsor will report without delay all SUSARs through the web portal *ToetsingOnline* to the METC.

The expedited reporting of SUSARs through the web portal Eudravigilance or ToetsingOnline is sufficient as notification to the competent authority.

The expedited reporting will occur not later than 15 days after the sponsor has first knowledge of the adverse reactions. For fatal or life threatening cases the term will be maximal 7 days for a preliminary report with another 8 days for completion of the report.

**8.3 Annual safety report**

In addition to the expedited reporting of SUSARs, the sponsor will submit, once a year throughout the clinical trial, a safety report to the accredited METC and competent authority. This safety report consists of:

a list of all suspected (unexpected or expected) serious adverse reactions, along with an aggregated summary table of all reported serious adverse reactions.

**8.4 Follow-up of adverse events**

All AEs will be followed until they have abated, or until a stable situation has been reached. Depending on the event, follow up may require additional tests or medical procedures as indicated, and/or referral to the general physician or a medical specialist.

SAEs need to be reported till end of study within the Netherlands, as defined in the protocol .

**8.5 Data Safety Monitoring Board (DSMB)**

The risk-classification is assessed as negligible to the study participants. Therefore, there will be no Data Safety Monitoring Board in this study.

**9. STATISTICAL ANALYSIS**

Characteristics of the study population will be examined for overall study population, and for intervention and control groups, separately. Distribution of continuous data will be assessed with mean ± standard deviation in case of normal distribution and median (interquartile interval) in case of non-normal distribution. Distribution of categorical data will be assessed as number or percentages.

**9.1 Primary study parameter(s)**

The primary efficacy analysis is a comparative analysis regarding clinical complications 7 days after transrectal prostate biopsy on the intention-to-treat set based on a Cochrane-Mantel-Haenzel chi-square test stratified by institution. A 2-tailed P value of less than .05 will be used to indicate statistical significance. In case of fewer than 5 cases in a cell in 2 X 2 contingency table, we will perform Fisher’s Exact test by institution and will combine p-values using Fisher’s product method. Missing data for the primary endpoint are not expected in general because this data can be extracted from patient’s medical record. In case missing information is observed, we will carry last information forward. We consider patients who do not have infection to remain infection free and those who have infection will also have infection (i.e. considered as a failure) after 7 days, in both control and intervention group. Sub-analyses of the primary endpoint are performed in bacteremic/septic cases.

**9.2 Secondary study parameter(s)**

*Cost-effectiveness analysis*

Cost data will be skewed. This necessitates a method of analysis that can deal with skewed data. For cost data it is appropriate to use a method that employs a skewed distribution function such as a gamma. Therefore cost will be analyzed using a generalized linear model (GLM) using a log link and a gamma distribution. This method is also able to deal with covariates if necessary.

EQ-5D data are more likely to behave normal. Therefore we shall apply ordinary least squares (OLS) regression to these data and add if necessary covariates to the model.

The incremental cost-effectiveness ratio will be analyzed in a net monetary benefit framework. Depending on the distribution of the net monetary benefits an appropriate regression method (GLM or OLS) will be used and if necessary covariates will be added to the model.

In intention-to-treat analysis, positive microbiological urine or blood culture (within 7 and 30 days) and clinical infectious complications will be analyzed using Cochrane-Mantel-Haenzel chi-square test stratified by institution. Fisher’s Exact test by institution will be performed in case of fewer than 5 cases in a cell in 2 X 2 contingency table, and p-values will be combined using Fisher’s product method.

In case of missing data regarding secondary parameters, last information will be carried forward (e.g., no complications will be reported in missing cases).

**9.3 Other study parameters**

Similar to primary and secondary binary study parameters, in intention-to-treat analysis, other binary study parameters including hospitalization (within 30 days), overall mortality (within 30 days), side-effects and toxicity (within 30 days) occurring after transrectal prostate biopsy, will be analyzed using Cochrane-Mantel-Haenzel chi-square test stratified by institution. Similarly, in case of fewer than 5 cases in a cell in 2 X 2 contingency table, Fisher’s Exact test by institution will be performed and p-values will be combined using Fisher’s product method.

Continuous outcome including length of hospital stay and overall antibiotic use will be analyzed in a linear regression analyses while accounting for institution. In case data on continuous outcomes is skewed, analysis will be performed after log transforming the skewed data.

The second rectal Eswab will be stored. Susceptibility testing is performed only in case of infectious complications in an attempt to determine the cause of the infectious complication (see page 37).

Interviews on the experiences of patients and healthcare professionals will be recorded and transcribed verbatim by an independent transcriber. Two researchers will independently code the transcripts. A constant comparative method will be used for the analysis of the emerging themes form interviews, until saturation is reached i.e. no new themes are identified. Coding and analysis will be conducted by using a qualitative software program (Atlas.ti).

Based on these results, questionnaires will be developed for patients and for healthcare professionals. Descriptive statistics will be used to analyze these questionnaires.

Interim analysis

No interim analysis will be performed.

**Changes in the statistical analysis plan:**

**-** For the primary outcome measure, the 95% confidence interval for the difference in prevalence unstratified for hospitals was calculated.

- Since no method to adjust for multiple testing was specified in our statistical analysis plan and sample size calculation was only performed for the primary outcome measure, report of all secondary and exploratory endpoints were limited to point estimates of effects with 95% confidence intervals (unstratified for hospital) for the difference in those point estimates.

**10. ETHICAL CONSIDERATIONS**

**10.1 Regulation statement**

This study will be conducted in accordance with the ethical principles stated in the most recent version of the Declaration of Helsinki (7^th^ version, October 2013), the Medical Research Involving Human Subjects Act (WMO) and the applicable International Conference on Harmonisation (ICH) guidelines on Good Clinical Practice. The study will start after approval by the Ethics Review Board (CMO) region Arnhem – Nijmegen. Moreover, prior to initiation of the study, the study protocol and the informed consent form will have to be approved by the Institutional Review Boards.

**10.2 Recruitment and consent**

For a detailed description of the methods of patient recruitment and informed consent, see Chapter 3.

In short, an IEC-approved consent form for documenting written informed consent must be appropriately signed and dated by the subject or legal representative before participation in the study and prior to undertaking any rectal screening procedures. After reading the patient information letter and before the written informed consent form is signed, patients will have the possibility to ask questions about the study to the research team.

Patients will not sign the informed consent form in presence of the investigator. This is in the interests of the patients, namely to make logistics as easy as possible for them (no additional OPD visit) and to allow them sufficient time to consider the information. Moreover, we want to deviate as little as possible from daily clinical practice in order not to influence the barrier analysis. The investigator will sign the informed consent form after the patient has signed the form. A copy of the signed consent will be provided to the study participant. The signed consent will remain in each participants study file and be available for verification by study monitors at any time.

**10.3 Benefits and risks assessment, group relatedness**

Nature and extent of the burden associated with participation:

At three moments during the study period patients have to fill in a few questionnaires (see chapter 6).
At two moments during the study period a rectal swab is performed (at home by self-sampling approximately 14 days prior to prostate biopsy and immediately before prostate biopsy by the urologist). Utility of a rectum swab culture is a minimally invasive method to provide information on a participant’s rectal flora. It is already routine procedure for patients, admitted to Dutch hospitals, that are at risk for carriage of multidrug resistant bacteria (HRMO screening or methicillin-resistant Staphylococcus aureus (MRSA).(39) It must be mentioned that rectal swabs of the control group are taken and cultured but results are not used to guide prophylaxis.

According to the laws and regulations associated with research with a medicinal product, the study medication must be provided from the Clinical Trial Units of the hospital pharmacies of the participating centers. Therefore, participants can not obtain the antimicrobial prophylaxis at their home pharmacy. Participants will be offered two choices: to obtain the antimicrobial prophylaxis in the days before prostate biopsy at the Urology outpatient department (consequence: extra trip to the hospital) or to be present in the hospital two hours prior to prostate biopsy and then obtain the antimicrobial prophylaxis at the Urology outpatient department (and take the antimicrobial prophylaxis immediately). No extra other (study-related) hospital visits are required.

Risks associated with participation:

The risk-classification is assessed as negligible to the study participants.

Utility of a rectal Eswab is a common, safe and minimal invasive method to collect feces for research and diagnostics.

The alternative antibiotics which may be prescribed in case of FQ resistance are all registered in the Netherlands, used in clinical practice for the treatment of UTI and/or prostatitis and logical alternatives as antimicrobial prophylactic around prostate biopsy. We do not expect additional risks compared to the use of the standard antibiotic prophylaxis with ciprofloxacin and besides already registered adverse effects. In addition, it should be mentioned that it concerns antibiotic prophylaxis, which means that the antibiotics are used only for a short period (mostly only for up to 24 hours).

Benefit:
We expect that the intervention group (with rectal swab culture-guided antimicrobial prophylaxis) will have a lower risk of post-biopsy infectious complications compared to the control group (estimated at respectively 1.0% and 3.2%).

**10.4 Compensation for injury**

According to the Dutch law (WMO) the sponsor is obliged to have an insurance for compensation of subjects entered in clinical trials in the Netherlands who experienced trial related injury or death. Therefore, Radboudumc, The Netherlands, has arranged a liability insurance, which is detailed in the written subject information.

**11. ADMINISTRATIVE ASPECTS, MONITORING AND PUBLICATION**

**11.1 Handling and storage of data and documents**

The investigators will maintain adequate and accurate records to enable evaluation and reconstruction of the clinical trial and verification of the study data. These documents should be classified into 2 separate categories: 1) the investigator’s study file and 2) subject clinical source documents.

The investigator’s study file will contain the protocol/amendments, CRF and questionnaires, IEC, approval of the Medical Ethical Committee with correspondence, informed consent, drug records, staff curriculum vitae and authorizations forms, and other appropriate documents and correspondence.

Subject clinical source documents include, but are not limited to, the subject’s medical records, research case record forms, laboratory reports, ECG tracings, x-rays, radiologist’s reports, subject’s diaries and questionnaires, biopsy reports, ultrasound photographs, progress notes, pharmacy records, and any other similar reports or records of procedures performed during the subject’s participation in the study. The site investigators are responsible for maintaining any source documents related to the study. All clinical study documents will be retained by the investigator for at least 15 years.

All source data will be collected anonymously in a licensed electronic Case Report Form (E-CRF). The E-CRF is a secure online tool where the participating hospitals must login with an unique password. The E-CRF must be completed on an ongoing basis during the course of the study by authorized individuals. For each subject enrolled, the E-CRF must be reviewed and approved by the principal investigator or co-investigator within a reasonable time period after data collection. This also applies to records for those subjects who fail to complete the study (even during a pre-randomization screening period if a CRF was initiated). If a subject withdraws from the study, the reason must be noted on the CRF. In this study, Castor EDC will be used, which is a professional online data management system with a Good Clinical Practice certificate. Participating hospitals only have access to their own data. The key to the login code will be safeguarded by the study coordinator.

The investigator will assure that subjects’ anonymity will be strictly maintained and that their identities are protected form unauthorized parties. The medical record and other source documents are only accessible by the medical staff of the clinical research centre, the investigators and monitor as well as for audits by METC members or authorized government personnel. The CRFs will not contain identifiable information and will be coded with randomisation numbers only. Randomisation numbers will not be based on patients initials and birth-date. A subject identification code list will be used to link the data to the subject. The key to the code will be safeguarded by the study coordinator or principal investigator.

Culture isolates from faecal samples (Eswab 1) and faecal samples (Eswab 2) will be stored in the freezer at -70°C of the microbiological laboratory of the study site and be identifiable by study name, randomisation number, date of sampling and sampling code. Then, if necessary, certain tests can be performed again on the bacteria. Only investigators and laboratory staff will have access to the samples and data. The culture isolated will not be used in other studies. After the study participant has finished the study the material will be destroyed.

**11.2 Monitoring and Quality Assurance**

The conduct of this study will be monitored. The monitor will work independently and has no involvement in the set up of the study and interpretation of the results. The monitor is responsible for routine review of the CRFs at regular intervals throughout the study, to verify adherence to the protocol, and the completeness, consistency and accuracy of the data in the CRFs. The monitor will have access to any subject’s records needed to verify the entries on the CRFs. The investigators agree to cooperate with the monitor to ensure that any problems detected in the course of these monitoring visits are resolved.

Details about monitoring are stated in the monitoring plan.

**11.3 Amendments**

A ‘substantial amendment’ is defined as an amendment to the terms of the METC application, or to the protocol or any other supporting documentation, that is likely to affect to a significant degree:

- the safety or physical or mental integrity of the subjects of the trial;
- the scientific value of the trial;
- the conduct or management of the trial; or
- the quality or safety of any intervention used in the trial.

All substantial amendments will be notified to the METC and to the competent authority.

Non-substantial amendments will not be notified to the accredited METC and the competent authority, but will be recorded and filed by the sponsor.

**11.4 Annual progress report**

The sponsor/investigator will submit a summary of the progress of the trial to the accredited METC once a year. Information will be provided on the date of inclusion of the first subject, numbers of subjects included and numbers of subjects that have completed the trial, serious adverse events/serious adverse reactions, other problems, and amendments.

**11.5 Temporary halt and (prematurely) end of study report**

The sponsor will notify the accredited METC and the competent authority of the end of the study within a period of 90 days. The end of the study is defined as the last patient’s last visit.

The sponsor will notify the METC immediately of a temporary halt of the study, including the reason of such an action.

In case the study is ended prematurely, the sponsor will notify the accredited METC and the competent authority within 15 days, including the reasons for the premature termination.

Within one year after the end of the study, the investigator/sponsor will submit a final study report with the results of the study, including any publications/abstracts of the study, to the accredited METC and the Competent Authority.

**11.6**  **Public disclosure and publication policy**

The study was registered to a publicly accessible registry and results database (ClinicalTrials.gov). The investigator will submit any proposed publication or presentation along with the respective scientific journal or presentation forum at least 7 days prior to submission of the publication or presentation to all co-investigators.

**12. REFERENCES**

1. Volksgezondheidenzorg.info [updated 12th July 2017. RIVM Bilthoven:[Available from:

https://www.volksgezondheidenzorg.info/onderwerp/prostaatkanker/cijfers-context/huidige-situatie#!node-prevalentie-prostaatkanker.

2. Zani EL, Clark OA, Rodrigues Netto N, Jr. Antibiotic prophylaxis for transrectal prostate biopsy. Cochrane Database Syst Rev. 2011(5):CD006576.

3. Wolf JS, Jr., Bennett CJ, Dmochowski RR, Hollenbeck BK, Pearle MS, Schaeffer AJ, et al. Best practice policy statement on urologic surgery antimicrobial prophylaxis. J Urol. 2008;179(4):1379-90.

4. Grabe M, Bartoletti R, Bjerklund-Johansen TE, Çek HM, Pickard RS, Tenke P, et al. Guidelines on Urological Infections. European Association of Urology. 2013.

5. Urologie NVv. Richtlijn. Bacteriële urineweginfecties bij adolescenten en volwassenen. Etiologie, diagnostiek, behandeling en profylaxe. 2009.

6. Vlek ALM, Ruiter AEC, Vijverberg PLM, Kaan JA. Infectieuze complicaties en antibiotische profylaxe bij transrectale echogeleide prostaatbiopsie. Tijdschrift voor Urologie. 2014;5:119-24.

7. Aly M, Dyrdak R, Nordstrom T, Jalal S, Weibull CE, Giske CG, et al. Rapid increase in multidrug-resistant enteric bacilli blood stream infection after prostate biopsy - A 10-year population-based cohort study. Prostate. 2015;75(9):947-56.

8. Loeb S, Carter HB, Berndt SI, Ricker W, Schaeffer EM. Complications after prostate biopsy: data from SEER-Medicare. J Urol. 2011;186(5):1830-4.

9. Carignan A, Roussy JF, Lapointe V, Valiquette L, Sabbagh R, Pepin J. Increasing risk of infectious complications after transrectal ultrasound-guided prostate biopsies: time to reassess antimicrobial prophylaxis? Eur Urol. 2012;62(3):453-9.

10. Steensels D, Slabbaert K, De Wever L, Vermeersch P, Van Poppel H, Verhaegen J. Fluoroquinolone-resistant E. coli in intestinal flora of patients undergoing transrectal ultrasound-guided prostate biopsy--should we reassess our practices for antibiotic prophylaxis? Clin Microbiol Infect. 2012;18(6):575-81.

11. Liss MA, Taylor SA, Batura D, Steensels D, Chayakulkeeree M, Soenens C, et al. Fluoroquinolone resistant rectal colonization predicts risk of infectious complications after transrectal prostate biopsy. J Urol. 2014;192(6):1673-8.

12. Loeb S, van den Heuvel S, Zhu X, Bangma CH, Schroder FH, Roobol MJ. Infectious complications and hospital admissions after prostate biopsy in a European randomized trial. Eur Urol. 2012;61(6):1110-4.

13. Summers SJ, Patel DP, Hamilton BD, Presson AP, Fisher MA, Lowrance WT, et al. An antimicrobial prophylaxis protocol using rectal swab cultures for transrectal prostate biopsy. World J Urol. 2015;33(12):2001-7.

14. Marino K, Parlee A, Orlando R, Lerner L, Strymish J, Gupta K. Comparative Effectiveness of Single versus Combination Antibiotic Prophylaxis for Infections after Transrectal Prostate Biopsy. Antimicrob Agents Chemother. 2015;59(12):7273-5.

15. Taylor S, Margolick J, Abughosh Z, Goldenberg SL, Lange D, Bowie WR, et al. Ciprofloxacin resistance in the faecal carriage of patients undergoing transrectal ultrasound guided prostate biopsy. BJU Int. 2013;111(6):946-53.

16. Zaytoun OM, Vargo EH, Rajan R, Berglund R, Gordon S, Jones JS. Emergence of fluoroquinolone-resistant Escherichia coli as cause of postprostate biopsy infection: implications for prophylaxis and treatment. Urology. 2011;77(5):1035-41.

17. Abughosh Z, Margolick J, Goldenberg SL, Taylor SA, Afshar K, Bell R, et al. A prospective randomized trial of povidone-iodine prophylactic cleansing of the rectum before transrectal ultrasound guided prostate biopsy. J Urol. 2013;189(4):1326-31.

18. Anderson E, Leahy O, Cheng AC, Grummet J. Risk factors for infection following prostate biopsy - a case control study. BMC Infect Dis. 2015;15:580.

19. Antsupova V, Norgaard N, Bisbjerg R, Nygaard Jensen J, Boel J, Jarlov JO, et al. Antibiotic prophylaxis for transrectal prostate biopsy-a new strategy. J Antimicrob Chemother. 2014;69(12):3372-8.

20. Campeggi A, Ouzaid I, Xylinas E, Lesprit P, Hoznek A, Vordos D, et al. Acute bacterial prostatitis after transrectal ultrasound-guided prostate biopsy: epidemiological, bacteria and treatment patterns from a 4-year prospective study. Int J Urol. 2014;21(2):152-5.

21. Greeff de SC, Mouton JW. NethMap 2017: Consumption of antimicrobial agents and antimicrobial resistance among medically important bacteria in the Netherlands / MARAN 2017: Monitoring of antimicrobial resistance and antibiotic usage in animals in the Netherlands in 2016. RIVM Bilthoven; 2017.

22. Verburgh HA, Neeling de H. NETHMAP 2003: Consumption of antimicrobial agents and antimicrobial resistance among medically important bacteria in the Netherlands. RIVM Bilthoven; 2003.

23. Dai J, Leone A, Mermel L, Hwang K, Pareek G, Schiff S, et al. Rectal swab culture-directed antimicrobial prophylaxis for prostate biopsy and risk of postprocedure infection: a cohort study. Urology. 2015;85(1):8-14.

24. Duplessis CA, Bavaro M, Simons MP, Marguet C, Santomauro M, Auge B, et al. Rectal cultures before transrectal ultrasound-guided prostate biopsy reduce post-prostatic biopsy infection rates. Urology. 2012;79(3):556-61.

25. Farrell JJ, Hicks JL, Wallace SE, Seftel AD. Impact of preoperative screening for rectal colonization with fluoroquinolone-resistant enteric bacteria on the incidence of sepsis following transrectal ultrasound guided prostate biopsy. Res Rep Urol. 2017;9:37-41.

26. Van Besien J, Uvin P, Van den Abeele AM, Merckx L. Prevalence, Risk Factors, and Clinical Relevance of Fluoroquinolone-Resistant Organisms in Rectal Cultures: Should We Target Antibiotic Prophylaxis Prior to Prostate Biopsy? Adv Urol. 2016;2016:5392107.

27. Mottet N, Bellmunt J, Briers E, Bergh van den RCN, Bolla M, Casteren van NJ, et al. Guidelines on prostate cancer. European Association of Urology; 2015.

28. Cussans A, Somani BK, Basarab A, Dudderidge TJ. The role of targeted prophylactic antimicrobial therapy before transrectal ultrasonography-guided prostate biopsy in reducing infection rates: a systematic review. BJU Int. 2016;117(5):725-31.

29. Lu DD, Raman JD. Strategies for prevention of ultrasound-guided prostate biopsy infections. Infect Drug Resist. 2016;9:161-9.

30. Johnson DC, Reiter RE. Multi-parametric magnetic resonance imaging as a management decision tool. Transl Androl Urol. 2017;6(3):472-82.

31. Futterer JJ, Briganti A, De Visschere P, Emberton M, Giannarini G, Kirkham A, et al. Can Clinically Significant Prostate Cancer Be Detected with Multiparametric Magnetic Resonance Imaging? A Systematic Review of the Literature. Eur Urol. 2015;68(6):1045-53.

32. Quentin M, Blondin D, Arsov C, Schimmoller L, Hiester A, Godehardt E, et al. Prospective evaluation of magnetic resonance imaging guided in-bore prostate biopsy versus systematic transrectal ultrasound guided prostate biopsy in biopsy naive men with elevated prostate specific antigen. J Urol. 2014;192(5):1374-9.

33. Li CK, Tong BC, You JH. Cost-effectiveness of culture-guided antimicrobial prophylaxis for the prevention of infections after prostate biopsy. Int J Infect Dis. 2016;43:7-12.

34. Taylor AK, Zembower TR, Nadler RB, Scheetz MH, Cashy JP, Bowen D, et al. Targeted antimicrobial prophylaxis using rectal swab cultures in men undergoing transrectal ultrasound guided prostate biopsy is associated with reduced incidence of postoperative infectious complications and cost of care. J Urol. 2012;187(4):1275-9.

35. Cook I, Angel JB, Vera PL, Demos J, Preston D. Rectal swab testing before prostate biopsy: experience in a VA Medical Center urology practice. Prostate Cancer Prostatic Dis. 2015;18(4):365-9.

36. Liss MA, Kim W, Moskowitz D, Szabo RJ. Comparative Effectiveness of Targeted vs Empirical Antibiotic Prophylaxis to Prevent Sepsis from Transrectal Prostate Biopsy: A Retrospective Analysis. J Urol. 2015;194(2):397-402.

37. Hillelsohn JH, Duty B, Blute ML, Jr., Okhunov Z, Kashan M, Moldwin R, et al. Variability of transrectal ultrasound-guided prostate biopsy prophylactic measures. Can J Urol. 2012;19(6):6573-7.

38. Redgrave LS, Sutton SB, Webber MA, Piddock LJ. Fluoroquinolone resistance: mechanisms, impact on bacteria, and role in evolutionary success. Trends Microbiol. 2014;22(8):438-45.

39. NVMM Guideline Laboratory detection of highly resistant microorganisms. 2011 (version 1.0).

40. El-Halfawy OM, Valvano MA. Antimicrobial heteroresistance: an emerging field in need of clarity. Clin Microbiol Rev. 2015;28(1):191-207.

41. Lunny C, Taylor D, Hoang L, Wong T, Gilbert M, Lester R, et al. Self-Collected versus Clinician-Collected Sampling for Chlamydia and Gonorrhea Screening: A Systemic Review and Meta-Analysis. PLoS One. 2015;10(7):e0132776.

42. Snijders PJ, Verhoef VM, Arbyn M, Ogilvie G, Minozzi S, Banzi R, et al. High-risk HPV testing on self-sampled versus clinician-collected specimens: a review on the clinical accuracy and impact on population attendance in cervical cancer screening. Int J Cancer. 2013;132(10):2223-36.

43. Liss MA, Nakamura KK, Meuleners R, Kolla SB, Dash A, Peterson EM. Screening rectal culture to identify fluoroquinolone-resistant organisms before transrectal prostate biopsy: do the culture results between office visit and biopsy correlate? Urology. 2013;82(1):67-71.

44. Ijzerman MJ. Richtlijn voor het uitvoeren van economische evaluaties in de gezondheidszorg. 2016.

45. Bouwmans C, Krol M, Severens H, Koopmanschap M, Brouwer W, Hakkaart-van Roijen L. The iMTA Productivity Cost Questionnaire: A Standardized Instrument for Measuring and Valuing Health-Related Productivity Losses. Value Health. 2015;18(6):753-8.

46. Versteegh M. Impact on the Incremental Cost-Effectiveness Ratio of Using Alternatives to EQ-5D in a Markov Model for Multiple Sclerosis. Pharmacoeconomics. 2016;34(11):1133-44.

47. Fleuren MA, Paulussen TG, Van Dommelen P, Van Buuren S. Towards a measurement instrument for determinants of innovations. Int J Qual Health Care. 2014;26(5):501-10.

48. Flottorp SA, Oxman AD, Krause J, Musila NR, Wensing M, Godycki-Cwirko M, et al. A checklist for identifying determinants of practice: a systematic review and synthesis of frameworks and taxonomies of factors that prevent or enable improvements in healthcare professional practice. Implement Sci. 2013;8:35.

49. Charalabopoulos K, Karachalios G, Baltogiannis D, Charalabopoulos A, Giannakopoulos X, Sofikitis N. Penetration of antimicrobial agents into the prostate. Chemotherapy. 2003;49(6):269-79.

50. Jeppesen N, Frimodt-Moller C. Serum concentrations and penetration into prostate of mecillinam and ampicillin. Curr Med Res Opin. 1984;9(3):213-8.

51. Rhodes NJ, Gardiner BJ, Neely MN, Grayson ML, Ellis AG, Lawrentschuk N, et al. Optimal timing of oral fosfomycin administration for pre-prostate biopsy prophylaxis. J Antimicrob Chemother. 2015;70(7):2068-73.

52. Gardiner BJ, Mahony AA, Ellis AG, Lawrentschuk N, Bolton DM, Zeglinski PT, et al. Is fosfomycin a potential treatment alternative for multidrug-resistant gram-negative prostatitis? Clin Infect Dis. 2014;58(4):e101

53. Aron M, Rajeev TP, Gupta NP. Antibiotic prophylaxis for transrectal needle biopsy of the prostate: a randomized controlled study. BJU Int. 2000;85(6):682-5.

54. Kapoor DA, Klimberg IW, Malek GH, Wegenke JD, Cox CE, Patterson AL, et al. Single-dose oral ciprofloxacin versus placebo for prophylaxis during transrectal prostate biopsy. Urology. 1998;52(4):552-8.

55. Isen K, Kupeli B, Sinik Z, Sozen S, Bozkirli I. Antibiotic prophylaxis for transrectal biopsy of the prostate: a prospective randomized study of the prophylactic use of single dose oral fluoroquinolone versus trimethoprim-sulfamethoxazole. Int Urol Nephrol. 1999;31(4):491-5.

56. Hills NH, Bultitude MI, Eykyn S. Co-trimoxazole in prevention of bacteriuria after prostatectomy. Br Med J. 1976;2(6034):498-9.

57. Cai T, Gallelli L, Cocci A, Tiscione D, Verze P, Lanciotti M, et al. Antimicrobial prophylaxis for transrectal ultrasound-guided prostate biopsy: fosfomycin trometamol, an attractive alternative. World J Urol. 2017;35(2):221-8.

58. Lista F, Redondo C, Meilan E, Garcia-Tello A, Ramon de Fata F, Angulo JC. Efficacy and safety of fosfomycin-trometamol in the prophylaxis for transrectal prostate biopsy. Prospective randomized comparison with ciprofloxacin. Actas Urol Esp. 2014;38(6):391-6.

59. Fahmy AM, Kotb A, Youssif TA, Abdeldiam H, Algebaly O, Elabbady A. Fosfomycin antimicrobial prophylaxis for transrectal ultrasound-guided biopsy of the prostate: A prospective randomised study. Arab J Urol. 2016;14(3):228-33.

60. Sen V, Aydogdu O, Bozkurt IH, Yonguc T, Sen P, Polat S, et al. The use of prophylactic single-dose fosfomycin in patients who undergo transrectal ultrasound-guided prostate biopsy: A prospective, randomized, and controlled clinical study. Can Urol Assoc J. 2015;9(11-12):E863-7.

61. Ongun S, Aslan G, Avkan-Oguz V. The effectiveness of single-dose fosfomycin as antimicrobial prophylaxis for patients undergoing transrectal ultrasound-guided biopsy of the prostate. Urol Int. 2012;89(4):439-44.

62. Kisa E, Altug MU, Gurbuz OA, Ozdemir H. Fosfomycin: a good alternative drug for prostate biopsy prophylaxis the results of a prospective, randomized trial with respect to risk factors. Int Braz J Urol. 2017;43.

63. Christoffersen T, Bjerrum L, Nielsen AB. General practitioners do not systematically adhere to regional recommendations on treatment of uncomplicated urinary tract infections. Dan Med J. 2014;61(4):A4814.

64. Singer M, Deutschman CS, Seymour CW, Shankar-Hari M, Annane D, Bauer M, et al. The Third International Consensus Definitions for Sepsis and Septic Shock (Sepsis-3). JAMA. 2016;315(8):801-10.

65. Fujimura T, Kume H, Nishimatsu H, Sugihara T, Nomiya A, Tsurumaki Y, et al. Assessment of lower urinary tract symptoms in men by international prostate symptom score and core lower urinary tract symptom score. BJU Int. 2012;109(10):1512-6.

66. Barry MJ, Fowler FJ, Jr., O'Leary MP, Bruskewitz RC, Holtgrewe HL, Mebust WK, et al. The American Urological Association symptom index for benign prostatic hyperplasia. The Measurement Committee of the American Urological Association. J Urol. 1992;148(5):1549-57; discussion 64.

67. Charlson ME, Pompei P, Ales KL, MacKenzie CR. A new method of classifying prognostic comorbidity in longitudinal studies: development and validation. J Chronic Dis. 1987;40(5):373-83.

68. Sundararajan V, Henderson T, Perry C, Muggivan A, Quan H, Ghali WA. New ICD-10 version of the Charlson comorbidity index predicted in-hospital mortality. J Clin Epidemiol. 2004;57(12):1288-94.

69. Quan H, Li B, Couris CM, Fushimi K, Graham P, Hider P, et al. Updating and validating the Charlson comorbidity index and score for risk adjustment in hospital discharge abstracts using data from 6 countries. Am J Epidemiol. 2011;173(6):676-82.

70. Bouwmans C, Krol M, Brouwer W, Severens JL, Koopmanschap MA, Hakkaart L. IMTA Productivity Cost Questionnaire (IPCQ). Value Health. 2014;17(7):A550.

**13. AMENDMENTS TO THE PROTOCOL**

Amendments to the original protocol have been marked in the last version of our protocol (yellow) and are summarized below:

- Initially, we planned to conduct the study in three Dutch hospitals. Due to changes in the daily clinical practice around prostate biopsy in some hospitals, the corona pandemic and delay in the start of the clinical trial, the recruitment rate was lower than expected (as stated on page 33 of the protocol, version 11.0, 4 August 2020). Therefore, the study was extended to eleven participating hospitals. These hospitals started with the recruitment of patients between December 2018 and August 2020. Approval for recruitment in additional hospitals was obtained by the Medical Research Ethics Committee and the concerning institutional review boards. As a result, rectal culture tests were performed in six clinical microbiological laboratories connected to the participating hospitals in our trial instead of two clinical microbiological laboratories in which we initially planned to perform these tests.
- In March 2019, the European Medicine Agency finalized a review of serious, disabling side effects with fluoroquinolone antibiotics. Exclusion criteria were added to the study protocol to avoid the use of fluoroquinolones in patients at high risk for these side effects, see page 13 of the study protocol, version 11. Moreover, re-biopsy within seven days was added as exclusion criteria. Repeated biopsy within seven days makes it impossible to reliably assess the primary outcome measure: it was not foreseen in the original protocol that this could occur.
- In our grant proposal and preliminary versions of the study protocol, we made a power calculation (estimated at 1332 patients) based on the assumption that all patient underwent prostate biopsy with systematic biopsies. However, between the writing of our protocol and approval of the trial by the Medical Research Ethics Committee, the daily clinical practice around prostate biopsy changed at the Radboudumc site: systematic ultrasound-guided prostate biopsy was fully replaced by MRI-guided or MRI-ultrasound fusion guided prostate biopsy. The newly introduced MRI-based techniques allowed urologists to more accurate target biopsies with only 2 till 4 core samples (instead of 12 core samples). Because less core samples were taken with these techniques, we hypothesized that the amount of infectious complications after MRI-guided or MRI-ultrasound fusion guided prostate biopsies would be reduced compared systematic ultrasound-guided prostate biopsies (see page 7 and 27 of the original protocol). For this reason, we adapted the power calculation in our original protocol under the assumption that we would include at least 200 patients at the Radboudumc site. Because the recruitment rate at the Radboudumc was considerably lower than expected (30 patients included instead of the expected 200 patients), we decided in August 2020 (after consultation with the statistician) to go back to the first power calculation (of the grant proposal and preliminary versions of the study protocol) for both financial and ethical reasons (see page 32-33 of the final protocol).

**Changes in the statistical analysis plan:**

*see page 49 of the final protocol*

- For the primary outcome measure, the 95% confidence interval for the difference in prevalence unstratified for hospitals was calculated.
- Since no method to adjust for multiple testing was specified in our statistical analysis plan and sample size calculation was only performed for the primary outcome measure, report of all secondary and exploratory endpoints were limited to point estimates of effects with 95% confidence intervals (unstratified for hospital) for the difference in those point estimates.

N.B. The presented study is part of a larger project including three research questions, as reflected in our protocol (implementation, effectiveness and cost-effectiveness of culture-based antibiotic prophylaxis in transrectal prostate biopsy). For pragmatic reasons, all three research questions were included in a single protocol and submitted for approval to the Medical Ethics Committee. Research on the implementation of culture-based prophylaxis is already finished and results have been published (PMID: 34806004). Data-analysis on the cost-effectiveness of culture-based prophylaxis is still ongoing and is outside the scope of this paper.

**Supplementary File II: Rectal culture protocol**

Collected swabs were transported to one of six clinical microbiological laboratories connected to participating hospitals. The rectal swab (Eswab, Copan Diagnostics, Murrietta, CA, USA) was inoculated on four different MacConkey agars. Each MacConkey agar contained 20 mg/l vancomycin (to inhibit the growth of gram-positive bacteria) and one of the following antibiotics (MediaproductsBV, Groningen, The Netherlands): ^1^

1. 0.5 mg/l ciprofloxacin

2. 2 mg/l trimethoprim

3. 4 mg/l fosfomycin and 25 mg/L glucose-6-phosphate

(for maximal enhancement of fosfomycin activity in *in vitro* testing)

4. 2 mg/l mecillinam and 8 mg/l amoxicillin/clavulanic acid

Trimethoprim was used as marker for trimethoprim/sulfamethoxazole resistance. Mecillinam was used instead of pivmecillinam, since pivmecillinam is a prodrug and therefore not active in vitro. Mecillinam was combined with amoxicillin/clavulanic acid, because this combination was found to synergistically reduce the mean inhibitory concentration (MIC) of mecillinam over 32-fold in extended-spectrum-β-lactamase-producing *Escherichia coli*.^2^ Agar concentrations for ciprofloxacin, trimethoprim and amoxicillin/clavulanic acid are clinical breakpoint concentrations indicating resistance (R) for Enterobacterales and Pseudomonas as recommended by EUCAST.^3^ For fosfomycine and mecillinam, lower agar concentrations than EUCAST breakpoint were used, as these breakpoints are for uncomplicated urinary tract infections only and do not take into account drug concentrations in the prostate.

The swab was also inoculated on a Columbia agar supplemented with 5% sheep blood (Becton Dickinson GmbH, Heidelberg, Germany) to ensure that the swab was taken correctly. After 24 and 48 hours of incubation at 35°C under aerobic conditions all inoculated agars were assessed for growth. If there was no growth on MacConkey agar(s) (and growth on the control agar) after the 48-hour incubation, the rectal flora was assumed to be sensitive for the antibiotic within the agar. Vice versa, any growth of Enterobacterales, *Acinetobacter* species or *Pseudomonas aeruginosa* on MacConkey agar(s) was assumed to be resistant for the antibiotic within the agar. Growth of other bacteria was not taken into account. The identification of bacteria was performed by analyzing each colony with different morphology of each separate MacConkey agar with matrix-assisted laser desorption ionization-time of flight mass spectrometry (MALDI-TOF MS, Bioptyper version 3, Bruker, Bremen, Germany).

In case of growth on all four different MacConkey agars, for each colony with different morphology isolated from the MacConkey agar with ciprofloxacin the MIC was determined for trimethoprim, fosfomycin, amoxicillin/clavulanic acid and mecillinam using a MIC test strip (MTS, Liofilchem, Italy) on a Mueller-Hinton agar (with glucose-6-phophate for fosfomycin) (Oxoid, ThermoFisher, Waltham, USA) (to be able to combine two oral antibiotics as prophylaxis: ciprofloxacin and another oral antibiotic). If necessary, an automated identification and susceptibility testing system (VITEK®2 or BD Phoenix^TM^) was used to perform susceptibility testing for intravenous antibiotics.

Immediately prior to prostate biopsy, a second rectal swab was collected by the healthcare provider. This swab was inoculated on a MacConkey agar containing 20 mg/l vancomycin. After 48-hour incubation at 35°C under aerobic conditions, all Enterobacteriaceae, *Acinetobacter* species and *Pseudomonas aeruginosa* colonies with different morphology were stored in the freezer at -20°C. In case of an infectious complication, on these isolates, susceptibility testing for ciprofloxacin and the antibiotic(s) prescribed as prophylaxis was performed using a MIC test strip (MTS, Liofilchem, Italy) on a Mueller-Hinton agar (Oxoid, ThermoFisher, Waltham, USA).

**REFERENCES**

1. Tops SCM, Bruens M, van Mook-Vermulst S, et al. Performance Validation of Selective Screening Agars for Guiding Antimicrobial Prophylaxis in Patients Undergoing Prostate Biopsy. J Clin Microbiol 2018;56.

2. Lampri N, Galani I, Poulakou G, et al. Mecillinam/clavulanate combination: a possible option for the treatment of community-acquired uncomplicated urinary tract infections caused by extended-spectrum beta-lactamase-producing Escherichia coli. J Antimicrob Chemother 2012;67:2424-8.

3. European Committee on antimicrobial susceptibility testing (EUCAST) 2018 [Available from: http://www.eucast.org/clinical_breakpoints/.

**Supplementary File III: Patient Questionnaires**

Questionnaire 1 (sent before prostate biopsy):

**Explanation: please read this first!**

**What is the questionnaire about?**

This questionnaire is part of the medical research you participate in regarding culture-based prophylaxis in transrectal prostate biopsy. This questionnaire is about your medical history, urinary symptoms, medication use and state of health. First we will ask you some general questions, for example your weight and height.

**How long does it take to complete the questionnaire?**

It will take approximately 30 minutes to complete the questionnaire.

**How to fill in the questionnaire?**

- This is the first questionnaire. In the coming weeks you will receive two more (shorter) questionnaires. It is important to fill in the questionnaire completely.
- Start with the first question and follow the numbering.
- Tick one box for each question, unless stated else.
- For some questions, you can enter a number or something else on the dotted line.
- Fill in the questions based on your experience and opinion. There are no right or wrong answers here.


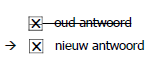
**Do you want to change an answer?**

- Cross out the old answer
- Tick a new answer
- Place an arrow in front of the new answer

**Can’t fill in the questionnaire yourself?**

If you can’t complete the list yourself, someone may be able to help you. For example, an family member.

**What happens with your answers?**

Your answers will be used for research. Only the researchers will see your answers, no one else. The investigators will not tell anyone that you participated in the trial.

**Thanks you for filling in the list for us!**

**General questions**

**Research number:**

**Question 1. On which date did you complete this list?**

**Questions 2. What is your age?**

years

**Question 3. Wat is your length?**

centimeter

**Question 4. What is your weight?**

If you do not know your weight, try to estimate it as accurate as possible

kilogram

**Question 5. Do you smoke?**

No, never

Yes, namely

On average more than 30 cigarettes, shags or cigars per day

On average 20-30 cigarettes, shags or cigars per day

On average 10-20 cigarettes, shags or cigars per day

On average 5-10 cigarettes, shags or cigars per day

On average less than 5 cigarettes, shags or cigars per day

*(If you have indicated that you smoke, please continue at question 7a)*

*(If you have indicated that you do not smoke, please continue at question 6)*

**Question 6. Did you smoke in the past?**

No

Yes, namely

On average more than 30 cigarettes, shags or cigars per day

On average 20-30 cigarettes, shags or cigars per day

On average 10-20 cigarettes, shags or cigars per day

On average 5-10 cigarettes, shags or cigars per day

On average less than 5 cigarettes, shags or cigars per day

**Question 7a. Have you been abroad in the past six months?**

No *(if no, please continue at question 8)*

Yes *(if yes, please continue at question 7b)*

**Question 7b. Which countries have you visited in the past six months?**

………………………………………………………………………………………………………………………………………………………………………………………………………………………………………………………………………………………………………………………………………………………………………

**Question 8. Do you work in healthcare?**

No

Yes

**The following questions are about medication use and allergies**

**Question 9. What is the name of your pharmacy?**

Name:……………………………………………………………………………………………………………..

Place:……………………………………………………………………………………………………………..

**Question 10. Do you use any medication?**

No

Yes

**Question 11. Did you use any antibiotics in the past 14 days?**

No

Yes

**Question 12a. Did you use any antibiotics in the past year?**

No *(if no, please continue at question 13)*

Yes *(if yes, please continue at question 12b)*

**Question 12b. Which antibiotics did you use in the past year? (multiple answers possible)**

Amoxicillin Pheneticillin (Broxil)

Amoxicillin//clavulanic acid (Augmentin) Flucloxacillin (Floxapen)

Azithromycin (Zitromax) Fosfomycin (Monuril)

Ciprofloxacin (Ciproxin) Moxifloxacin (Avelox)

Clindamycin (Dalacin) Metronidazole (Flagyl)

Clarythromycin (Klacid) Nitrofurantoin (Furabid)

Doxycycline Trimethoprim/sulfamethoxazole

Intravenous antibiotics

Otherwise, namely………………………………………………………………………………………………

I do not know the name of the antibiotic(s)

**Question 12c. What was the indication for antibiotic use?**

........................................................................................................................................................................................

I do not know the indication for antibiotic use (anymore)?

**Question 13. Do you have a history of allergic reaction to antibiotics?**

No

Yes, namely (multiple answers possible)

Itching and/or rash Swelling and/or shortness of breath

Nausea, vomiting and/or diarrhea Otherwise, namely ……………………….........

I do not know

**The following questions are about your medical history**

**Question 14. Did you have an infection of the bladder, urinary tract or prostate in the past two weeks?**

No

Yes

**Question 15. Did you have an examination or procedure via the uretha in the past 30 days?**

No

Yes, namely …………………………………………………………………………………………………..

I do not know

**Question 16a. Have you ever had a prostate biopsy before?**

No *(if no, please continue at question 17)*

Yes *(if yes, please continue at question 16b)*

I do not know *(if you do not know, please continue at question 17)*

**Question 16b. When did you have the previous prostate biopsy?**

If you don't remember exactly, please enter an approximate date (if possible)

Date: (day – month – year)

I do not know (anymore)

**Question 16c. Did you use antibiotics around this previous prostate biopsy prescribed by your urologist to prevent infections (prophylaxis)?**

No

Yes

I do not know

**Question 17. Do you have an indwelling catheter or perform intermittent catheterization?**

No

Yes, namely

I have an indwelling catheter

I perform intermittent catheterization

**Question 18a. Have you been hospitalized in the past year?**

No *(if no, please continue at question 19)*

Yes *(if yes, please continue at question 18b)*

**Question 18b. What was the date and duration of hospitalization in the past year?**

If you don’t remember exactly, please enter an approximate date and duration (if possible)

Date: (day – month – year)

Duration: days

I do not know (anymore)

**Question 19a. Have you - currently or in the past - been treated by a medical specialist in another hospital than where you are undergoing the prostate biopsy?**

No *(if no, please continue at question 20)*

Yes, namely ………………………………………………………*(if yes, please continue at question 19b)*

**Question 19b. For which complaint(s) or condition(s) have you currently or in the past been treated by a medical specialist in another hospital than where you are undergoing the prostate biopsy?**

………………………………………………………………………………………………………………………………………………………………………………………………………………………………………………………………………………………………………………………………………………………………………

**The following questions are used to estimate how many urinary complaints you have.**

**Please tick the answer that applies to you.**

| **Symptoms** | **Not at all** | **Less than 1 time in 5** | **Less than half the time** | **About half the time** | **More than half the time** | **Almost always** |
| --- | --- | --- | --- | --- | --- | --- |
| Over the past month, how often have you had a sensation of not emptying your bladder completely after you finish urinating? |  |  |  |  |  |  |
| Over the past month, how often have you had to urinate again less than two hours after you finished urinating? |  |  |  |  |  |  |
| Over the past month, how often have you found you stopped and started again several times when you urinated? |  |  |  |  |  |  |
| Over the last month, how difficult have you found it to postpone urination? |  |  |  |  |  |  |
| Over the past month, how often have you had a weak urinary stream? |  |  |  |  |  |  |
| Over the past month, how often have you had to push or strain to begin urination? |  |  |  |  |  |  |

| **Symptoms** | **None** | **1 time** | **2 times** | **3 times** | **4 times** | **5 times or more** |
| --- | --- | --- | --- | --- | --- | --- |
| Over the past month, many times did you most typically get up to urinate from the time you went to bed until the time you  got up in the morning? |  |  |  |  |  |  |

| **Quality of life due to urinary symptoms** | **Delig-thed** | **Pleased** | **Mostly satisfied** | **Mixed: equally statis-fied/ dissatis-fied** | **Un-happy** | **Terrible** |
| --- | --- | --- | --- | --- | --- | --- |
| If you were to spend the rest of your life with your urinary condition the way it is now, how would you feel about that? |  |  |  |  |  |  |

**This was the last question.**laatste vraag.

Do you have any questions or comments? Please let us know in the comments section below.

……………………………………………………………………………………………………………………....................................................................................................................................................................................................................................................................................................................................................................................................................................................................................................................................................................

Questionnaire 2 (sent approximately seven days post-biopsy)

**Explanation: please read this first!**

**What is the questionnaire about?**

This questionnaire is part of the medical research you participate in regarding culture-based prophylaxis in transrectal prostate biopsy. This questionnaire is about the use of antibiotics and the occurrence of infections after the prostate biopsy.

**How long does it take to complete the questionnaire?**

It will take approximately 15 minutes to complete the questionnaire.

**How to fill in the questionnaire?**

- You will receive three questionnaires. This is the second questionnaire. It is important to fill in the questionnaire completely. Even if you have already answered the question in the previous list.
- Start with the first question and follow the numbering.
- Tick one box for each question, unless stated else.
- For some questions, you can enter a number or something else on the dotted line.
- Fill in the questions based on your experience and opinion. There are no right or wrong answers here.


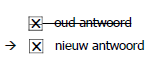
**Do you want to change an answer?**

- Cross out the old answer
- Tick a new answer
- Place an arrow in front of the new answer

**Can’t fill in the questionnaire yourself?**

If you can’t complete the list yourself, someone may be able to help you. For example, an family member.

**What happens with your answers?**

Your answers will be used for research. Only the researchers will see your answers, no one else. The investigators will not tell anyone that you participated in the trial.

**Thanks you for filling in the list for us!**

**The following questions are about the use of antibiotics, the occurrence of infections and hospitalization in the period from prostate biopsy until now.**

**Research number:**

**Question 1. On which date did you complete this questionnaire?**

**Question 2. For the reliability of the trial, it is important for us to know whether you have used the antibiotics according to the instructions.**

These are the antibiotics that we have prescribed to prevent an infection after prostate biopsy (prophylaxis). If you have not used these antibiotics according to the instructions, this will not affect your participation in the trial.

No, I did not use the antibiotics according to the instructions, because:

I did not start the antibiotics two hours before prostate biopsy, but …………………………………

I have not taken all the tablets. Explanation:…………………………………………………..………

I did not use the antibiotics (at all)

Otherwise, namely

Yes, I did use the antibiotics according to the instructions.

*If you did not use the antibiotics at all, please continue at question 4. In all other cases, please continue at question 3.*

**We would appreciate it if you could return the form *Antibiotic use* with this list.**

**Question 3. Have you experienced side effects/complaints related to the antibiotics?**

It concerns the antibiotics that we have prescribed to prevent an infection after prostate biopsy (prophylaxis).

No

Yes, namely…………………………………………………………………………………………………

**Question 4. Recently you underwent a prostate biopsy.**

**Was there any (suspicion of) infection since prostate biopsy?**

No *(if no, please continue at question 8)*

Yes, namely (multiple answers possible) *(if yes, please continue at question 5)*

Fever (body temperature of 38 °C or more) Infection of the prostate

Urinary tract infection Epididymitis

Renal pelvis inflammation

Otherwise, namely……………………………………………………………………………………

**Question 5. Have you been in contact with a doctor since prostate biopsy because of (suspected) infection?**

No

Yes, namely (multiple answer possible)

General practioner, namely…………………………….(name)…………………………...(place)

A urologist from the hospital where prostate biopsy was performed

A urologist from another hospital than where prostate biopsy was performed ……..……………

Otherwise, namely……………………………………………………………………………………

**Question 6. Have you been hospitalized since prostate biopsy because of (suspected) infection?**

No

Yes, namely………………………………………………………………………………(hospital name)

**Question 7. Did you have EXTRA medical appointmens since prostate biopsy because of (suspected) infection?** It concerns appointments with the general practitioner, general practitioner out-of-hours service or hospital which you would not have had without (suspicion) of infection.

No

Yes, I went to the doctor mentioned below on the following date(s) and location

If you don't remember exactly, please enter an approximate date (if possible)

Date:…………………………………… Location:……………………………………………..............

*Optional in case of multiple medical visits:*

Date:…………………………………… Location:……………………………………………..............

Date:…………………………………… Location:……………………………………………..............

**Question 8a. Did you use antibiotics since prostate biopsy (other than the antibiotics that we prescribed to prevent infection after prostate biopsy)?**

No *(if no, please continue at question 9)*

Yes, namely (multiple answers possible) *(if no, please continue at question 8b)*

Amoxicillin Pheneticillin (Broxil)

Amoxicillin//clavulanic acid (Augmentin) Flucloxacillin (Floxapen)

Azithromycin (Zitromax) Fosfomycin (Monuril)

Ciprofloxacin (Ciproxin) Moxifloxacin (Avelox)

Clindamycin (Dalacin) Metronidazole (Flagyl)

Clarythromycin (Klacid) Nitrofurantoin (Furabid)

Doxycycline Trimethoprim/sulfamethoxazole

Intravenous antibiotics

Otherwise, namely…………………………………………………………………………………

I do not know the name of the antibiotic(s)

**Vraag 8b. Have you experienced side effects or complaints related to the antibiotics?**

It does not concern side effects or complaints related to the antibiotics that we have prescribed to prevent an infection after prostate biopsy (prophylaxis)

No

Yes, namely……………………………………………………………………………………………………..

**This was the last question.**

Do you have any questions or comments? Please let us know in the comments section below.

……………………………………………………………………………………………………………………....................................................................................................................................................................................................................................................................................................................................................................................................................................................................................................................................................................

Questionnaire 3 (sent approximately 30 days post-biopsy)

**Explanation: please read this first!**

**What is the questionnaire about?**

This questionnaire is part of the medical research you participate in regarding culture-based prophylaxis in transrectal prostate biopsy. This questionnaire is about the use of antibiotics and the occurrence of infections after the prostate biopsy.

**How long does it take to complete the questionnaire?**

It will take approximately 15 minutes to complete the questionnaire.

**How to fill in the questionnaire?**

- You will receive three questionnaires. This is the last questionnaire. It is important to fill in the questionnaire completely. Even if you have already answered the question in the previous list.
- Start with the first question and follow the numbering.
- Tick one box for each question, unless stated else.
- For some questions, you can enter a number or something else on the dotted line.
- Fill in the questions based on your experience and opinion. There are no right or wrong answers here.


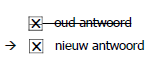
**Do you want to change an answer?**

- Cross out the old answer
- Tick a new answer
- Place an arrow in front of the new answer

**Can’t fill in the questionnaire yourself?**

If you can’t complete the list yourself, someone may be able to help you. For example, an family member.

**What happens with your answers?**

Your answers will be used for research. Only the researchers will see your answers, no one else. The investigators will not tell anyone that you participated in the trial.

**Thanks you for filling in the list for us!**

**The following questions are about the use of antibiotics, the occurrence of infections and hospitalization in the period in the past three weeks.**

**Research number:**

**Question 1. On which date did you complete this questionnaire?**

**Question 2. Recently you underwent a prostate biopsy.**

**Was there any (suspicion of) infection in the past three weeks?**

No *(if no, please continue at question 6)*

Yes, namely (multiple answers possible) *(if yes, please continue at question 3)*

Fever (body temperature of 38 °C or more) Infection of the prostate

Urinary tract infection Epididymitis

Renal pelvis inflammation

Otherwise, namely…………………………………………………………………………………

**Question 3. Have you been in contact with a doctor in the past three weeks because of (suspected) infection?**

No

Yes, namely (multiple answer possible)

General practioner, namely……………………………..(name)…………………………...(place)

A urologist from the hospital where prostate biopsy was performed

A urologist from another hospital than where prostate biopsy was performed ……..……………

Otherwise, namely…………………………………………………………………………………

**Question 4. Have you been hospitalized in the past three weeks because of (suspected) infection?**

No

Yes, namely………………………………………………………………………………(hospital name)

**Question 5. Did you have EXTRA medical appointmens in the past three weeks because of (suspected) infection?** It concerns appointments with the general practitioner, general practitioner out-of-hours service or hospital which you would not have had without (suspicion) of infection.

No

Yes, I went to the doctor mentioned below on the following date(s) and location

If you don't remember exactly, please enter an approximate date (if possible)

Date:…………………………………… Location:……………………………………………..............

*Optional in case of multiple medical visits:*

Date:…………………………………… Location:……………………………………………..............

Date:…………………………………… Location:……………………………………………..............

**Question 8a. Did you use antibiotics in the past three weeks (other than the antibiotics that we prescribed to prevent infection after prostate biopsy)?**

No *(if no, please continue at question 9)*

Yes, namely (multiple answers possible) *(if no, please continue at question 8b)*

Amoxicillin Pheneticillin (Broxil)

Amoxicillin//clavulanic acid (Augmentin) Flucloxacillin (Floxapen)

Azithromycin (Zitromax) Fosfomycin (Monuril)

Ciprofloxacin (Ciproxin) Moxifloxacin (Avelox)

Clindamycin (Dalacin) Metronidazole (Flagyl)

Clarythromycin (Klacid) Nitrofurantoin (Furabid)

Doxycycline Trimethoprim/sulfamethoxazole

Intravenous antibiotics

Otherwise, namely…………………………………………………………………………………

I do not know the name of the antibiotic(s)

**Vraag 8b. Have you experienced side effects or complaints related to the antibiotics?**

It does not concern side effects or complaints related to the antibiotics that we have prescribed to prevent an infection after prostate biopsy (prophylaxis)

No

Yes, namely…………………………………………………………………………………………………

**This was the last question.**

Do you have any questions or comments? Please let us know in the comments section below.

**Supplementary File IV: Microbiological outcomes**

Pre-biopsy rectal culture

Our phenotypic culture method obviated full susceptibility testing of all cultured colonies, so that culture results were available within 48 hours in 92% of the patients, which was important for urologists in previous research on the implementation of culture-based prophylaxis.(16). The timing between rectal swab collection and prostate biopsy is depicted in Supplementary Figure I.

**Supplementary Figure I.** Days between rectal swab collection and prostate biopsy

**Days before prostate biopsy**

**Frequency**

Growth rates of relevant bacteria on the different agars can be found in Supplementary Table I. In case of growth on the agar containing ciprofloxacin, on average 1.2 unique bacteria with different morphology were cultured. The most common bacteria cultured on the agar with ciprofloxacin were Escherichia coli (89.7%), followed by Klebsiella species (4.3%), Proteus species (2.1%) and Pseudomonas aeruginosa (2.1%).

| **Supplementary Table I.** Growth rates of relevant bacteria on the different agars (n=1524) | |
| --- | --- |
| MacConkey agar + 0.5 mg/l ciprofloxacin (McCIP) | 15.4% |
| MacConkey agar + 2 mg/l trimethoprim (McTMP) | 52.8% |
| MacConkey agar + 4 mg/l fosfomycin (McFOS) | 62.5% |
| MacConkey agar + 2 mg/l mecillinam + 8 mg/l amoxicillin/clavulanic acid (McMECAMC) | 44.6% |

Based on the results of our study, in a culture-based strategy with ciprofloxacin, in 84.6% of the patients (n=1290) ciprofloxacin could be prescribed. In 15.4% of the patients (n=234), alternative prophylaxis was required. In half of these patients (7.7%; n=117), another single oral prophylactic agent could be prescribed. In the other half (7.7%; n=117), a combination of oral antibiotics regimens or intravenous antibiotics were required. The number of single oral alternative antibiotics agents available in case of growth on the agar with ciprofloxacin can be found in Supplementary Table II.

| **Supplementary Table II. N**umber of single oral alternative antibiotics agents available in case of growth on the agar with ciprofloxacin | | | |
| --- | --- | --- | --- |
| No relevant growth on the other 3 agars | 10.7% | 3 alternatives available | 10.7% |
| McTMP only | 8.1% | 2 alternatives available | 14.0% |
| McFOS only | 3.8% |  |  |
| McMECAMC only | 2.1% |  |  |
| McTMP + McFOS | 12.4% | 1 alternative available | 25.2% |
| McTMP + McMECAMC | 6.4% |  |  |
| McFOS + McMECAMC | 6.4% |  |  |
| McTMP + McFOS + MCMECAMC | 50.0% | No alternative with a single oral antibiotic available | 50.0% |

In a culture-based strategy without ciprofloxacin, in 1059 patients (69.5%) a single oral prophylactic agent could be prescribed. In the other 465 patients (30.5%), a combination of oral antibiotics regimens or intravenous antibiotics were required.

Post-biopsy cultures (taken because of infectious complications)

Combining all culture proven infections i.e. blood- and urine cultures, 2.9% (n=19) of the patients in the CG had an early culture-proven infection compared to 0.8% (n=5) of the patients in the IG (reduction -2.1%; 95% CI 0.005 to 0.038). In total, 3.8% (n=25) (CG) and 1.7% (n=11) (IG) of the patients developed a culture-proven infection within 30 days post-biopsy (reduction -2.1%; 95% CI 0.002 to 0.041).

| **Supplementary table III. Microbiological outcomes of patients with an infectious complication within seven days post-biopsy (primary outcome)** | | | | | |
| --- | --- | --- | --- | --- | --- |
| **Patient number** | **Ciprofloxacin resistant bacteria cultured (Eswab 1)** | **Antibiotic prophylaxis used** | **Type of infectious complication** | **Causative pathogen of infection isolated** | **Ciprofloxacin Sensitive or Resistant** |
| Culture-based prophylaxis (IG) | | | | | |
| 120315 | No | Ciprofloxacin | Isolated fever | No microbiological culture performed | NA |
| 140019 | No | Ciprofloxacin | Definitive UTI with systemic symptoms | Urine culture: negative  Blood culture: negative | NA |
| 140022 | No | Ciprofloxacin | Probable UTI without systemic symptoms | No microbiological culture performed | NA |
| 140033 | No | Ciprofloxacin | Definitive UTI with systemic symptoms | Urine culture: *E.coli*  Blood culture: not performed | R |
| 150052 | No | Ciprofloxacin | Isolated fever | Urine culture: negative  Blood culture: negative | NA |
| 170021 | Yes, *E. coli* | Fosfomycin | Definitive UTI with systemic symptoms | Urine culture: negative  Blood culture: negative | NA |
| 170046 | No | Ciprofloxacin | Definitive UTI with systemic symptoms | Urine culture: *E.coli*  Blood culture: not performed | S |
| 170117 | No | Ciprofloxacin | Definitive UTI with systemic symptoms | Urine culture: negative  Blood culture: negative | NA |
| 170118 | No | Ciprofloxacin | Definitive UTI with systemic symptoms | Urine culture: *E.coli + P. rettgeri*  Blood culture: *E.coli* | I |
| 180051 | No | Ciprofloxacin | Definitive UTI without systemic symptoms | Urine culture: *E.coli*  Blood culture: not performed | R |
| 180125 | No | Ciprofloxacin | Probable UTI with systemic symptoms | Urine culture: negative  Blood culture: negative | NA |
| 180156 | Yes, *E. coli* | Pivmecillinam + amoxicillin clavulanic acid | Definitive UTI with systemic symptoms | Urine culture: negative  Blood culture: *S. hominis* (contaminant) | NA |
| 180198 | No | Ciprofloxacin | Probable UTI without systemic symptoms | No microbiological culture performed | NA |
| 210002 | No | Ciprofloxacin | Definitive UTI without systemic symptoms | No microbiological culture performed | NA |
| 230007 | No | Ciprofloxacin | Probable UTI with systemic symptoms | Urine culture: negative  Blood culture: negative | NA |
| 230015 | No | Ciprofloxacin | Definitive UTI with systemic symptoms | Urine culture: *E.coli*  Blood culture: negative | S |
| **Patient number** | **Ciprofloxacin resistant bacteria cultured (Eswab 1)** | **Antibiotic prophylaxis used** | **Type of infectious complication** | **Causative pathogen of infection isolated** | **Ciprofloxacin Sensitive or Resistant** |
| Empirical prophylaxis with ciprofloxacin | | | | | |
| 120033 | No | Ciprofloxacin | Isolated fever | No microbiological culture performed | NA |
| 120064 | Yes, *E. coli* | Ciprofloxacin | Definitive UTI without systemic symptoms | Urine culture: *C. kerstersii*  Blood culture: not performed | R |
| 120080 | No | Ciprofloxacin | Definitive UTI with systemic symptoms | Urine culture: *K. pneumoniae*  Blood culture: negative | S |
| 120117 | No | Ciprofloxacin | Acute epididymitis | Urine culture: not performed  Blood culture: negative | NA |
| 120231 | Yes, *E. coli* | Ciprofloxacin | Definitive UTI without systemic symptoms | No microbiological culture performed | NA |
| 120324 | No | Ciprofloxacin | Definitive UTI with systemic symptoms | Urine culture: not performed  Blood culture: *K. pneumoniae* | S |
| 130002 | Yes, *E. coli* | Ciprofloxacin | Definitive UTI with systemic symptoms | Urine culture: *E.coli*  Blood culture: negative | R |
| 140025 | Yes, *E. coli* | Ciprofloxacin | Definitive UTI with systemic symptoms | Urine culture: *E.coli*  Blood culture: not performed | R |
| 160033 | No | Ciprofloxacin | Isolated fever | Urine culture: negative  Blood culture: not performed | NA |
| 160045 | No | Ciprofloxacin | Definitive UTI with systemic symptoms | Urine culture: *E.coli*  Blood culture: *E.coli* | S |
| 170062 | No | Ciprofloxacin | Sepsis | Urine culture: negative  Blood culture: *E.coli* | S |
| 170084 | Yes, *E. coli* | Ciprofloxacin | Acute epididymitis | Urine culture: *E.coli*  Blood culture: not performed | R |
| 170100 | Yes, *E. coli* | Ciprofloxacin | Definitive UTI with systemic symptoms | Urine culture: *E.coli*  Blood culture: *E.coli* | R |
| 170111 | No | Ciprofloxacin | Definitive UTI with systemic symptoms | Urine culture: *E.coli + E. faecalis*  Blood culture: negative | *E.coli S*  *E. faecalis* R |
| 170122 | Yes, *E. coli* | Ciprofloxacin | Definitive UTI with systemic symptoms | Urine culture: *E.coli*  Blood culture: not performed | R |
| 170125 | Yes, *E. coli* | Ciprofloxacin | Definitive UTI with systemic symptoms | Urine culture: negative  Blood culture: not performed | NA |
| **Patient number** | **Ciprofloxacin resistant bacteria cultured (Eswab 1)** | **Antibiotic prophylaxis used** | **Type of infectious complication** | **Causative pathogen of infection isolated** | **Ciprofloxacin Sensitive or Resistant** |
| 170137 | No | Ciprofloxacin | Isolated fever | Urine culture: not performed  Blood culture: negative | NA |
| 170140 | Yes, *E. coli* | Ciprofloxacin | Definitive UTI with systemic symptoms | Urine culture: negative  Blood culture: *E.coli* | R |
| 170174 | No | Ciprofloxacin | Acute prostatitis | Urine culture: *E.coli*  Blood culture: not performed. | S |
| 170187 | Yes, *E. coli* | Ciprofloxacin | Isolated fever | Urine culture: negative  Blood culture: negative | NA |
| 180122 | No | Ciprofloxacin | Definitive UTI with systemic symptoms | Urine culture: negative  Blood culture: *E.coli* | R |
| 190025 | No | Ciprofloxacin | Probable UTI with systemic symptoms | Urine culture: negative  Blood culture: negative | NA |
| 210019 | Yes, *E. coli* | Ciprofloxacin | Definitive UTI with systemic symptoms | Urine culture: *E.coli*  Blood culture: *E.coli* | I |
| 220003 | Yes, *E. coli* | Ciprofloxacin | Definitive UTI with systemic symptoms | Urine culture: *E. coli*  Blood culture: negative | I |
| 220013 | Yes, *E. coli* | Ciprofloxacin | Probable UTI with systemic symptoms | Urine culture: negative  Blood culture: not performed | NA |
| 250003 | No | Ciprofloxacin | Definitive UTI with systemic symptoms | Urine culture: *E. coli*  Blood culture: negative | I |
| 250004 | Yes, *E. coli* | Ciprofloxacin | Definitive UTI with systemic symptoms  Acute epididymitis | Urine culture: *E. coli*  Blood culture: negative | R |
| 250006 | Yes, *E. coli* | Ciprofloxacin | Definitive UTI with systemic symptoms | Urine culture: *E.coli*  Blood culture: *E.coli* | I |

| **Supplementary table III. Microbiological outcomes of patients with an infectious complication between eight and 30 days post-biopsy (secondary outcome)** | | | | | |
| --- | --- | --- | --- | --- | --- |
| **Patient number** | **Ciprofloxacin resistant bacteria cultured (Eswab 1)** | **Antibiotic prophylaxis used** | **Type of infectious complication** | **Causative pathogen of infection isolated** | **Ciprofloxacin Sensitive or Resistant** |
| Culture-based prophylaxis (IG) | | | | | |
| 120036 | No | Ciprofloxacin | Definitive UTI without systemic symptoms | Urine culture: *E.coli*  Blood culture: not performed | S |
| 120099 | No | Ciprofloxacin | Definitive UTI without systemic symptoms | Urine culture: negative  Blood culture: not performed | NA |
| 120177 | Yes, Klebsiella spp. | Pivmecillinam + amoxicillin clavulanic acid | Definitive UTI with systemic symptoms | Urine culture: negative  Blood culture: not performed | NA |
| 120213 | Yes, *E. coli* | Fosfomycin | Definitive UTI without systemic symptoms | Urine culture: *E.coli*  Blood culture: not performed | I |
| 120353 | No | Ciprofloxacin | Acute prostatitis | Urine culture: negative  Blood culture: not performed | NA |
| 120394 | No | Ciprofloxacin | Definitive UTI without systemic symptoms | Urine culture: *Aerococcus urinae*  Blood culture: not performed | S |
| 120445 | No | Ciprofloxacin | Probable UTI without systemic symptoms | No microbiological culture performed | NA |
| 170058 | No | Ciprofloxacin | Isolated fever | No microbiological culture performed | NA |
| 170070 | No | Ciprofloxacin | Probable UTI with systemic symptoms | No microbiological culture performed | NA |
| 170088 | No | Ciprofloxacin | Probable UTI without systemic symptoms | No microbiological culture performed | NA |
| 180032 | No | Ciprofloxacin | Definitive UTI with systemic symptoms | Urine culture: *E.coli*  Blood culture: negative | S |
| 180054 | Yes, *E. coli* | Ciprofloxacin | Definitive UTI without systemic symptoms | Urine culture: *E.coli*  Blood culture: not performed | R |
| 180150 | No | Ciprofloxacin | Definitive UTI with systemic symptoms | Urine culture: *S. saprophyticus*  Blood culture: not performed | S |
| 230028 | No | Ciprofloxacin | Acute epididymitis | Urine culture: negative  Blood culture: not performed | NA |
| Empirical prophylaxis with ciprofloxacin | | | | | |
| 120073 | Yes, *E. coli* | Ciprofloxacin | Definitive UTI without systemic symptoms | Urine culture: *E.coli*  Blood culture: not performed | R |
| **Patient number** | **Ciprofloxacin resistant bacteria cultured (Eswab 1)** | **Antibiotic prophylaxis used** | **Type of infectious complication** | **Causative pathogen of infection isolated** | **Ciprofloxacin Sensitive or Resistant** |
| 120097 | Yes, *E. coli* | Ciprofloxacin | Definitive UTI with systemic symptoms | Urine culture: Enterococcus spp.  Blood culture: not performed | R |
| 120133 | No | Ciprofloxacin | Definitive UTI with systemic symptoms | Urine culture: *E.coli*  Blood culture: not performed | S |
| 120188 | No | Ciprofloxacin | Probable UTI with systemic symptoms | Urine culture: negative  Blood culture: not performed | NA |
| 120222 | Yes, *E. coli* | Ciprofloxacin | Definitive UTI with systemic symptoms | Urine culture: *K. pneumoniae*  Blood culture: *K. pneumoniae* | S |
| 120290 | No | Ciprofloxacin | Definitive UTI without systemic symptoms | Urine culture: Enterococcus spp.  Blood culture: not performed | R |
| 130023 | Yes, *E. coli* | Ciprofloxacin | Acute epididymitis | Urine culture: negative  Blood culture: not performed | NA |
| 220010 | No | Ciprofloxacin | Definitive UTI without systemic symptoms | No microbiological culture performed | NA |
| 230046 | No | Ciprofloxacin | Definitive UTI without systemic symptoms | Urine culture: Enterococcus spp.  Blood culture: not performed | R |

**Supplementary File V:** **Differences in effect between hospitals**

We consider our heterogeneous study population a strength of the study as it reflects ‘real life’ and as such leads to better generalizability. There is, however, considerable variation in the number of patients included in each hospital. To account for any cluster effect in our study, stratified randomisation and stratified analysis for hospital was performed. Given the low event rate of our primary outcome measure, differences in effect between the hospitals can only be reliably estimated for hospitals with the highest inclusion rates (B and G).

| **Differences in the risk of infectious complications within seven days post-biopsy between the control- and the intervention group for hospital B and G** | | | | | |
| --- | --- | --- | --- | --- | --- |
| **Hospital** | **Total** | **Control group** | **Intervention group** | **Confidence interval for the difference between both groups** | **Growth on the agar with ciprofloxacin** |
| Hospital B | 1.8% (n=399) | 2.9% (n=206) | 0.5% (n=193) | -2.4%; 95% CI -0.009 to 0.060 | 14.5% (n=399) |
| Hospital G | 5.4% (n=350) | 6.2% (n=179) | 4.7% (n=171) | -1.5%; 95% CI -0.040 to 0.069 | 13.4% (n=350) |
